# Repurposing the Pathogen Box compounds for identification of potent anti-malarials against blood stages of *Plasmodium falciparum* with PfUCHL3 inhibitory activity

**DOI:** 10.1038/s41598-021-04619-4

**Published:** 2022-01-18

**Authors:** Hina Bharti, Aakriti Singal, Manisha Saini, Pradeep Singh Cheema, Mohsin Raza, Suman Kundu, Alo Nag

**Affiliations:** Department of Biochemistry, University of Delhi South Campus, Benito Juarez Road, New Delhi, 110021 India

**Keywords:** Biochemistry, Computational biology and bioinformatics, Drug discovery, Bioinformatics, High-throughput screening, Isolation, separation and purification

## Abstract

Malaria has endured as a global epidemic since ages and its eradication poses an immense challenge due to the complex life cycle of the causative pathogen and its tolerance to a myriad of therapeutics. PfUCHL3, a member of the ubiquitin C-terminal hydrolase (UCH) family of deubiquitinases (DUBs) is cardinal for parasite survival and emerges as a promising therapeutic target. In this quest, we employed a combination of computational and experimental approaches to identify PfUCHL3 inhibitors as novel anti-malarials. The Pathogen Box library was screened against the crystal structure of PfUCHL3 (PDB ID: 2WE6) and its human ortholog (PDB ID: 1XD3). Fifty molecules with better comparative score, bioavailability and druglikeliness were subjected to in-vitro enzyme inhibition assay and among them only two compounds effectively inhibited PfUCHL3 activity at micro molar concentrations. Both MMV676603 and MMV688704 exhibited anti-plasmodial activity by altering the parasite phenotype at late stages of the asexual life cycle and inducing the accumulation of polyubiquitinated substrates. In addition, both the compounds were non-toxic and portrayed high selectivity window for the parasite over mammalian cells. This is the first comprehensive study to demonstrate the anti-malarial efficacy of PfUCHL3 inhibitors and opens new avenues to exploit UCH family of DUBs as a promising target for the development of next generation anti-malaria therapy.

## Introduction

Malaria is a globally acknowledged parasitic disease that causes an enormous socio-economic burden worldwide. Despite endless preventive measures, it continues to impact millions of people across the regions of sub-Saharan Africa and Asia, impelling thousands of deaths annually^1^. It is caused by six species of the unicellular eukaryotic pathogen of the genus *Plasmodium* and is mainly transmitted by the bite of an infected female *Anopheles* mosquito. *P. falciparum* being the deadliest among all the malaria causing *Plasmodium* species and accounts for the highest mortality rate^2^. Presently, an orchestra of sensitive diagnostic tools, effective chemotherapeutics, and vector control strategies have contributed immensely to limit the prevalence of this disease^3–5^. Undoubtedly, Artemisinin-based combination therapy (ACT) is the most effective treatment for malaria and accounts for reduced mortality. However, a major setback in the treatment of this disease appears with the occurrence of Artemisinin resistant *Plasmodium* strain in Thailand–Cambodia borders^6,7^. The persistence of resistance against other partnered non-clinical drugs with associated toxicity, limits the responsiveness of these drugs during the treatment regimen^8–11^. Furthermore, Mosquirix, the only licensed first-generation RTS, S/AS01 vaccine exhibits limited protection with an efficacy rate of only thirty percent^12,13^. Altogether, the dearth of effective vaccines and potent drugs against malaria warrants an absolute urgency to identify novel therapeutics with distinctive mode of action and molecular targets.

Surprisingly, unlike other eukaryotes, *Plasmodium* species own few transcription regulators^14,15^, thereby, ascertaining the functional relevance of post translational modification (PTM) modulators in the parasite life cycle. Amidst varied PTMs, ubiquitination is an evolutionarily conserved class of PTMs that serves versatile cellular purposes in different eukaryotic organisms, including *Plasmodium*^16^. The ubiquitous involvement of this cascade in nearly every cellular and biological process enables the parasite for an elaborated adaptation to the staggeringly variable cellular environment that the parasites encounter across their multistage life cycle^17,18^. Moreover, a major proportion of parasite proteome is identified as the potential target for ubiquitination, and these ubiquitin tagged substrate are detected across all the asexual stages of the parasite life cycle^16^. Several knockout and experimental inhibition studies have identified various members of this pathway to be of clinical significance^19–21^. Owing to such essential character, the craftsmen of this pathway represent an attractive drug target for the development of newer antimalarials. Ubiquitin modification is carried out by sequential interplay of three enzymes namely E1, E2, and E3, whereas, the process is reversed by a highly specialized family of enzymes known as deubiquitinases (DUBs)^22^. DUBs are a group of proteases that removes the ubiquitin moiety from substrates by cleaving isopeptide linkages at the C-terminus of ubiquitin and regulates a plethora of cellular processes such as ubiquitin homeostasis, apoptosis, cell cycle progression, stress response, cell signalling and DNA damage, among others^23–27^. Any aberration in DUBs regulated pathways is associated with severe implications in various diseases such as cancer, inflammatory and neurodegenerative diseases^28–31^. Not surprisingly, DUBs are even identified as the key modulators of unicellular parasitic protozoans, therefore, they may serve as an attractive therapeutic target in several infectious diseases^32–35^. Likewise, several proteasome inhibitors such as Salinosporamide^36^, Bortezomib^37^, Epoxomicin^38^ and MLN-273^39^ have been shown to inhibit the growth and development of the malarial parasite. Moreover, resistance to Artemisinin is linked with the missense mutation in a gene encoding a deubiquitinase (UBP-1) on chromosome 2 in *P. chabaudi* Artemisinin resistant strain AS-ATN^40–42^. Together, these studies render DUBs as promising candidates for targeted therapeutic development against malaria.

*Plasmodium falciparum* encodes well-defined deubiquitination machinery with 29 DUBs and DUB-like proteins^43^, classified as cysteine protease [ubiquitin-specific processing proteases (USP/UBP), ovarian tumor domain-containing proteases (OTU), Machado-Josephin domain (MJD) DUBs, ubiquitin C-terminal hydrolases (UCH)] and zinc-metalloprotease [JAMM/MPN motif proteases (JAMM)]^44^. Among these, members of the cysteine protease family of DUBs have been identified as crucial mediators of the parasite life cycle. For instance, inhibition of PfUSP14 results in parasite death by altering the cellular ubiquitin homeostasis^45^. Whereas, PfOTU maintains parasite apicoplast by associating with Atg8^35^. Overexpression of a functional mutant of PfUCHL3 exerts a dominant-negative effect on wild type parasites, thereby, highlighting its inevitable role in parasite survival^46^. Among known DUBs in *P. falciparum*, UCHL3 is a structurally characterized deubiquitinase exhibiting dual activity and is a validated drug target. Whereas, in eukaryotes, UCHL3 is known to regulate numerous cellular pathways including oocyte maturation^47^, DNA damage sensing and repair^48,49^ and stress management^50^. Recently, it has emerged as an anti-cancer^51–53^ and anti-neurodegenerative^54^ therapeutic target as well. Surprisingly, despite the evolutionarily conserved biochemical traits, PfUCHL3 exhibits modest similarity to *Homo sapiens* UCHL3 (HsUCHL3) and displays considerable architectural variation in residues defining interaction in the active site pocket^55^. In comparison to human ortholog, the residues lining the binding groove of PfUCHL3 exhibits notable differences, which facilitate the formation of completely distinct interactions with its substrate. These interactions aid PfUCHL3 to attain a different conformation from its mammalian ortholog. Notably, the catalytic triad of PfUCHL3 lies within the milieu of functional interaction and undergoes minor conformational changes upon substrate binding as compared to HsUCHL3 whose active site is occluded by loop and undergo major changes upon substrate binding^46^. Hence, such differential structural characteristics can be exploited for the identification of pathogen selective inhibitors. Several studies employing structure based drug design approach have been reported for the identification of potent yet selective anti-malarials^56–58^. In this pursuit, we hypothesized that inhibitors targeting PfUCHL3 will possibly act as potent therapeutics in malaria therapy. However, developing a novel pharmacological agent is a time-consuming, extremely costly and high-risk process^59^. On the contrary, drug repurposing is a powerful alternative approach to overcome the above challenges^60^.

Drug repurposing (even known as repositioning, reprofiling or re-tasking) is a process for identification of new therapeutic potential of an existing drug (approved/investigated against different diseases)^61^. With this aim, Medicines of Malaria Ventures (MMV) assembled and released Pathogen Box in 2015, consisting of 400 pure compounds active against Type II and III diseases^62^. Based on their activity, these compounds are grouped into varied pathogen specific categories^63^. Several studies have repurposed the Pathogen Box compounds against an array of diseases to identify novel inhibitors that provide a rudimentary scaffold as a primitive tool for drug discovery^62–67^. Therefore, these studies intrigued us to repurpose MMV Pathogen Box compounds for the identification of novel PfUCHL3 inhibitors that may act as potent anti-malarials.

In this study, we employed structure-based drug discovery approach to repurpose 400 compounds from MMV Pathogen Box as novel anti-malarial with PfUCHL3 inhibitory effect. Herein, we docked Pathogen Box compounds against a grid encompassing active site residues and neighboring residues of PfUCHL3 and HsUCHL3. Based on comparative dock score and ADME (absorption, distribution, metabolism and excretion) profiling, best fifty candidates were assessed against in-vitro activity of *P. falciparum* UCHL3. Further, in-silico approaches were employed to gain insight into the enzyme-inhibitor interactions and the efficacy of the most promising hits was evaluated against the blood stages of *P. falciparum* in culture. The selectivity of lead compounds for parasites over the mammalian cell lines was also assessed. We present the first report attempting to identify the novel inhibitors targeting PfUCHL3 with potent inhibition of parasite growth. In the process, this study provides new chemical scaffolds that may serve as a template to develop more specific and potent clinical therapeutics against malaria.

## Results

### Structure based virtual screening (SBVS)

**Figure 1:**
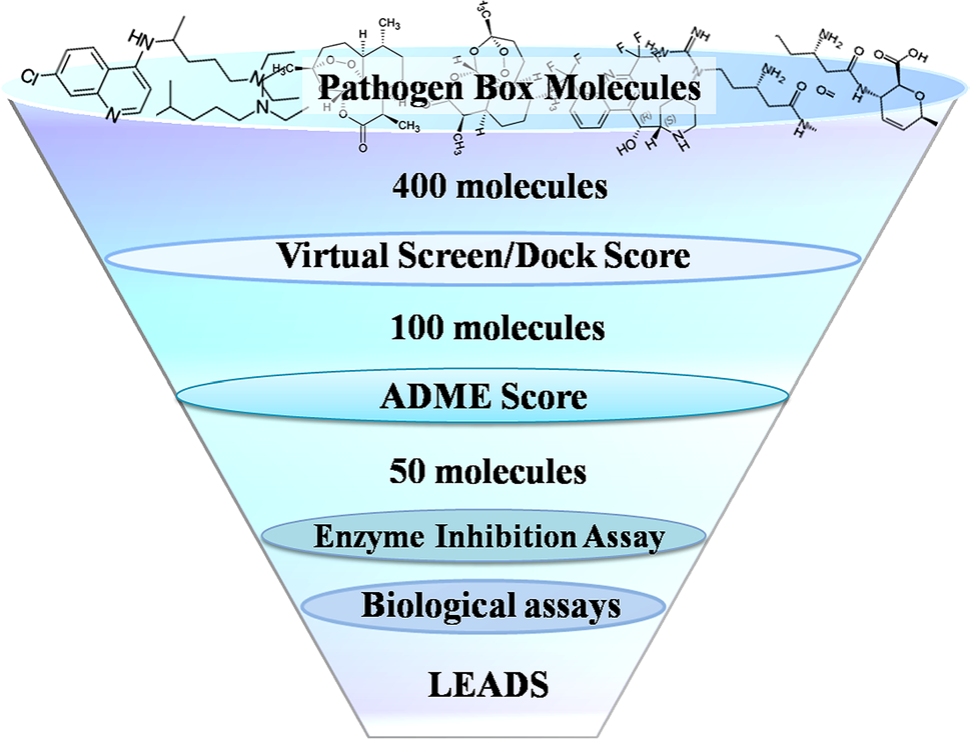
Schematic representation of the methodology for identification of potent hits against PfUCHL3: a flowchart illustrating the series of in-silico and in-vitro protocols employed to identify potent hits.

SBVS approach was employed to identify novel anti-PfUCHL3 molecules. A flow chart depicting the screening methodology is illustrated in Fig. 1. Molecular docking algorithms were used to screen 400 compounds against the X-ray crystallographic structure of the enzyme (PDB ID: 2WE6) and with its human correspondent (PDB ID: 1XDT), which shares only 30% identity. Each molecule was docked 50 times in different conformations at the active site cleft of both the enzymes and the top hundred molecules were picked on the basis of higher selectivity towards *P. falciparum* UCHL3 than its mammalian counterpart (Table S1).

Next, the compounds exhibiting drug like properties, i.e. with acceptable range of pharmaceutically relevant properties and with zero violation of Lipinski rule of five were selected. Pharmaceutically relevant properties such as the partition coefficient (log Po/w) critical for estimation of absorption of drugs within the body, log Kp predicting skin permeability and log S exhibiting water solubility of the compounds were assessed, and eventually Pan-assay interference compounds which give false positive biological output were rejected^68^. As presented in Table S2, among the set of hundred compounds, based on SWISSDOCK-based ADME predictions, fifty distinct chemical entities that entirely fit inside the pink zone region of the bioavailability radar that displays the rapid appraisal of drug likeliness, were further investigated for their activity on recombinant PfUCHL3 in vitro.

### Expression, purification and in-vitro assessment of compounds for enzyme inhibition

**Figure 2:**
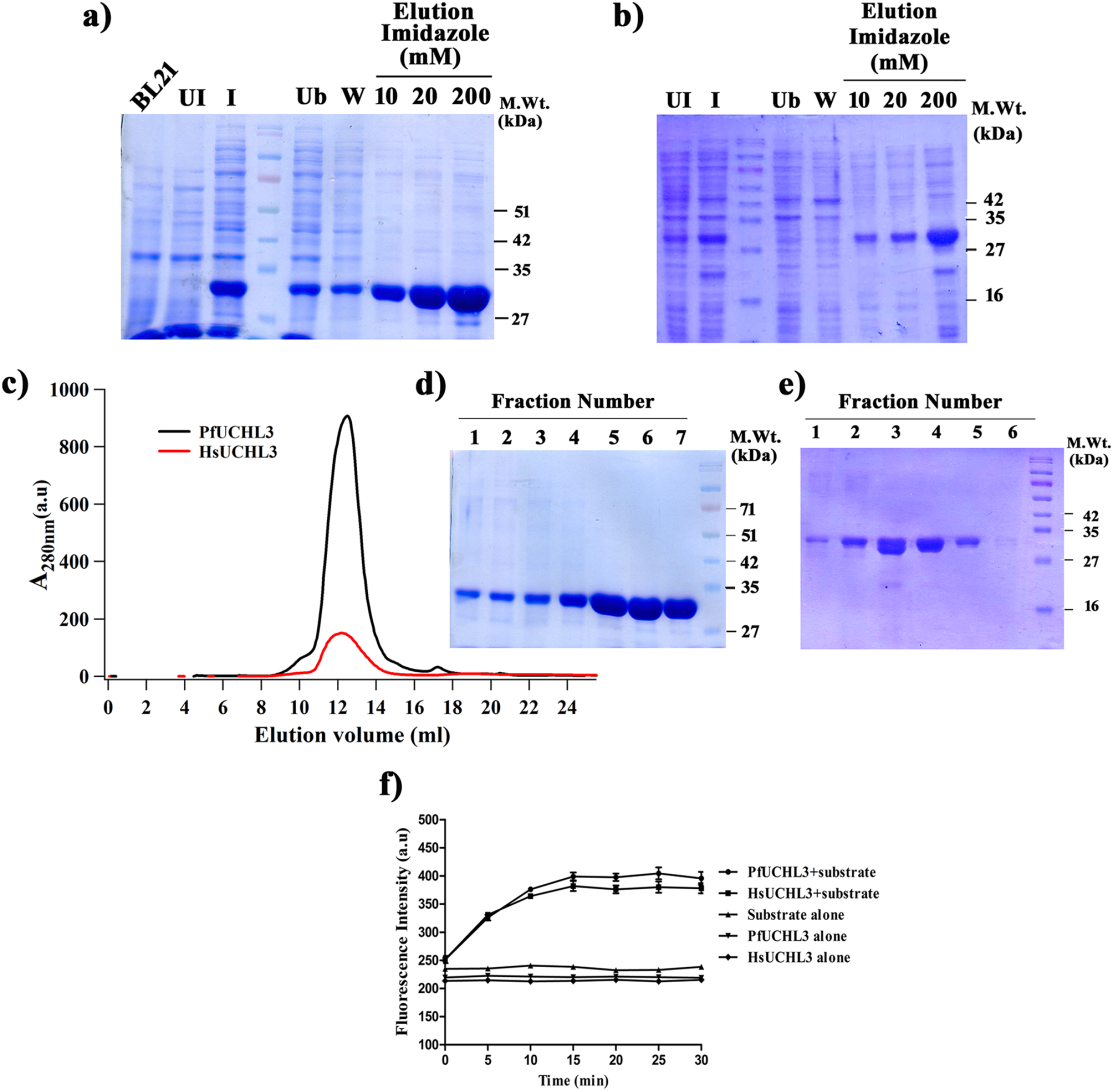
Expression, purification and deubiquitinating activity of PfUCHL3 and HsUCHL3: (**a**) PfUCHL3 and (**b**) HsUCHL3 were expressed in BL21 (λDE3) and purified using Ni-affinity chromatography, UI-uninduced, I-induced, Ub-unbound, W-wash. The proteins were further purified by gel filtration chromatography using AKTA prime. (**c**) The FPLC profile denotes a single peak for both the enzymes. Both (**d**) PfUCHL3 and (**e**) HsUCHL3 were purified to homogeneity as determined by SDS-PAGE analysis. (**f**) The catalytic activities of both the enzymes were assessed by measuring the change in fluorescence intensity, substrate and enzyme alone were taken as negative control. Uncropped images of commassie stained SDS gels are provided in Supplementary Fig. S1.

**Figure 3:**
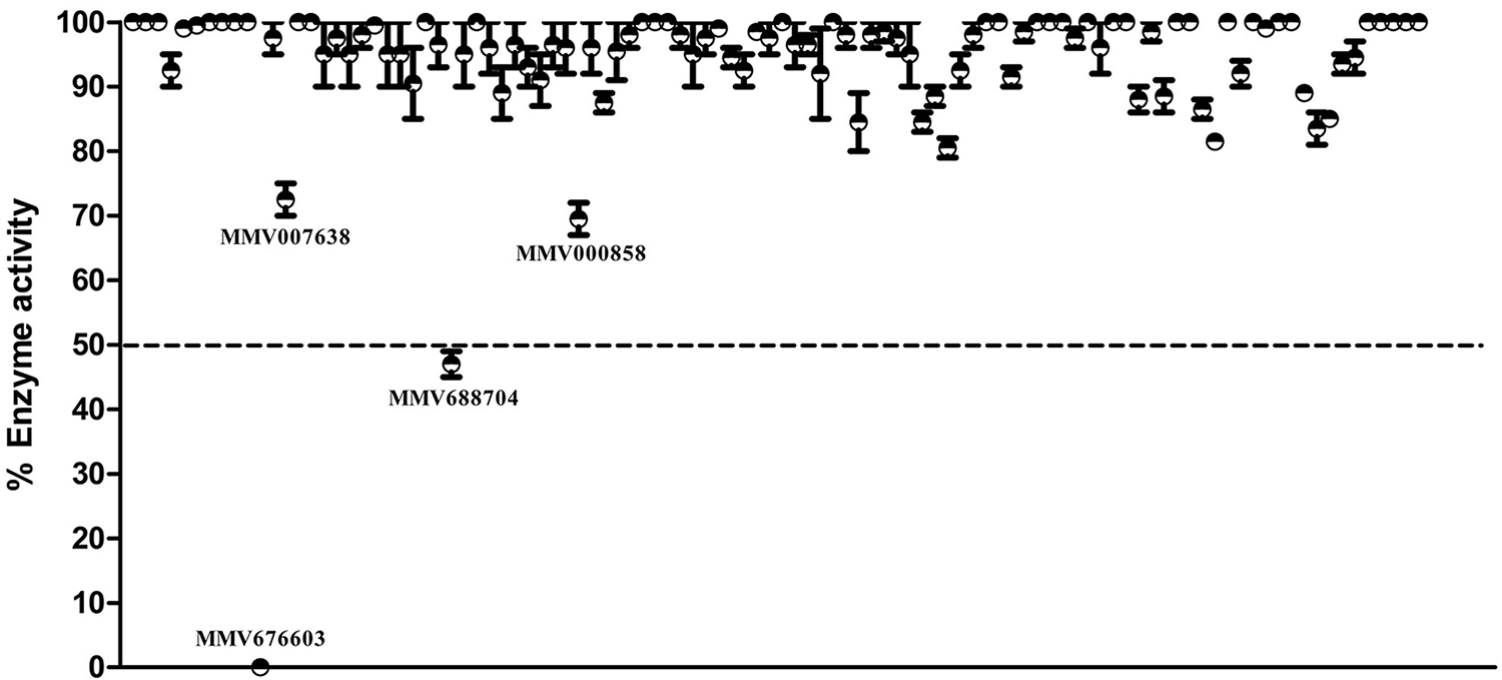
In-vitro screening of best hundred hits against PfUCHL3 activity: a scatter plot representing percent enzyme activity inhibition of fifty promising Pathogen Box compounds, identified from computational evaluation at 100 μM concentration. The dashed line at 50 is considered as inhibitory cutoff. Compounds with more than 50% inhibition threshold were considered for further screening. Bar denotes the standard deviation (n = 3) for each compound.

*Plasmodium falciparum* and human UCHL3 were expressed and purified to homogeneity as a single band around 30 kDa on SDS-PAGE (Fig. 2a–e). Both the enzymes were found to be catalytically active as evaluated by deubiquitinase assay (Fig. 2f). Subsequently, we evaluated the performance of the potential fifty compounds identified from *in-silico* studies against the catalytically active enzyme at a concentration of 100 μM with NEM (a known inhibitor of cysteine protease) as a positive internal control. At this concentration, the majority of compounds were ineffective on PfUCHL3 activity, whereas, four compounds showed more than thirty percent inhibition and only two of them (MMV676603 and MMV688704) exhibited an inhibition rate of more than fifty percent, as shown in Fig. 3.

**Figure 4:**
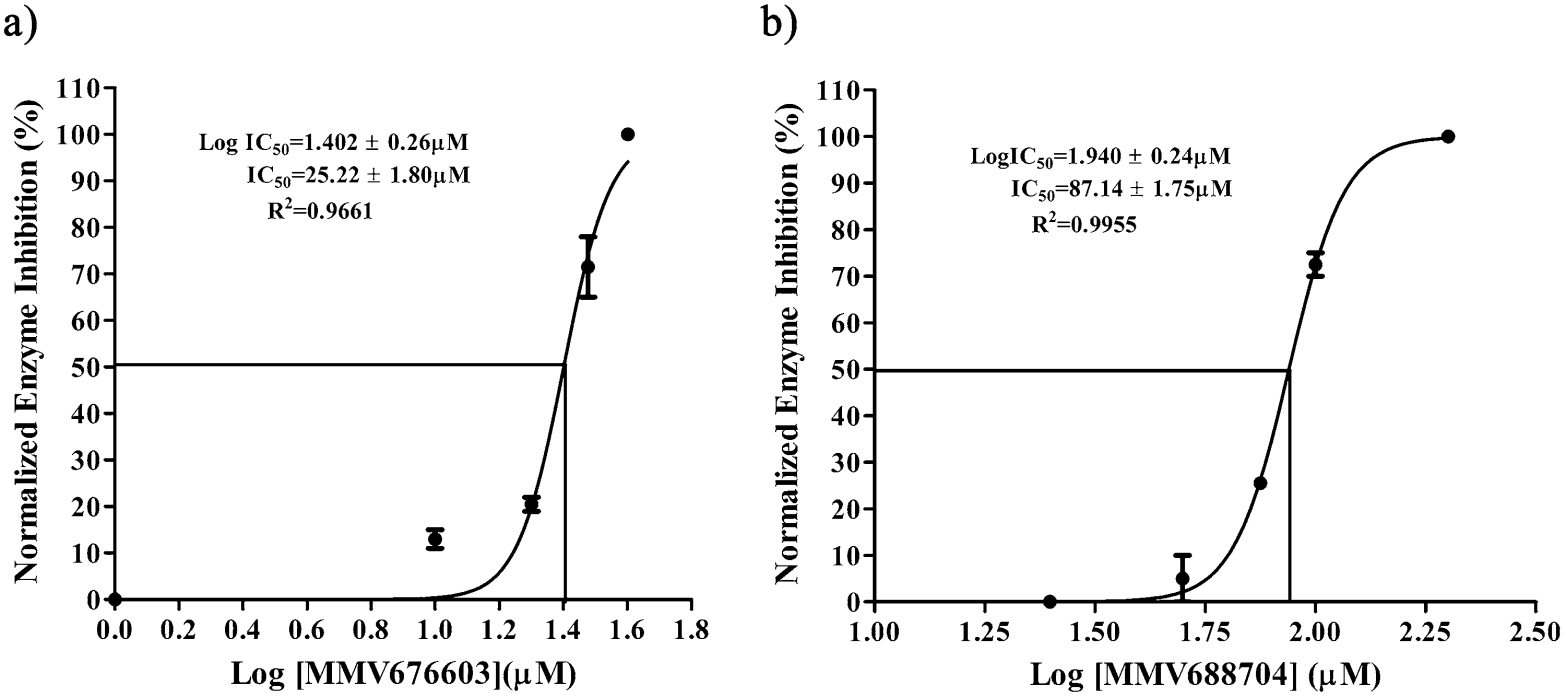
IC_50_ determination of the compounds MMV676603 and MMV688704 against PfUCHL3 activity: dose–response curve indicating the Log IC_50,_ IC_50_ and R^2^ values of (**a**) MMV676603 and (**b**) MMV688704 against recombinant PfUCHL3. Inhibition at each data point is depicted as Mean ± S.D. of three independent experiments.

Compounds with more than 50% enzyme inhibition were further evaluated for their inhibitory potential in a dose-dependent manner. As shown in Fig. 4a,b, compounds MMV676603and MMV688704 displayed an IC_50_ value of 25.12 ± 1.80 μM and 87.14 ± 1.75 μM, respectively. Concomitantly, due to the limited availability of the compounds, we evaluated these compounds only at their respective IC_50_ values against closely related human ortholog and observed no discernable effect on HsUCHL3 activity. The findings allude to the fact that both MMV676603 and MMV688704 hold the potential to inhibit in-vitro activity of PfUCHL3 over its mammalian ortholog with MMV676603 being a more potent inhibitor.

### Molecular docking analysis of the lead compounds

Molecular docking provides the best-fit pose of the ligand in the protein structure that allows the explicit analysis of protein–ligand interactions. Accordingly, compounds MMV676603 and MMV688704 showed the binding energies of − 7.23 and − 6.15 kcal/mol, respectively for PfUCHL3. In contrary, both MMV676603 and MMV688704 lack specific binding to human UCHL3 and displayed the binding energies of 0.18 and − 1.6 kcal/mol respectively (Table 1). Docking analysis provides the predicted IC_50_ values for MMV676603 and MMV688704 against PfUCHL3 as 4.99 and 30.91 μM, respectively. In sharp contrast, the predicted IC_50_ value for MMV676603 against HsUCHL3 was not defined because of its positive binding energy value, for e.g. 0.8, however, for MMV688704, it was found to be 67.09 mM, as summarized in Table 1. Together, these results imply that compounds MMV676603 and MMV688704 harness more specific inhibitory action on PfUCHL3 than its mammalian ortholog.

**Table 1:** Table presents the comparative binding energies and predicted IC_50_ values of the identified hits by Autodock software.

Name of the compound	△G (kcal/mol) against PfUCHL3 (PDB ID:2WE6)	△G (kcal/mol) against HsUCHL3 (PDB ID:1XD3)	Comparative △G (kcal/mol)	Predicted IC_50_ value against PfUCHL3 (μM)	Predicted IC_50_ values against HsUCHL3
MMV676603	− 7.23	0.18	− 7.41	4.99	N.D
MMV688704	− 6.15	− 1.6	4.55	30.91	67.09 mM

N.D—not determined because the binding energy of MMV676603 was in positive range i.e., 0.18, hence, the software was not able to predict its IC_50_ value.

### Molecular insights: unravelling interactions between identified leads and the enzyme

**Figure 5:**
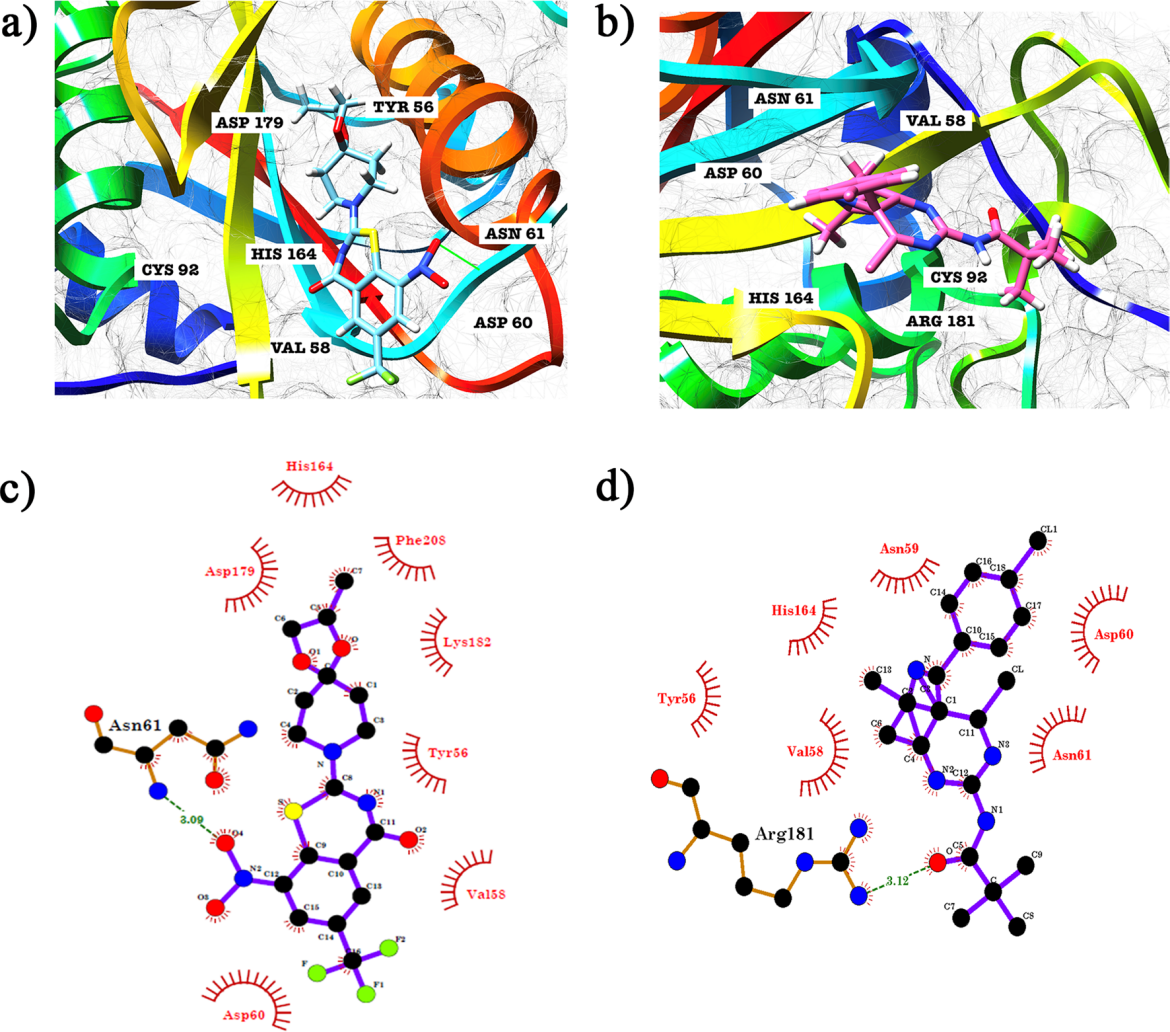
The two lead molecules docked to the active site cleft of PfUCHL3 (PDB ID: 2WE6): cartoon representation of predicted binding of (**a**) MMV676603 and (**b**) MMV688704 with PfUCHL3. 2-D ligand interaction maps of (**c**) MMV676603 and (**d**) MMV688704. Green lines depict hydrogen bonds and eye shaped residues exhibit hydrophobic interactions.

**Figure 6:**
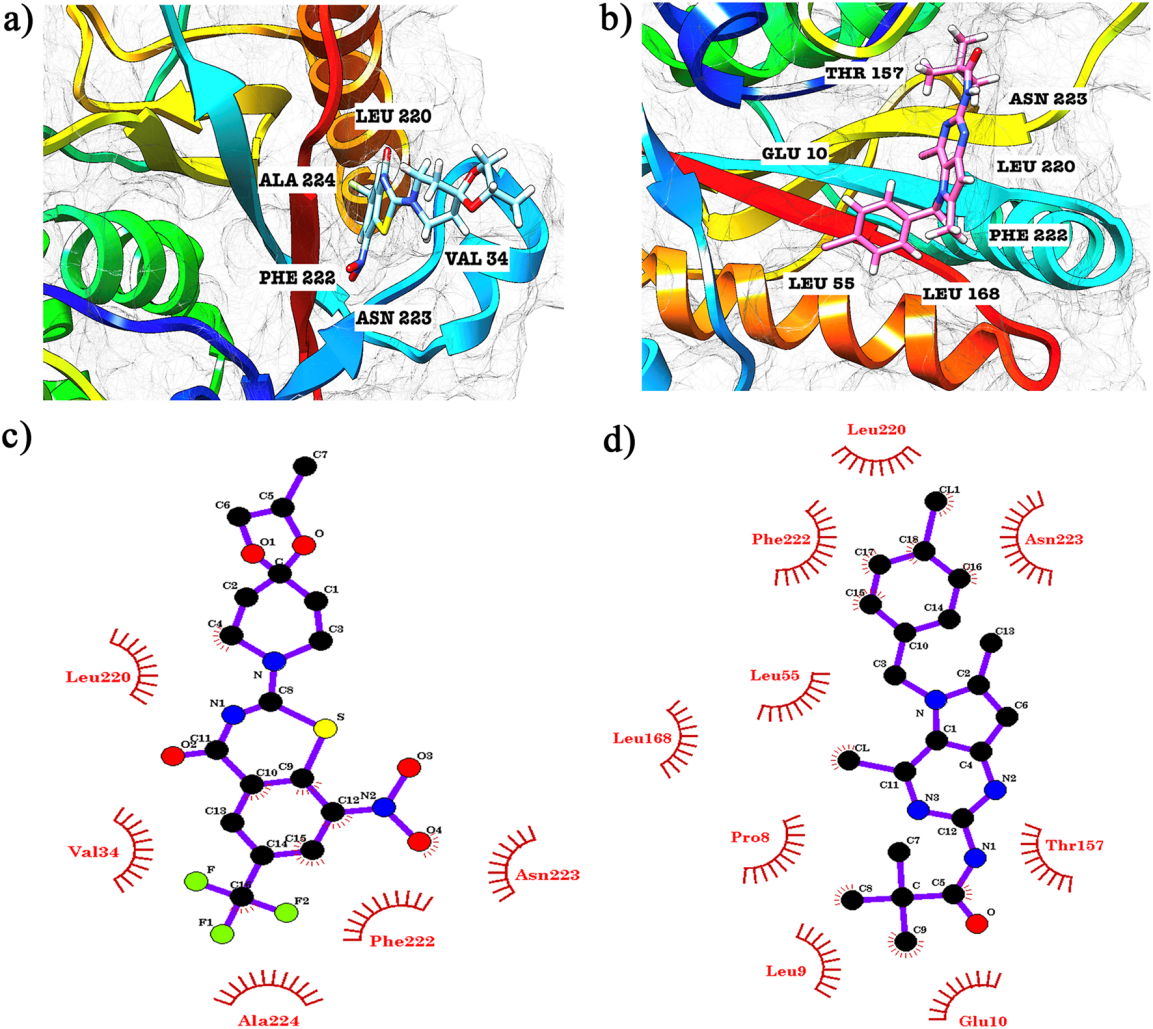
Leads docked to HsUCHL3 (PDB ID: 1XD3): cartoon representation of predicted binding of (**a**) MMV676603 and (**b**) MMV688704 with HsUCHL3. 2-D ligand interaction maps of (**c**) MMV676603 and (**d**) MMV688704. Green lines depict hydrogen bonds and eye shaped residues exhibit hydrophobic interactions.

Protein-inhibitor interaction studies were performed to gain better insight into the binding mode of the compunds MMV676603 and MMV688704. As depicted in Fig. 5a, MMV676603 obstructs the catalytic center of PfUCHL3 by funneling into the binding groove of PfUCHL3. This complex is stabilized by the formation of a hydrogen bond with ASN61 and hydrophobic interactions with HIS164, PHE208, ASP179, LYS182, TYR56, VAL58 and ASP60 of PfUCHL3 (Fig. 5c). Similarly, MMV688704 aligns in the binding groove of PfUCHL3 and overpasses the catalytic center (Fig. 5b) by forming a hydrogen bond with ARG181 and hydrophobic interactions with ASN59, HIS164, ASP60, TYR56, VAL58 and ASN61 (Fig. 5d). On the contrary, both MMV676603 and MMV688704 sit in an entirely different groove (Fig. 6a,b) and show interactions with residues that do not play any interphase in enzymatic activity (Fig. 6c,d) and do not demonstrate any specific binding to human ortholog, supported by docking results. Hence, these results indicate the selective binding of the identified hits to PfUCHL3 over HsUCHL3 and provide scope for further refinement and lead optimization studies.

### Anti-malarial effect of MMV676603 and MMV688704 on intra-erythrocytic stage of *P. falciparum*

**Figure 7:**
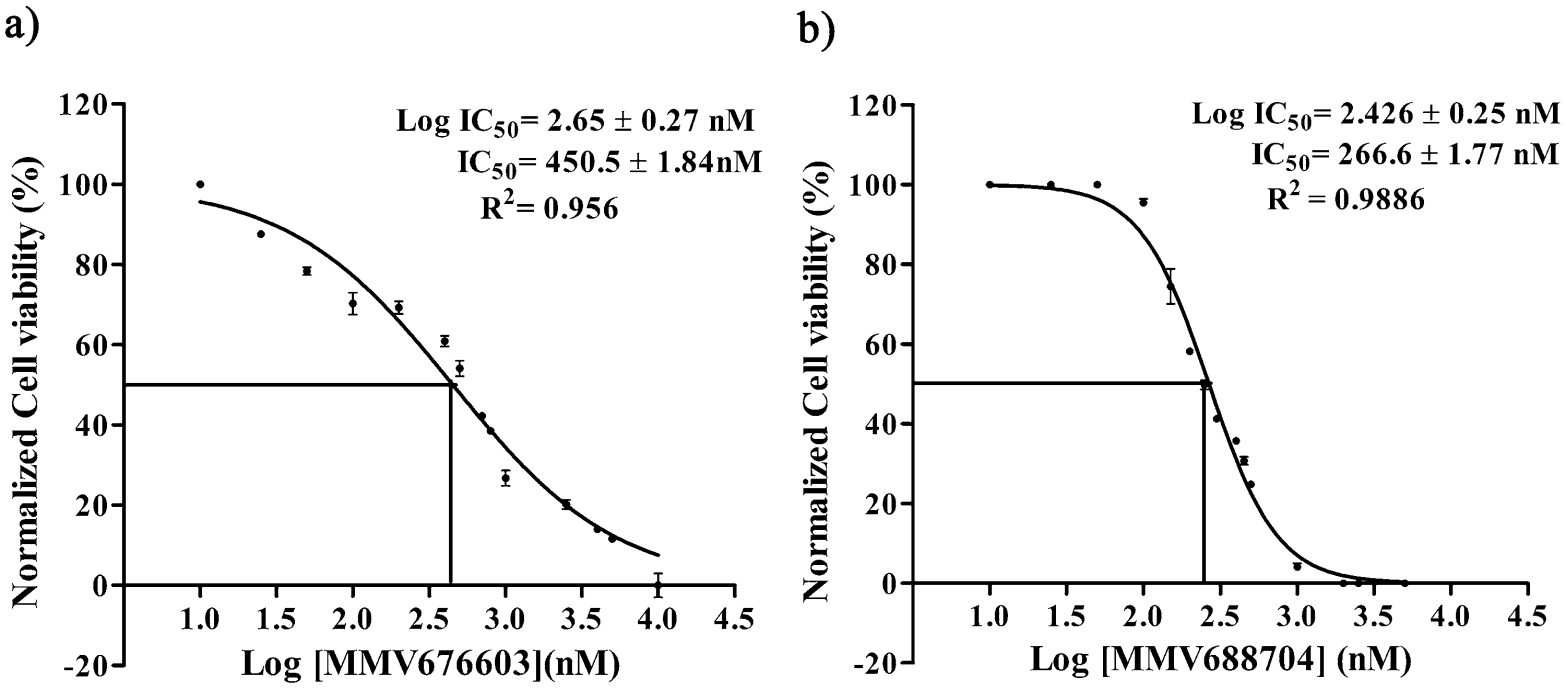
IC_50_ determination of MMV676603 and MMV688704 from Pathogen Box against blood stage of *P. falciparum* 3D7: dose–response curve indicating the Log IC_50_, IC_50_ and R^2^ values of (**a**) MMV676603 and (**b**) MMV688704 against asexual stages of *P. falciparum* 3D7. Inhibition at each data point is depicted as Mean ± S.D. of three independent experiments.

Since PfUCHL3 is cardinal for the parasite, therefore, we hypothesized that the inhibitors of PfUCHL3 would be effective in inhibiting parasite growth. To test this, we determined the inhibitory effect of the identified hits against the asexual intra-erythrocytic stage of *P. falciparum 3D7* in culture using SYBR Green I assay. The standard anti-malarial, Chloroquine was used as an internal reference for validation of the assay. As expected, both the compounds were equally effective and caused complete inhibition of parasite growth at a concentration of 10 μM. Compounds MMV676603 and MMV688704 displayed a dose dependent inhibition of Chloroquine (CQ)-sensitive *P. falciparum 3D7* strain with IC_50_ values 450.5 ± 1.84 nM and 266.6 ± 1.77 nM, respectively (Fig. 7a,b).

### In-vitro phenotypic and speed of action analysis of the two hit molecules

**Figure 8:**
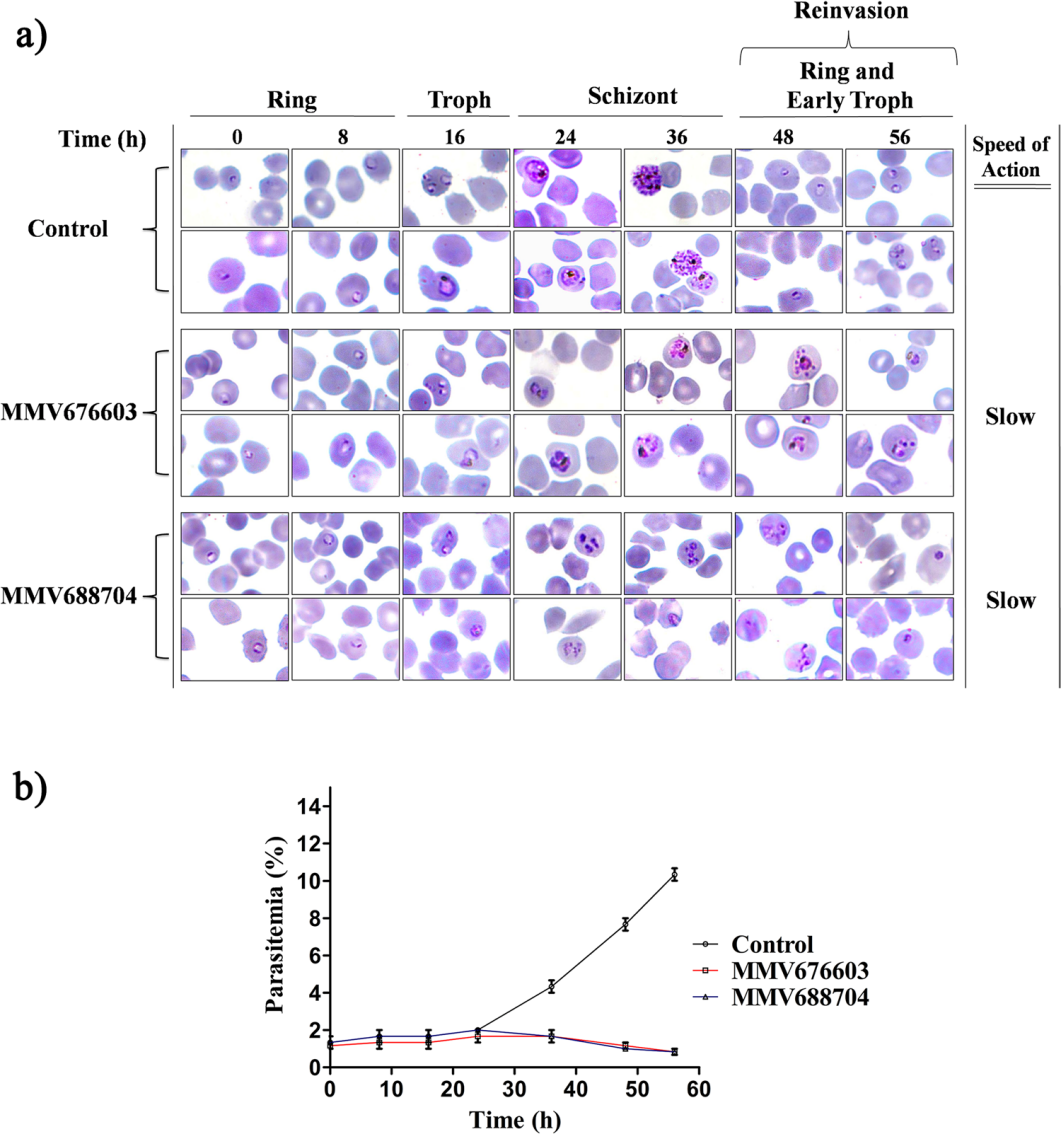
Effect of MMV676603 and MMV688704 on the growth and morphology of intraerythrocytic stage of *P. falciparum*, and assessment of their speed of action: synchronized *P. falciparum* 3D7 culture was treated with leads (MMV676603 and MMV688704) and their effect on parasite phenotype and growth was analyzed by (**a**) Giemsa stained blood smear micrographs at 0, 8, 16, 24, 36, 48 and 56 h (two representative images of each set). The speed of action of both the compounds (MMV676603 and MMV688704) was evaluated for one complete maturation cycle [starting from ring (0 h), trophozoite (16–24 h) to schizont (36 h)]. Both the compounds were considered as slow inhibitors as they hinder the parasite development at late stages (trophozoite and schizont). (**b**) The in-vitro reduction in parasitemia level was quantified and illustrated as a graph, indicating the parasitemia level as a function of parasite life cycle progression (time). Data are represented as Mean ± S.D.

To investigate the effect of MMV676603 and MMV688704 on parasite growth and phenotype, ring stage parasites were treated with the compounds at ten-fold concentration of the IC_50_ value and observed at various intervals for 56 h. Cultures treated with MMV676603 and MMV688704 displayed the appearance of young trophozoites at 16 h, exhibiting a similarity with the control set. However, both MMV676603 and MMV688704 treated parasites were arrested at the late trophozoite stage with shrunken, less granular and condensed appearance of the parasite. In comparison to control, both MMV676603 and MMV688704 treatment caused substantial delay in the transition from trophozoite to schizont stage. This results in complete arrest of the parasite growth at later stages of the parasite life cycle. Moreover, both MMV676603 and MMV688704 impeded erythrocyte reinvasion as indicated by absence of the new ring-stage parasite even at 56 h post drug incubation as shown in Fig. 8a. These results were further supported by quantification of parasitemia level at different time points, which revealed a continuous reduction in parasite count after 24 h accounting for almost ten-fold drop in parasitemia at 56 h (Fig. 8b). These results suggest that both MMV676603 and MMV688704 are slow acting inhibitors of parasite growth in culture (Fig. 8a).

### Effect of MMV676603 and MMV688704 on ubiquitination levels

**Figure 9:**
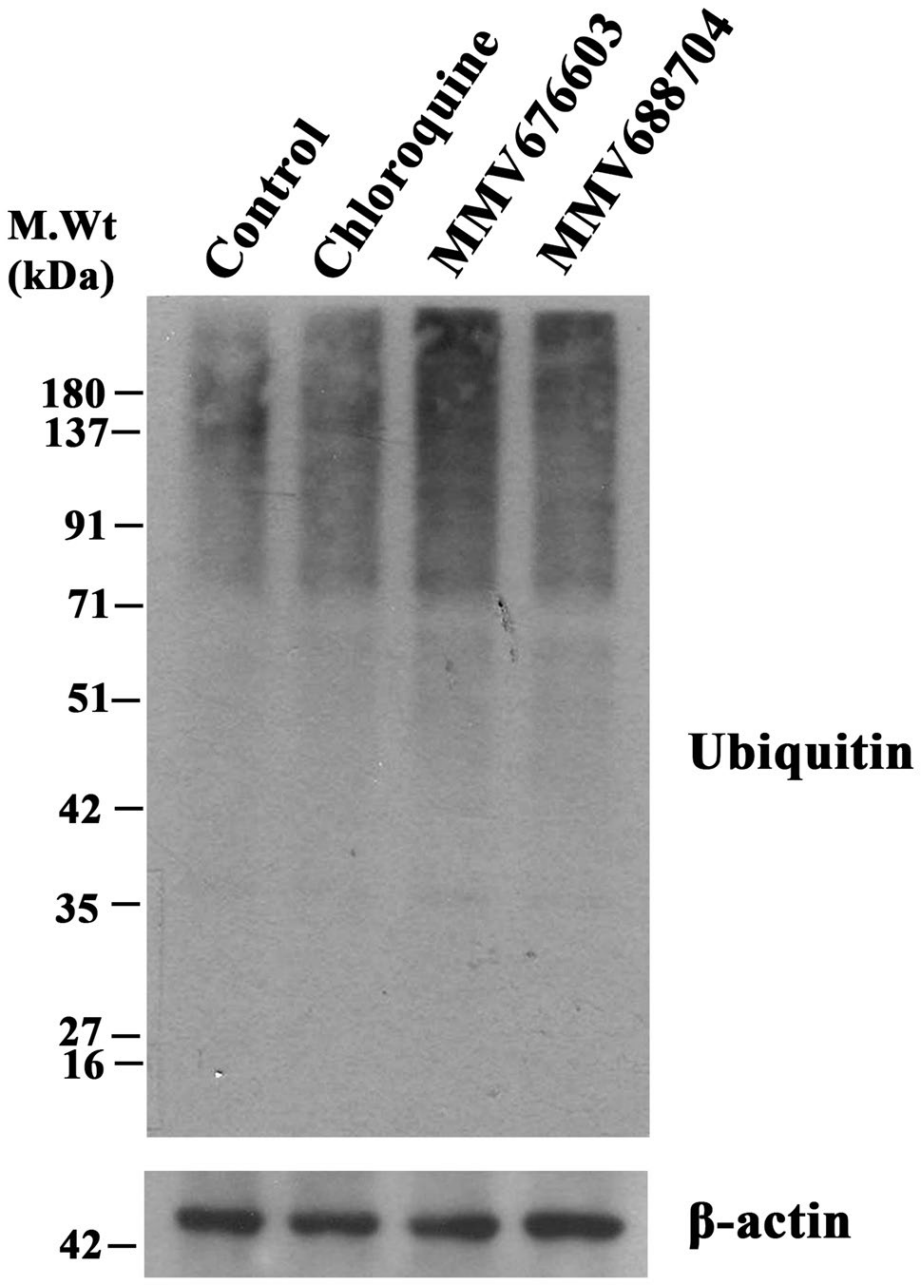
Effect of leads on ubiquitination levels: asynchronous *P. falciparum* culture was treated with vehicle control (Lane 1), Chloroquine (Lane 2) and identified compounds (MMV676603 (Lane 3), MMV688704 (Lane 4)). Following treatment, parasites were lysed and equal amount of parasite lysate was subjected to SDSPAGE and immunoblotting was carried out with ubiquitin antibody for ubiquitination levels. β-actin was kept as loading control. The experiment was performed atleast thrice with a respresentative image shown as figure. Full blots are provided in Supplementary Fig. S2.

Considering the in-vitro inhibitory action of the two molecules against PfUCHL3 activity, we determined their effect on parasite ubiquitination level to ascertain that the parasiticidal activity of the identified compounds is mediated through PfUCHL3 inhibition. With this aim, asynchronous parasite culture was exposed to three times the IC_50_ concentration of the two inhibitors and ubiquitination levels were detected post treatment. Treatment with both MMV676603 and MMV688704 induced a significant accumulation of high-molecular weight ubiquitin conjugates. In comparison to the vehicle control set, treatment with MMV676603 displayed the highest levels of ubiquitination laddering, followed by MMV688704 (Fig. 9). In contrast, Chloroquine, a known antimalarial exhibited negligible effect on the parasite ubiquitination levels (Fig. 9), indicating that these compounds alter PfUCHL3 activity for effective parasite killing.

### Cytotoxicity against mammalian cell lines

To demonstrate that the anti-parasitic effect of MMV676603 and MMV688704 was not due to general toxicity, the compound mediated cytotoxic effect was determined in the human hepatic cell line (HepG2) and human embryonic kidney cells (HEK-293T) with the help of MTT assay. No cytotoxicity was observed up to a concentration of 20 μM (100 times the effective concentration) (Fig. S3). Furthermore, as summarized in Table 2, compound MMV676603 and MMV688704 displayed a Selectivity Index of 50 and 100, respectively, presumably indicating a high anti-parasitic selective profile of these hits.

**Table 2:** In-vitro activity of the two hits identified from Pathogen Box against parasite and mammalian cell culture.

Compound no.	Compound ID	*P. falciparum* 3D7 IC_50_ (nM)	HEK-293T IC_50_ (μM)	HepG2 IC_50_ (μM)	Selective Index (HEK-293T)	Selective Index (HepG2)
1	**MMV676603**	450.5 ± 1.84	> 20	> 20	50	50
2	**MMV688704**	266.6 ± 1.77	> 20	> 20	100	100

## Discussion

Regardless of the extensive efforts to combat this infectious disease, malaria continues to torment the world with its severity and complexity^69^. Limited protection by licensed vaccine and development of accelerated resistance to current clinical anti-malarials has overcome the efforts to contain this disease^70,71^. Such a scenario, stimulates an urgent need for new drug discovery programs to mitigate associated problems and develop new and cost effective anti-malarial therapies with least side effects.

DUBs are a group of highly sophisticated enzymes known to regulate a number of cellular functions and their deregulation contribute to the development of severe clinical manifestations^28,72^. Likewise, compelling evidences from literature highlight the critical importance of DUBs in parasite biology. However, till recent times, studies exploiting DUBs as a therapeutic target in *Plasmodium* are limited. PfUCHL3 is one such promising member of *Plasmodium* deubiquitinase family, whose absence exhibits a lethal effect on parasite survival, thereby, validating its utmost importance as a therapeutic target^46^. A report by Franco et al., identified inhibitors against PfUCHL3 from the ZINC database by virtual screening^73^. Unfortunately, the study lacked enzymatic validation and biological assay of the identified hits. Even though, PfUCHL3 shares some identity with HsUCHL3, the subtle differences in the active site pocket and substrate binding groove could serve as a fundamental scaffold for the development of selective therapeutics^55^. Therefore, in the present study, we focused to query Pathogen Box compounds for identification of novel PfUCHL3 inhibitors as potent anti-malarials.

Herein, we performed targeted docking in search of selective inhibitors of PfUCHL3 over HsUCHL3 using AutoDock suite. The molecules, exhibiting selectivity towards *P. falciparum* UCHL3 and displaying drug like properties were evaluated for their in vitro enzyme inhibition assay at 100 μM. Interestingly, MMV676603 and MMV688704 were identified as potent inhibitors that potentially impeded PfUCHL3 activity at sub-micromolar concentration, which was in close association with the predicted in-silico IC_50_ values and was further supported by predicted dock score values. We also performed a detailed analysis of the docked complexes to gain better insight into the binding mechanism of both the hits. Notably, MMV676603 blocks the binding pocket of PfUCHL3 by residing in the binding groove with its azapiro ring facing the catalytic unit (CYS92, HIS164 and ASP179) whereas the benzo ring faces the crossover loop. Such affirmative orientation positions the hydrogen acceptor and donors in the close vicinity thus contributing to the formation of strong non-covalent interactions, including hydrogen bonds with the catalytic triad along with ASP60, ASN61, van der waal interactions with PHE208 and TYR56 and ionic interactions with LYS182. The presence of three fluorines in MMV676603 further strengthens these interactions, thereby, enhancing the selectivity and permeability. Incidentally, fluorination of ligand molecules is a common practice to elevate the potency of the inhibitors. Whilst, these are short interactions, yet they impart a significant effect on protein ligand binding^74^. On the contrary, MMV676603 lacks proper orientation in the binding groove and interacts with the amino acid residues (LEU220-ALA224) lining the C-terminal region of HsUCHL3. Together, these collated evidences advocate for more proficient suppressive effects of MMV676603 on PfUCHL3 activity as compared to its mammalian ortholog. Additionally, MMV688704 also aligns itself in the binding groove of PfUCHL3 with the tail of the benzene ring facing the crossover loop and the pyrimidine ring aligns towards the catalytic center of PfUCHL3. This allows MMV688704 to form a strong network of non-covalent interactions with the amino acid residues surrounding the binding pocket. In contrast, the horizontal orientation of the MMV688704 (facing the C-terminal region) in HsUCHL3 hampers its interaction with the catalytic groove, thereby, impeding its potency towards human UCHL3. Both the compounds obey all druglikeliness parameters as depicted by bioavailability radar and even exhibit zero violation of Lipinski rule of five, which makes them suitable for oral absorption.

The compounds MMV676603 and MMV688704 were evaluated for their anti-malarial potential and compounds MMV676603 and MMV688704 were highly efficient against *P. falciparum* 3D7 (CQ-sensitive strain) in culture with an IC_50_ value of 450.5 ± 1.84 nM and 266.6 ± 1.77 nM, respectively. Both MMV676603 and MMV688704 displayed asexual stage dependent growth inhibition after prolonged exposure of drug (after 24 h) and caused phenotypic and developmental aberrations in parasites. The treated parasite was completely arrested at late stages (trophozoites/schizont) with a compact and shrunken appearance as evidenced by lack of ring stage even after 56 h. The trophozoite stage marks the initiation of a highly replicative phase in the parasite life cycle, which indicates high dependency on the ubiquitin regulatory machinery for invasion and differentiation^16^. Therefore, it can be envisaged that DUBs like PfUCHL3 might regulate the critical processes in late asexual stages of the parasite and its inhibition by MMV676603 and MMV688704 hampers the parasite growth by altering the protein homeostasis and other essential pathways. Additionally, to affirm whether the biological action of these compounds is exerted through PfUCHL3 inhibition, the parasite ubiquitination levels were determined upon exposure to the selected hits. As compared to control and CQ, a known antimalarial with different mode of action, both MMV676603 and MMV688704 treated parasites exhibited a marked increase in the ubiquitination levels, thereby, suggesting their inhibitory role towards the deubiquination process. In comparison to MMV676603, a less intense ubiquitination pattern in MMV688703 treated set could be an attribute of differential impact on PfUCHL3 activity, rendering its dexterity to curb the deubiquitination event in the parasite milieu. Despite such variable biological effects, the enhanced ubiquitination laddering in compounds treated sets provide compelling evidences for PfUCHL3-mediated parasite killing by the identified hits. This, coupled with other results, make it very likely that the compounds MMV676603 and MMV688704 might be equally effective against the resistant strains of the parasite and possibly be used as an adjunct therapeutics in ACTs., Pathogen Box classifies MMV676603 and MMV688704 against tuberculosis and toxoplasmosis, respectively. However, this is the first report of their identification as an anti-malarial. MMV676603 belongs to the nitrobenzothiazinones family and is effective against the clinical isolates of tuberculosis^75^. Currently, it is under pre-clinical trials for the treatment of drug-resistant tuberculosis^76^. Some studies have also identified its potential against *N. brasiliensi*^77^ *a*nd Corynebacterineae^78^. Alongside, this drug acts by forming a covalent bond with CYS387 of DprE1of an oxidoreductase enzyme that is vital for cell wall arabinan synthesis and ultimately death^78,79^. Consequently, we can infer that MMV676603 inhibits PfUCHL3 activity by altering the key interaction of cysteine residue present in the active site pocket of the enzyme. Such inhibition renders the enzyme non-functional by obstructing the overall parasite growth. In contrast, MMV688704 belongs to the family of pyrimidine, mainly known as DHFR inhibitors^80^ with minimal evidence of it as anti-toxoplasmosis. In our study, MMV688704 displayed a better efficacy against the parasites in culture with enzyme inhibition at the micro molar range, which could be a result of an additional mode of action. Hence, the results necessitate further lead optimization and chemical engineering to improve its efficacy. These studies were further complemented by cytotoxicity assay on mammalian cell lines. Expectedly, compound MMV676603 and MMV688704 selectively inhibited parasites over the mammalian cell lines (HEK-293T and HepG2 cells) with S.I value of more than 50, which was in corroboration with our in-silico studies. However, in-vivo and dynamic mechanistic studies are warranted to provide a better insight into the potential of PfUCHL3 as a therapeutic target. Concurrently, our study identified MMV676603 and MMV688704 as promising antimalarial with appreciable inhibition of PfUCHL3 activity.

## Conclusion

The present study represents first comprehensive attempt to repurpose the collection of MMV Pathogen Box compounds targeting UCHL3 of *P. falciparum* for the development of anti-malarial chemotherapy. In this study, we identified two novel promising non-cytotoxic small molecules, MMV676603 and MMV688704, as PfUCHL3 antagonists with biological activity against *P. falciparum* as an outcome of a stringent screening. In comparison to MMV676603, MMV688704 was found to be more potent against the parasite, but less effective against recombinant PfUCHL3. Therefore, it is tempting to dissect its probable mode of action. Besides this, both MMV676603 and MMV688704 hold the potential as potent anti-malarial. Therefore, improvement towards selectivity for PfUCHL3 over human ortholog and in-vivo studies will complement these findings. The outcome of this study provides a scaffold for future optimization of more specific, effective and selective compounds with its implication as a therapeutic as well as a tool to identify the biological functions of the enzyme. Overall, inhibitors targeting the UCHL3 family of DUBs can enlighten the drug development path and discovery against a range of parasitic and infectious diseases.

## Material and methods

### Pathogen Box compounds

The Pathogen Box was procured from Medicine for Malaria Venture (MMV) foundation (Geneva, Switzerland). The library of compounds was supplied in 96-well microtitre plate at a concentration of 10 mM in dimethyl sulfoxide (DMSO) (10 μL) and further dilutions were prepared according to MMV instructions. The details about plate layout, chemical formula and other biological activity are available online as Pathogen Box supporting information in a form of an Excel spreadsheet (https://www.pathogenbox.org/about-pathogen-box/supporting-information).

### Constructs, antibodies and chemicals

Bacterial expression vector encoding PfUCHL3 was a generous gift from Dr. Hidde Ploegh, Boston Children’s Hospital, MA and Katerina Artavanis-Tsakonas, Department of Pathology, University of Cambridge, Cambridge, UK. The gene encoding HsUCHL3 was amplified from HEK-293T cDNA using 5′-GGT CAT ATG GAG GGT CAA CGC TGG CTG-3′ and 5′-CCC AAG CTT CTA TGC TGC AGA AAG AGC-3′ as forward and reverse primer, respectively. The amplified gene was cloned between NdeI and HindIII of pET28a(+) as a N-terminus 6 X His tag. The gene sequences were verified by DNA sequencing. Anti-ubiquitin and anti-β-actin antibodies were purchased from Santa Cruz Biotechnology Inc. (Santa Cruz, CA, USA).  Isopropyl β-D-1-thiogalactopyranoside (IPTG) was obtained from Bio Basic Inc. (Markham, Canada). SYBR Green I, penicillin and nickel nitrilotriacetic acid (Ni–NTA) beads were procured from Thermo Scientific (Rockford IL, USA). Dithiothreitol (DTT) and hypoxanthine were obtained from Sigma (St. Louis, MO, USA). Powdered RPMI-1640 and Albumax II were obtained from GIBCO (ThermoFisher Scientific, Waltham, MA, USA). DMEM and streptomycin were purchased from Invitrogen (Carlsbad, CA, USA). All other reagents were of analytical grade.

### Heterologous expression and purification of recombinant proteins

Expression vector encoding PfUCHL3 and HsUCHL3 was transformed in *E. coli BL21(DE3)* cells*.* Protein expression and purification of the recombinant proteins were performed as described previously with slight modification^46^. In brief, protein expression was induced with 1 mM IPTG for 16 h at 30 °C. The cells were harvested and the pellet was lysed with lysis buffer (50 mM Tris, 150 mM NaCl, 2 mM DTT) containing protease inhibitor cocktail, lysozyme and DNase. The lysate was sonicated and the clear lysate was loaded onto a pre-equilibrated Ni–NTA column and protein was eluted with increasing concentration of imidazole hydrochloride. Subsequently, the eluants were subjected to size exclusion chromatography (AKTA Prime, GE Healthcare, Björkgatan, Uppsala, Sweden) and the fractions were collected and purity of the protein was determined by SDS-PAGE analysis.

### Structure based virtual screening (SBVS)

The dataset comprising of 400 molecules from MMV, Geneva were docked against the active site of PfUCHL3 and its human ortholog whose crystal structure was retrieved from Protein Data Bank (PDB ID: 2WE6 and 1XD3 respectively). Autodock Racoon software^81^ was used for docking. Before commencing for docking, the protein and ligands were prepared separately. Water molecules and cofactors were removed from the protein. However, the SMILES of 400 compounds were used to retrieve the 2D structures from ChEMBL database, followed by their conversion to PDB via Pymol and lastly to the PDBQT format. Subsequently, the grid was generated by keeping the active site residues (CYS92, HIS164 and ASP179) of PfUCHL3 as highlights, which are dissimilar in positioning when compared with human UCHL3 whose binding pocket comprises of CYS95, HIS169 and ASP184. Top 100 compounds having a comparative binding energy value of ≥ 3 kcal/mol were selected for ADME analysis.

### ADME screening

The assortment of flawed molecular entities in the early phases of drug discovery diminishes the error rate and increases the efficacy in the initial stage. Hence, the pharmacokinetic activities of the leads were estimated using SWISS ADME^82^. Only drug-like candidates with zero violation of Lipinski rule of five and with easy absorption, distribution, metabolism and excretion were taken forward.

### In-vitro DUB assay

The selected compounds were initially screened against recombinant PfUCHL3 at a concentration of 100 μM using fluorimetric assay as described by Artavanis et al.^46^ with relevant modification. In brief, the assay was performed in a 60 μl reaction volume containing 10 pM of enzyme in reaction buffer (50 mM Tris–Cl, pH-8.0, 150 mM NaCl, 2 mM DTT, 2 mM EDTA and 0.1 mg/mL BSA) and 100 μM of compounds. The fluorogenic peptide substrate Ub-AMC (Ubiquitin C-terminal tagged 7-amido-4-methylcoumarin)(Boston Biochem,Cambridge, MA, USA) was added at a final concentration of 125 nM and the release of AMC was continuously monitored (Excitation: 485 nm and Emission: 535 nm) for a period of 30 min at 25 °C. The enzyme inhibition assay for each compound was performed in triplicates. The compounds inhibiting ≥ 50% of PfUCHL3 activity were identified as potent hits. The IC_50_ of best compounds was determined through the dose–response workspace of GraphPad Prism Software (GraphPad Co. Ltd., San Diego, CA, USA).

### In-vitro culturing and synchronization of parasite

*Plasmodium falciparum 3D7* was cultured with human O^+^ human erythrocyte (5% hematocrit) in RPMI-1640 (Gibco, ThermoFisher Scientific, Waltham, MA, USA) medium supplemented with 0.5% (w/v) Albumax (Gibco, ThermoFisher Scientific, Waltham, MA, USA), Hypoxanthine (50 mg/L), Gentamicin (10 mg/L) and Ampicillin (10 mg/L) in mixed gas environment (5% O_2_, 5% CO_2_ and 90% N_2_) at 37 °C^83^. Human whole blood was procured from the Rotary Blood Bank, New Delhi. Under sterile conditions, erythrocytes were obtained by removal of plasma and peripheral blood mononuclear cells (PBMCs) using histopaque gradient. Parasitemia levels were routinely assessed using Giemsa staining of blood smears. Synchronization was performed using the sorbitol lysis method^84^. The parasite culture was treated with 5% D-sorbitol for 10 min at 37 °C for the enrichment of ring stage parasite. The culture was pelleted by centrifugation and washed thrice with media. In addition, the high synchrony level of the parasite was maintained by performing two consecutive sorbitol treatments at an interval of 4 h. The parasite was visualized by microscopic analysis of Giemsa stained blood smears.

### Drug susceptibility assay for asexual stages of *P. falciparum*

In-vitro anti-malarial efficacy of compounds was determined by SYBR-Green I based proliferation assay as reported previously^85^ with slight modification. In brief, various dilutions of test compounds (10 μL) were added to 96-well microdilution plates and asynchronous parasite culture at 4% hematocrit and 0.5% parasitemia was added to pre-dosed plates in a total volume of 100 μL. After 48 h of incubation at 37 °C under 90% N_2_, 5% CO_2_ and 5% O_2_, the plates were frozen at − 20 °C for an additional 24 h. Subsequently, plates were thawed and 100 μL of lysis buffer (20 mM Tris base, 5 mM EDTA, 0.0008% (v/v) Triton X-100, 0.008% (m/v) saponin, pH 8.0) containing 0.002% (v/v) SYBR Green I was added. Plates were incubated at 37 °C for 1 h and fluorescence readings were taken using Infinite M 200 PRO microplate reader (TECAN, Männedorf, Switzerland) with excitation and emission wavelengths of 485 nm and 535 nm, respectively. The half maximal inhibitory concentration (IC_50_) was determined via GraphPad PRISM 5.0 using non-linear regression analysis.

### Phenotypic evaluation and speed of action studies

Synchronized culture containing ring stage parasites at 1% parasitemia and 2% hematocrit was treated with test compounds at tenfold the IC_50_ concentration. After addition of compounds, the parasitemia level was quantified and morphological changes were studied by microscopic analysis of Giemsa stained thin blood smear at 0, 8, 16, 24, 36, 48 and 56 h. The speed of action of the compounds was determined on the basis of their effect on the early or late stage of the erythrocytic stages of the parasite as described by Terquille et al.^86^.

### Effect of selected hits on parasite ubiquitination levels

Asynchronus parasite cultures at 5% hematocrit and 4–5% parasitemia were incubated in the absence (vehicle control) or presence of three times the IC_50_ concentration of the identified compounds or Chloroquine (CQ) for 24 h at 37 °C. Post treatment, cultures were harvested and pellets were extracted with 0.1% (w/v) saponin for 15 min on ice. Cells were pelleted and parasite pellets were washed with PBS until the supernatant became clear. Parasite pellets were resuspended in RIPA buffer (150 mM NaCl, 1% Nonidet P-40, 0.5% sodium deoxycholate, 0.1% SDS, 50 mM Tris pH 8.0) and lysed on ice for 45 min, followed by mild 6 cycles of sonication (2 s On and 2 s Off) and the clear lysate was obtained post centrifugation at 13,800×*g* for 15 min at 4 °C. Protein concentration was estimated by Bradford and an equal amount of parasite lysates were resolved on SDS-PAGE and subjected to immunodetection using anti-ubiquitin antibody and anti β-actin antibody (loading control).

### Cytotoxicity assessment against mammalian cell lines

HepG2 (human hepatocellular carcinoma) and HEK-293T (human embryonic kidney) cells were cultured DMEM (Invitrogen, Carlsbad, CA) medium supplemented with 10% fetal bovine serum and antibiotics (100 U/mL of penicillin and 100 mg/mL streptomycin) in a humidified 5% CO_2_ chamber at 37 °C.The cytotoxicity of top hits was assessed against HepG2 and HEK-293T cells using MTT assay as described previously^87^. In brief, cells were seeded in a 96 well microplate and incubated for 24 h at 37 °C in a CO_2_ incubator. After 24 h, cells were exposed to different dilutions of drugs (prepared in respective medium) for 24 h. Post incubation, MTT was added and the plate was incubated at 37 °C for 4 h. Subsequently, the supernatant was removed and DMSO was added to solubilize the formazan crystals. Absorbance was measured at 570 nm using an Infinite M 200 PRO microplate reader (Tecan, Switzerland) with the Magellan 7 software. IC_50_ values were determined by nonlinear regression analysis GraphPad Prism 5 software (GraphPad Software, San Diego, CA).

### Selective Index (S. I.)

Selective Index of the top hits was determined by application of the following formula^62^:

$${\text{S}}.{\text{I}}_{P.falciparum} = {\text{IC}}_{{{5}0({\text{HEK}} - {\text{293T or HepG2}})}} /{\text{IC}}_{{{5}0(P.falciparum)}}$$

### Statistical analysis

Preparation of graphs and statistical analysis of data were performed using Prism 5 software (GraphPad Software, San Diego, CA). A non-linear regression sigmoidal dose dependent curve fit was applied to dose–response data for the determination of half maximal concentrations (IC_50_s). All data are presented as Mean ± S.D. from atleast three independent experiments.

### Ethical declaration

Parasite culture was maintained as per safety guidelines of the Department of Biotechnology, Ministry of Science and Technology, Government of India and has been approved by the Institutional Biosafety Committee of the University of Delhi, South Campus, New Delhi (Ref. no.-155/AN/Biochem/UDSC/IBSC/02/08/2019).

## Supplementary Information

Supplementary Information.

## Abbreviations

PfUCHL3: *Plasmodium falciparum* ubiquitin C-terminal hydrolase 3
DUB: Deubiquitinase
MMV: Medicine for malaria venture
MTT: 3-(4,5-Dimethylthiazol-2-yl)-2,5-diphenyl tetrazolium bromide
HepG2: Human hepatocellular carcinoma
HEK-293T: Human embryonic kidney
Ub-AMC: Ubiquitin C-terminal 7-amido-4-methylcoumarin
IPTG: Isopropyl β-D-1-thiogalactopyranoside
PTM: Post translational modifications
IC_50_: Half maximal inhibitory concentration
S.I.: Selective index
