# Supplementary material for: Repurposing the Pathogen Box compounds for identification of potent anti-malarials against blood stages of Plasmodium falciparum with PfUCHL3 inhibitory activity
Source: Sci Rep. 2022 Jan 18;12:918. doi: 10.1038/s41598-021-04619-4 (PMC8766476; doi:10.1038/s41598-021-04619-4)
Supplement: Supplementary file 1 — Supplementary Information. [file 41598_2021_4619_MOESM1_ESM.docx]

**Repurposing the Pathogen Box compounds for identification of potent anti-malarials against blood stages of *Plasmodium falciparum* with PfUCHL3 inhibitory activity.**

**Hina Bharti­ ^1^, Aakriti Singal ^1^, Manisha Saini ^1^, Pradeep Singh Cheema ^1^, Mohsin Raza ^1^ , Suman Kundu ^1^ and Alo Nag ^1^***

^1^Department of Biochemistry, University of Delhi South Campus, Benito Juarez Road, New

Delhi-110021, India.

Phone: +91-11-24117363, 24112503, Fax: 91-11-24115270.

* Corresponding author (Email id: anag@south.du.ac.in)

***Supplementary Tables***

***SupplementaryTable S1: Table depicting binding energies of all 400 Pathogen Box compounds against PfUCHL3 (2WE6) and HsUCHL3 (1XD3).*** *Top 100 compounds with comparative score greater than -3kcal/mol (cells colored in blue) were taken for further assessment.*

| **S.No.** | **Compound** | **△G (kcal/mol) against PfUCHL3 (PDB ID:2WE6)** | **△G (kcal/mol) against HsUCHL3 (PDB ID:1XD3)** | **Comparative △G (kcal/mol)** |
| --- | --- | --- | --- | --- |
|  | **MMV688508** | -3.57 | 5.86 | -9.43 |
|  | **MMV687700** | -7.37 | 1.83 | -9.2 |
|  | **MMV022029** | -7.24 | 1.75 | -8.99 |
|  | **MMV688362** | -4.85 | 3.92 | -8.77 |
|  | **MMV688990** | -3.83 | 4.55 | -8.38 |
|  | **MMV688509** | -7.2 | 1.05 | -8.25 |
|  | **MMV024937** | -5.91 | 2.31 | -8.22 |
|  | **MMV020512** | -4.41 | 3.6 | -8.01 |
|  | **MMV676380** | -6.15 | 1.79 | -7.94 |
|  | **MMV687798** | -5.95 | 1.92 | -7.87 |
|  | **MMV085499** | -5.21 | 2.54 | -7.75 |
|  | **MMV688552** | -5.4 | 2.1 | -7.5 |
|  | **MMV676603** | -7.23 | 0.18 | -7.41 |
|  | **MMV688762** | -4.72 | 2.5 | -7.22 |
|  | **MMV688180** | -5.46 | 1.7 | -7.16 |
|  | **MMV688407** | -5.33 | 1.83 | -7.16 |
|  | **MMV687813** | -3.96 | 2.97 | -6.93 |
|  | **MMV688271** | -4.04 | 2.85 | -6.89 |
|  | **MMV688474** | -5.17 | 1.68 | -6.85 |
|  | **MMV001493** | -4.7 | 2.15 | -6.85 |
|  | **MMV689029** | -4.75 | 1.98 | -6.73 |
|  | **MMV023949** | -0.17 | 6.54 | -6.71 |
|  | **MMV020591** | -5.76 | 0.87 | -6.63 |
|  | **MMV007638** | -6.32 | 0.18 | -6.5 |
|  | **MMV676597** | -5.31 | 1.12 | -6.43 |
|  | **MMV007133** | -5.63 | 0.76 | -6.39 |
|  | **MMV688262** | -5.33 | 1.01 | -6.34 |
|  | **MMV676539** | -6.94 | -0.67 | -6.27 |
|  | **MMV676602** | -4.39 | 1.86 | -6.25 |
|  | **MMV020136** | -5.34 | 0.89 | -6.23 |
|  | **MMV676478** | -4.09 | 2.08 | -6.17 |
|  | **MMV634140** | -5.99 | 0.17 | -6.16 |
|  | **MMV687729** | -4.69 | 1.43 | -6.12 |
|  | **MMV687172** | -5.49 | 0.61 | -6.1 |
|  | **MMV688472** | -5.58 | 0.47 | -6.05 |
|  | **MMV688466** | -4.14 | 1.91 | -6.05 |
|  | **MMV688361** | -6.26 | -0.24 | -6.02 |
|  | **MMV024406** | -5.12 | 0.78 | -5.9 |
|  | **MMV007471** | -6.2 | -0.42 | -5.78 |
|  | **MMV024114** | -7.76 | -2.02 | -5.74 |
|  | **MMV020982** | -4.29 | 1.35 | -5.64 |
|  | **MMV688270** | -2.29 | 3.34 | -5.63 |
|  | **MMV022478** | -3.99 | 1.59 | -5.58 |
|  | **MMV023860** | -8.57 | -3 | -5.57 |
|  | **MMV688471** | -4.93 | 0.61 | -5.54 |
|  | **MMV688178** | -5.72 | -0.2 | -5.52 |
|  | **MMV026490** | -2.82 | 2.69 | -5.51 |
|  | **MMV023953** | -6.9 | -1.53 | -5.37 |
|  | **MMV020289** | -6.89 | -1.53 | -5.36 |
|  | **MMV024829** | -4.17 | 1.18 | -5.35 |
|  | **MMV1029203** | -5.15 | 0.15 | -5.3 |
|  | **MMV658993** | -6.77 | -1.48 | -5.29 |
|  | **MMV407539** | -3.29 | 1.95 | -5.24 |
|  | **MMV688844** | -3.52 | 1.64 | -5.16 |
|  | **MMV676509** | -5.11 | 0.03 | -5.14 |
|  | **MMV688364** | -3.86 | 1.28 | -5.14 |
|  | **MMV671636** | -2.6 | 2.53 | -5.13 |
|  | **MMV688283** | -3.65 | 1.43 | -5.08 |
|  | **MMV030734** | -4.72 | 0.33 | -5.05 |
|  | **MMV085071** | -6.97 | -2.06 | -4.91 |
|  | **MMV024397** | -5.11 | -0.35 | -4.76 |
|  | **MMV687239** | -6.45 | -1.76 | -4.69 |
|  | **MMV676476** | -5.59 | -0.91 | -4.68 |
|  | **MMV099637** | -4.02 | 0.64 | -4.66 |
|  | **MMV659004** | -5.54 | -0.92 | -4.62 |
|  | **MMV020165** | -4.46 | 0.16 | -4.62 |
|  | **MMV688704** | -6.15 | -1.6 | -4.55 |
|  | **MMV688991** | -5.53 | -1.01 | -4.52 |
|  | **MMV688179** | -3.93 | 0.57 | -4.5 |
|  | **MMV090930** | -4.89 | -0.4 | -4.49 |
|  | **MMV668727** | -3.8 | 0.67 | -4.47 |
|  | **MMV689061** | -4.8 | -0.46 | -4.34 |
|  | **MMV032967** | -4.57 | -0.24 | -4.33 |
|  | **MMV019742** | -4.03 | 0.24 | -4.27 |
|  | **MMV688845** | -3.53 | 0.61 | -4.14 |
|  | **MMV004168** | -4.17 | -0.05 | -4.12 |
|  | **MMV687246** | -2.43 | 1.65 | -4.08 |
|  | **MMV688327** | -3.68 | 0.3 | -3.98 |
|  | **MMV675997** | -3.28 | 0.69 | -3.97 |
|  | **MMV687189** | -4.88 | -1.05 | -3.83 |
|  | **MMV688550** | -2.33 | 1.49 | -3.82 |
|  | **MMV010576** | -6.03 | -2.23 | -3.8 |
|  | **MMV688796** | -5.87 | -2.15 | -3.72 |
|  | **MMV020623** | -2.04 | 1.47 | -3.51 |
|  | **MMV008439** | -4.37 | -0.89 | -3.48 |
|  | **MMV019189** | -4.09 | -0.65 | -3.44 |
|  | **MMV689028** | -3.28 | 0.14 | -3.42 |
|  | **MMV675998** | -5 | -1.63 | -3.37 |
|  | **MMV687775** | -4.67 | -1.3 | -3.37 |
|  | **MMV1198433** | -3.51 | -0.16 | -3.35 |
|  | **MMV019087** | -4.83 | -1.51 | -3.32 |
|  | **MMV000858** | -7.18 | -3.9 | -3.28 |
|  | **MMV676528** | -6.6 | -3.35 | -3.25 |
|  | **MMV688761** | -5.96 | -2.71 | -3.25 |
|  | **MMV560185** | -4.99 | -1.78 | -3.21 |
|  | **MMV687794** | -4.65 | -1.44 | -3.21 |
|  | **MMV688768** | -4.73 | -1.53 | -3.2 |
|  | **MMV688921** | -4.28 | -1.08 | -3.2 |
|  | **MMV688350** | -1.32 | 1.69 | -3.01 |
|  | **MMV676555** | -5.05 | -2.05 | -3 |
|  | **MMV024035** | -1.15 | 1.8 | -2.95 |
|  | **MMV019790** | -5.73 | -2.82 | -2.91 |
|  | **MMV676536** | -4.39 | -1.5 | -2.89 |
|  | **MMV676554** | -3.39 | -0.57 | -2.82 |
|  | **MMV687703** | -2.35 | 0.46 | -2.81 |
|  | **MMV023233** | -4.8 | -2 | -2.8 |
|  | **MMV688936** | -3.97 | -1.18 | -2.79 |
|  | **MMV006901** | -6.4 | -3.62 | -2.78 |
|  | **MMV688942** | -4.67 | -1.89 | -2.78 |
|  | **MMV687812** | -1.79 | 0.97 | -2.76 |
|  | **MMV690102** | -1.58 | 1.15 | -2.73 |
|  | **MMV676558** | -6.39 | -3.66 | -2.73 |
|  | **MMV407834** | -1.3 | 1.42 | -2.72 |
|  | **MMV007625** | -2.13 | 0.54 | -2.67 |
|  | **MMV675968** | -4.82 | -2.2 | -2.62 |
|  | **MMV020291** | -7.54 | -4.94 | -2.6 |
|  | **MMV085210** | -3.68 | -1.1 | -2.58 |
|  | **MMV688854** | -2.52 | 0.02 | -2.54 |
|  | **MMV011691** | -0.5 | 2.01 | -2.51 |
|  | **MMV676571** | -1.61 | 0.86 | -2.47 |
|  | **MMV667494** | -4.66 | -2.3 | -2.36 |
|  | **MMV020320** | -3.85 | -1.5 | -2.35 |
|  | **MMV676260** | -5.84 | -3.53 | -2.31 |
|  | **MMV676064** | -2.54 | -0.26 | -2.28 |
|  | **MMV688766** | -4.36 | -2.09 | -2.27 |
|  | **MMV006741** | -5.41 | -3.15 | -2.26 |
|  | **MMV020537** | -4.06 | -1.8 | -2.26 |
|  | **MMV020670** | -0.99 | 1.24 | -2.23 |
|  | **MMV024443** | -4.86 | -2.64 | -2.22 |
|  | **MMV010764** | -4.2 | -2 | -2.2 |
|  | **MMV688547** | -4.82 | -2.66 | -2.16 |
|  | **MMV026020** | -1.54 | 0.59 | -2.13 |
|  | **MMV687762** | -6 | -3.89 | -2.11 |
|  | **MMV007920** | -3.65 | -1.55 | -2.1 |
|  | **MMV676480** | -5.08 | -3.04 | -2.04 |
|  | **MMV688703** | -6.05 | -4.05 | -2 |
|  | **MMV028694** | -3.78 | -1.78 | -2 |
|  | **MMV019807** | -5.98 | -4.02 | -1.96 |
|  | **MMV688360** | -5.03 | -3.07 | -1.96 |
|  | **MMV676600** | -0.44 | 1.5 | -1.94 |
|  | **MMV020520** | -0.31 | 1.61 | -1.92 |
|  | **MMV661713** | -2.99 | -1.1 | -1.89 |
|  | **MMV687800** | 1.01 | 2.9 | -1.89 |
|  | **MMV000063** | -4.38 | -2.61 | -1.77 |
|  | **MMV021013** | -4.38 | -2.61 | -1.77 |
|  | **MMV392832** | -4.6 | -2.86 | -1.74 |
|  | **MMV687696** | 0.05 | 1.78 | -1.73 |
|  | **MMV689243** | -4.35 | -2.66 | -1.69 |
|  | **MMV687188** | -4.73 | -3.1 | -1.63 |
|  | **MMV200748** | -6.74 | -5.11 | -1.63 |
|  | **MMV676589** | -4.53 | -2.93 | -1.6 |
|  | **MMV687180** | -3.67 | -2.07 | -1.6 |
|  | **MMV676159** | -3.78 | -2.18 | -1.6 |
|  | **MMV024195** | 0.04 | 1.63 | -1.59 |
|  | **MMV676182** | -3.35 | -1.77 | -1.58 |
|  | **MMV023969** | -0.96 | 0.62 | -1.58 |
|  | **MMV688371** | -4.72 | -3.15 | -1.57 |
|  | **MMV021660** | -3.54 | -2.03 | -1.51 |
|  | **MMV000062** | -3.12 | -1.61 | -1.51 |
|  | **MMV1037162** | -3.14 | -1.66 | -1.48 |
|  | **MMV688798** | -4.71 | -3.23 | -1.48 |
|  | **MMV023370** | -5.8 | -4.32 | -1.48 |
|  | **MMV001561** | -3.38 | -1.91 | -1.47 |
|  | **MMV676411** | -5.27 | -3.81 | -1.46 |
|  | **MMV663250** | -3.78 | -2.33 | -1.45 |
|  | **MMV019993** | -3.21 | -1.77 | -1.44 |
|  | **MMV020710** | -3.9 | -2.5 | -1.4 |
|  | **MMV102872** | -5.62 | -4.24 | -1.38 |
|  | **MMV188296** | -5.27 | -3.91 | -1.36 |
|  | **MMV228911** | -0.53 | 0.81 | -1.34 |
|  | **MMV689758** | -4.13 | -2.79 | -1.34 |
|  | **MMV688124** | -1.25 | 0.08 | -1.33 |
|  | **MMV676605** | -0.66 | 0.66 | -1.32 |
|  | **MMV007803** | -0.83 | 0.48 | -1.31 |
|  | **MMV020517** | -4.19 | -2.89 | -1.3 |
|  | **MMV688795** | -6.13 | -4.84 | -1.29 |
|  | **MMV676401** | -5.77 | -4.48 | -1.29 |
|  | **MMV019721** | -3.57 | -2.3 | -1.27 |
|  | **MMV676162** | -0.94 | 0.31 | -1.25 |
|  | **MMV393144** | -5.8 | -4.56 | -1.24 |
|  | **MMV687248** | -4.65 | -3.45 | -1.2 |
|  | **MMV676599** | -3.54 | -2.35 | -1.19 |
|  | **MMV688994** | 0.17 | 1.35 | -1.18 |
|  | **MMV676186** | 1.76 | 2.93 | -1.17 |
|  | **MMV688775** | -3.68 | -2.54 | -1.14 |
|  | **MMV676057** | -0.68 | 0.44 | -1.12 |
|  | **MMV688774** | 0.41 | 1.51 | -1.1 |
|  | **MMV688793** | -5.09 | -3.99 | -1.1 |
|  | **MMV676472** | 0.01 | 1.1 | -1.09 |
|  | **MMV688555** | -4.93 | -3.84 | -1.09 |
|  | **MMV652003** | -4.09 | -3.03 | -1.06 |
|  | **MMV690103** | -2.47 | -1.44 | -1.03 |
|  | **MMV687776** | -4.05 | -3.02 | -1.03 |
|  | **MMV688889** | -7.09 | -6.09 | -1 |
|  | **MMV676461** | -6.41 | -5.45 | -0.96 |
|  | **MMV016136** | -4.29 | -3.35 | -0.94 |
|  | **MMV054312** | -3.97 | -3.05 | -0.92 |
|  | **MMV687254** | -4 | -3.09 | -0.91 |
|  | **MMV688776** | -4.22 | -3.36 | -0.86 |
|  | **MMV676412** | -4.11 | -3.3 | -0.81 |
|  | **MMV676191** | -6.55 | -5.74 | -0.81 |
|  | **MMV595321** | 2.13 | 2.91 | -0.78 |
|  | **MMV688313** | 0.17 | 0.94 | -0.77 |
|  | **MMV676442** | -1.06 | -0.31 | -0.75 |
|  | **MMV676444** | -6.6 | -5.87 | -0.73 |
|  | **MMV637953** | 0.9 | 1.59 | -0.69 |
|  | **MMV688514** | -5.77 | -5.11 | -0.66 |
|  | **MMV063404** | -4.42 | -3.79 | -0.63 |
|  | **MMV006833** | -4.65 | -4.04 | -0.61 |
|  | **MMV011903** | -0.38 | 0.23 | -0.61 |
|  | **MMV676377** | -6.09 | -5.48 | -0.61 |
|  | **MMV407539** | 0.68 | 1.23 | -0.55 |
|  | **MMV676398** | -5.27 | -4.74 | -0.53 |
|  | **MMV689437** | -2.87 | -2.37 | -0.5 |
|  | **MMV676350** | -4.33 | -3.84 | -0.49 |
|  | **MMV023183** | -5.97 | -5.52 | -0.45 |
|  | **MMV688470** | -1.88 | -1.43 | -0.45 |
|  | **MMV023985** | -3.88 | -3.45 | -0.43 |
|  | **MMV1088520** | -3.4 | -3 | -0.4 |
|  | **MMV009135** | -3.25 | -2.85 | -0.4 |
|  | **MMV689709** | -6.32 | -5.93 | -0.39 |
|  | **MMV688980** | -3.27 | -2.88 | -0.39 |
|  | **MMV688279** | -3.34 | -2.99 | -0.35 |
|  | **MMV020321** | -5.97 | -5.66 | -0.31 |
|  | **MMV688274** | 2.87 | 3.14 | -0.27 |
|  | **MMV675996** | -3.45 | -3.19 | -0.26 |
|  | **MMV676409** | -5.67 | -5.43 | -0.24 |
|  | **MMV020391** | -5.28 | -5.06 | -0.22 |
|  | **MMV688416** | 0.04 | 0.26 | -0.22 |
|  | **MMV687796** | 1.37 | 1.59 | -0.22 |
|  | **MMV689000** | 0.7 | 0.89 | -0.19 |
|  | **MMV202553** | -4.96 | -4.79 | -0.17 |
|  | **MMV688763** | -6.63 | -6.47 | -0.16 |
|  | **MMV026468** | -5.39 | -5.23 | -0.16 |
|  | **MMV689255** | -3.54 | -3.39 | -0.15 |
|  | **MMV019234** | 0.79 | 0.9 | -0.11 |
|  | **MMV676063** | 0.38 | 0.47 | -0.09 |
|  | **MMV676382** | -7.19 | -7.14 | -0.05 |
|  | **MMV006372** | -5.86 | -5.82 | -0.04 |
|  | **MMV062221** | -3.68 | -3.64 | -0.04 |
|  | **MMV689244** | 0.24 | 0.25 | -0.01 |
|  | **MMV676050** | -3.89 | -3.9 | 0.01 |
|  | **MMV053220** | -5.24 | -5.28 | 0.04 |
|  | **MMV085230** | -5.69 | -5.74 | 0.05 |
|  | **MMV676204** | -2.95 | -3.01 | 0.06 |
|  | **MMV688888** | 0.92 | 0.85 | 0.07 |
|  | **MMV676386** | -6.37 | -6.44 | 0.07 |
|  | **MMV676526** | -5.45 | -5.54 | 0.09 |
|  | **MMV1110498** | -3.8 | -3.9 | 0.1 |
|  | **MMV045105** | -4.88 | -5 | 0.12 |
|  | **MMV688934** | -3.98 | -4.12 | 0.14 |
|  | **MMV687170** | -3.26 | -3.4 | 0.14 |
|  | **MMV024311** | -4.88 | -5.09 | 0.21 |
|  | **MMV000011** | 1.1 | 0.85 | 0.25 |
|  | **MMV676474** | -5.83 | -6.09 | 0.26 |
|  | **MMV002816** | -5.54 | -5.82 | 0.28 |
|  | **MMV675993** | -3.98 | -4.27 | 0.29 |
|  | **MMV019838** | -4.75 | -5.05 | 0.3 |
|  | **MMV675995** | 0.61 | 0.31 | 0.3 |
|  | **MMV020388** | -3.38 | -3.68 | 0.3 |
|  | **MMV006239** | -5.6 | -5.9 | 0.3 |
|  | **MMV010545** | -5.29 | -5.6 | 0.31 |
|  | **MMV676358** | -5.72 | -6.04 | 0.32 |
|  | **MMV688125** | -3.87 | -4.23 | 0.36 |
|  | **MMV000016** | -3.61 | -4.02 | 0.41 |
|  | **MMV003270** | -5.83 | -6.24 | 0.41 |
|  | **MMV687138** | -5.91 | -6.35 | 0.44 |
|  | **MMV676008** | 1.13 | 0.66 | 0.47 |
|  | **MMV1019989** | -4.39 | -4.87 | 0.48 |
|  | **MMV084603** | -4.14 | -4.62 | 0.48 |
|  | **MMV688557** | -4.67 | -5.17 | 0.5 |
|  | **MMV688273** | -3.68 | -4.2 | 0.52 |
|  | **MMV676406** | 1.65 | 1.12 | 0.53 |
|  | **MMV688891** | -5.15 | -5.69 | 0.54 |
|  | **MMV687146** | -6.12 | -6.67 | 0.55 |
|  | **MMV658988** | -3.64 | -4.19 | 0.55 |
|  | **MMV011511** | -2.94 | -3.5 | 0.56 |
|  | **MMV687706** | -6 | -6.58 | 0.58 |
|  | **MMV011765** | -5.25 | -5.83 | 0.58 |
|  | **MMV1028806** | -5.15 | -5.73 | 0.58 |
|  | **MMV069458** | -4.84 | -5.42 | 0.58 |
|  | **MMV016838** | 0.65 | 0.06 | 0.59 |
|  | **MMV1236379** | -5.96 | -6.56 | 0.6 |
|  | **MMV690027** | 1.17 | 0.56 | 0.61 |
|  | **MMV553002** | -6.87 | -7.48 | 0.61 |
|  | **MMV676384** | -5.81 | -6.42 | 0.61 |
|  | **MMV687730** | -3.45 | -4.08 | 0.63 |
|  | **MMV688543** | -6.26 | -6.9 | 0.64 |
|  | **MMV676588** | -4.91 | -5.56 | 0.65 |
|  | **MMV687273** | -7.05 | -7.71 | 0.66 |
|  | **MMV019551** | -4.42 | -5.08 | 0.66 |
|  | **MMV020120** | -6.81 | -7.48 | 0.67 |
|  | **MMV688548** | -6.15 | -6.83 | 0.68 |
|  | **MMV002529** | -5.03 | -5.71 | 0.68 |
|  | **MMV676584** | -4.48 | -5.18 | 0.7 |
|  | **MMV1030799** | -6.27 | -6.97 | 0.7 |
|  | **MMV659010** | -6.13 | -6.83 | 0.7 |
|  | **MMV001625** | -6.58 | -7.31 | 0.73 |
|  | **MMV676477** | 1.72 | 0.99 | 0.73 |
|  | **MMV676468** | -5.31 | -6.05 | 0.74 |
|  | **MMV676395** | -5.04 | -5.79 | 0.75 |
|  | **MMV688553** | -5.66 | -6.42 | 0.76 |
|  | **MMV676439** | -6.4 | -7.17 | 0.77 |
|  | **MMV000023** | -3.28 | -4.06 | 0.78 |
|  | **MMV690028** | 1.66 | 0.86 | 0.8 |
|  | **MMV676379** | -3.18 | -3.99 | 0.81 |
|  | **MMV023227** | -5.97 | -6.81 | 0.84 |
|  | **MMV020152** | -5.47 | -6.31 | 0.84 |
|  | **MMV495543** | -5.73 | -6.6 | 0.87 |
|  | **MMV021375** | -4.42 | -5.33 | 0.91 |
|  | **MMV688852** | -4.28 | -5.2 | 0.92 |
|  | **MMV688978** | -3.78 | -4.7 | 0.92 |
|  | **MMV676383** | -6.06 | -6.98 | 0.92 |
|  | **MMV688853** | -5.1 | -6.07 | 0.97 |
|  | **MMV688958** | -6.04 | -7.05 | 1.01 |
|  | **MMV687803** | -5.77 | -6.78 | 1.01 |
|  | **MMV676520** | -5.54 | -6.56 | 1.02 |
|  | **MMV024101** | -4.58 | -5.6 | 1.02 |
|  | **MMV047015** | -3.18 | -4.21 | 1.03 |
|  | **MMV020081** | -4.74 | -5.78 | 1.04 |
|  | **MMV272144** | -3.92 | -4.99 | 1.07 |
|  | **MMV676161** | -6.73 | -7.83 | 1.1 |
|  | **MMV153413** | -3.89 | -5 | 1.11 |
|  | **MMV393995** | -4.89 | -6.02 | 1.13 |
|  | **MMV688756** | -5.14 | -6.28 | 1.14 |
|  | **MMV084864** | -3.29 | -4.44 | 1.15 |
|  | **MMV687807** | -4.42 | -5.6 | 1.18 |
|  | **MMV688411** | -1.59 | -2.8 | 1.21 |
|  | **MMV676524** | -4.96 | -6.21 | 1.25 |
|  | **MMV026356** | -3.25 | -4.5 | 1.25 |
|  | **MMV688797** | -5.67 | -6.98 | 1.31 |
|  | **MMV688352** | 1.97 | 0.62 | 1.35 |
|  | **MMV688122** | -4.25 | -5.62 | 1.37 |
|  | **MMV688943** | -5.36 | -6.77 | 1.41 |
|  | **MMV001499** | -5.91 | -7.32 | 1.41 |
|  | **MMV611037** | -5.12 | -6.53 | 1.41 |
|  | **MMV688955** | -4.19 | -5.61 | 1.42 |
|  | **MMV002817** | -5.69 | -7.12 | 1.43 |
|  | **MMV687747** | -6.15 | -7.61 | 1.46 |
|  | **MMV023388** | -5.17 | -6.63 | 1.46 |
|  | **MMV676431** | -6.69 | -8.19 | 1.5 |
|  | **MMV687699** | -5.08 | -6.58 | 1.5 |
|  | **MMV687251** | -4.48 | -5.98 | 1.5 |
|  | **MMV009054** | -2.99 | -4.5 | 1.51 |
|  | **MMV688330** | -2.98 | -4.5 | 1.52 |
|  | **MMV676048** | -2.66 | -4.19 | 1.53 |
|  | **MMV688469** | -5.29 | -6.83 | 1.54 |
|  | **MMV687801** | -3.74 | -5.29 | 1.55 |
|  | **MMV688410** | -2.57 | -4.12 | 1.55 |
|  | **MMV011229** | -3.94 | -5.51 | 1.57 |
|  | **MMV026313** | -5.31 | -6.88 | 1.57 |
|  | **MMV687765** | 3.45 | 1.83 | 1.62 |
|  | **MMV689060** | -5.36 | -6.99 | 1.63 |
|  | **MMV146306** | -5.25 | -6.88 | 1.63 |
|  | **MMV031011** | -3.33 | -5 | 1.67 |
|  | **MMV687749** | 3.22 | 1.54 | 1.68 |
|  | **MMV003152** | -4.41 | -6.1 | 1.69 |
|  | **MMV676492** | -3.25 | -4.96 | 1.71 |
|  | **MMV688938** | -5.13 | -6.92 | 1.79 |
|  | **MMV676445** | -4.28 | -6.1 | 1.82 |
|  | **MMV676470** | -5.62 | -7.45 | 1.83 |
|  | **MMV637229** | -3.85 | -5.76 | 1.91 |
|  | **MMV022236** | -2.3 | -4.22 | 1.92 |
|  | **MMV688554** | -5.47 | -7.45 | 1.98 |
|  | **MMV687243** | -6.65 | -8.67 | 2.02 |
|  | **MMV161996** | -3.22 | -5.26 | 2.04 |
|  | **MMV676881** | -3.36 | -5.4 | 2.04 |
|  | **MMV000907** | -4.07 | -6.12 | 2.05 |
|  | **MMV676269** | -5.58 | -7.75 | 2.17 |
|  | **MMV688417** | -4.22 | -6.39 | 2.17 |
|  | **MMV688846** | -6.22 | -8.39 | 2.17 |
|  | **MMV688755** | -3.77 | -5.99 | 2.22 |
|  | **MMV676388** | -5.73 | -8.01 | 2.28 |
|  | **MMV676877** | -4.24 | -6.55 | 2.31 |
|  | **MMV012074** | -5.12 | -7.44 | 2.32 |
|  | **MMV687145** | -6.39 | -8.72 | 2.33 |
|  | **MMV688773** | -3.53 | -5.9 | 2.37 |
|  | **MMV676449** | -4.98 | -7.4 | 2.42 |
|  | **MMV688771** | -5.38 | -7.91 | 2.53 |
|  | **MMV675994** | -6.27 | -8.8 | 2.53 |
|  | **MMV026550** | -4.9 | -7.5 | 2.6 |
|  | **MMV688415** | -2.95 | -5.6 | 2.65 |
|  | **MMV676270** | -5.3 | -8.01 | 2.71 |
|  | **MMV032995** | -4.18 | -6.93 | 2.75 |
|  | **MMV689480** | -4.3 | -7.06 | 2.76 |
|  | **MMV461553** | -3.68 | -6.45 | 2.77 |
|  | **MMV676389** | -6.85 | -9.66 | 2.81 |
|  | **MMV021057** | -3.65 | -6.5 | 2.85 |
|  | **MMV688345** | -4.04 | -6.93 | 2.89 |
|  | **MMV676501** | -3.85 | -6.98 | 3.13 |
|  | **MMV688939** | -4.15 | -7.3 | 3.15 |
|  | **MMV675969** | -5.57 | -8.73 | 3.16 |
|  | **MMV001059** | -5.48 | -8.67 | 3.19 |
|  | **MMV676053** | -5.38 | -8.7 | 3.32 |
|  | **MMV688941** | -5.76 | -9.23 | 3.47 |
|  | **MMV676604** | -4.7 | -8.21 | 3.51 |
|  | **MMV688754** | 0.16 | -3.55 | 3.71 |
|  | **MMV676512** | -4.27 | -8.15 | 3.88 |
|  | **MMV202458** | -4.27 | -8.76 | 4.49 |
|  | **MMV688467** | 2.52 | -4.29 | 6.81 |

***Supplementary Table S2: Predicted ADME properties of top hundred compounds identified from initial in-silico screening.*** *The compounds fulfilling the criteria of drug likeliness by falling completely in the pink zone of bioavailability radar are considered as drug-like molecule and these are further evaluated for their activity against recombinant PfUCHL3 in-vitro. Cells representing the compounds that accord with drug-likeness criteria are highlighted in light grey and potential hits identified in this study are presented in red font.*

| **S.No** | **Name** | **Bioavailability Radar** | **Log P_o/w_** | **Log S** | **Log K_p_ (cm/s)** | **Lipinski** | **GI absorption** | **PAINS** |
| --- | --- | --- | --- | --- | --- | --- | --- | --- |
|  | **MMV023860** | 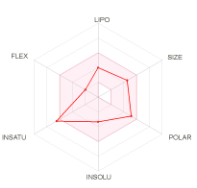 | 2.27 | -3.33 | -5.54 | Yes  0 violation | High | 0  alert |
|  | **MMV024114** | 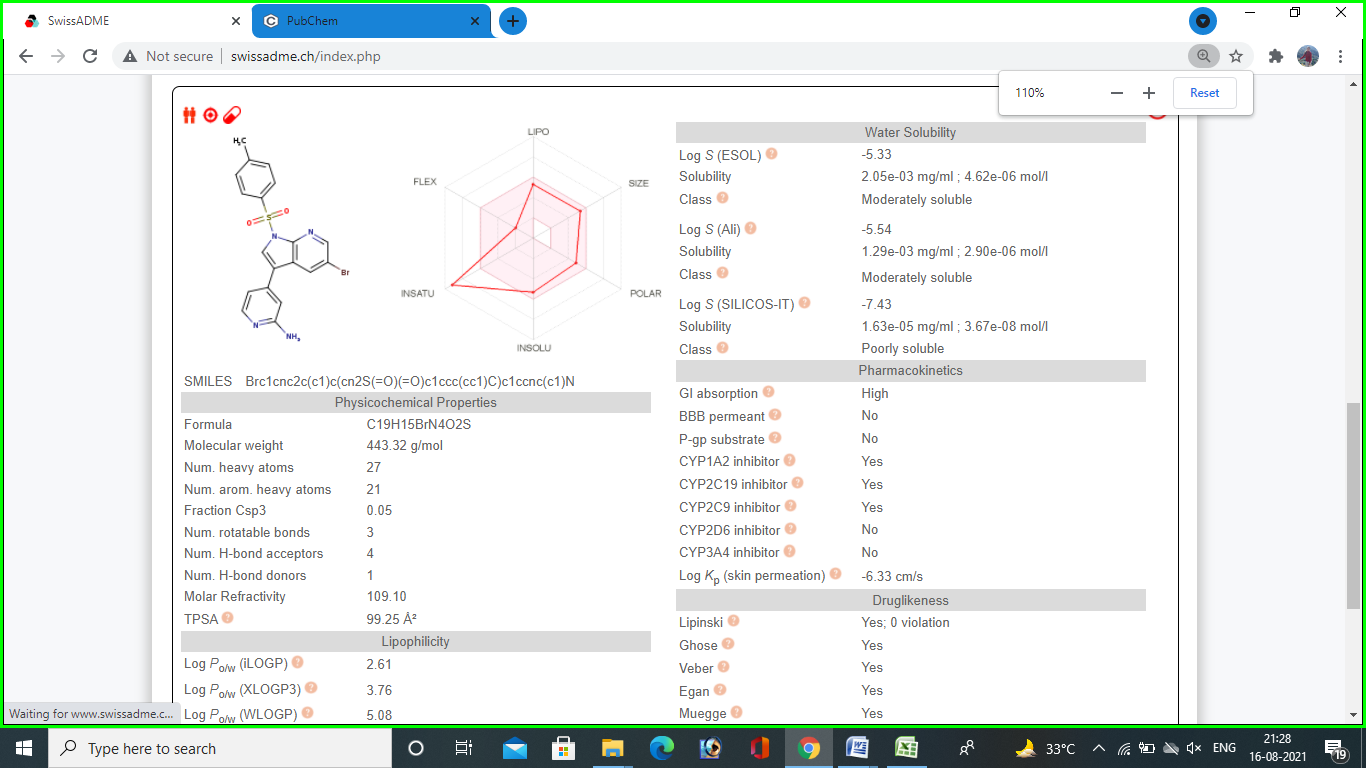 | 2.61 | -5.33 | -6.33 | Yes  0 violation | High | 0  alert |
|  | **MMV687700** | 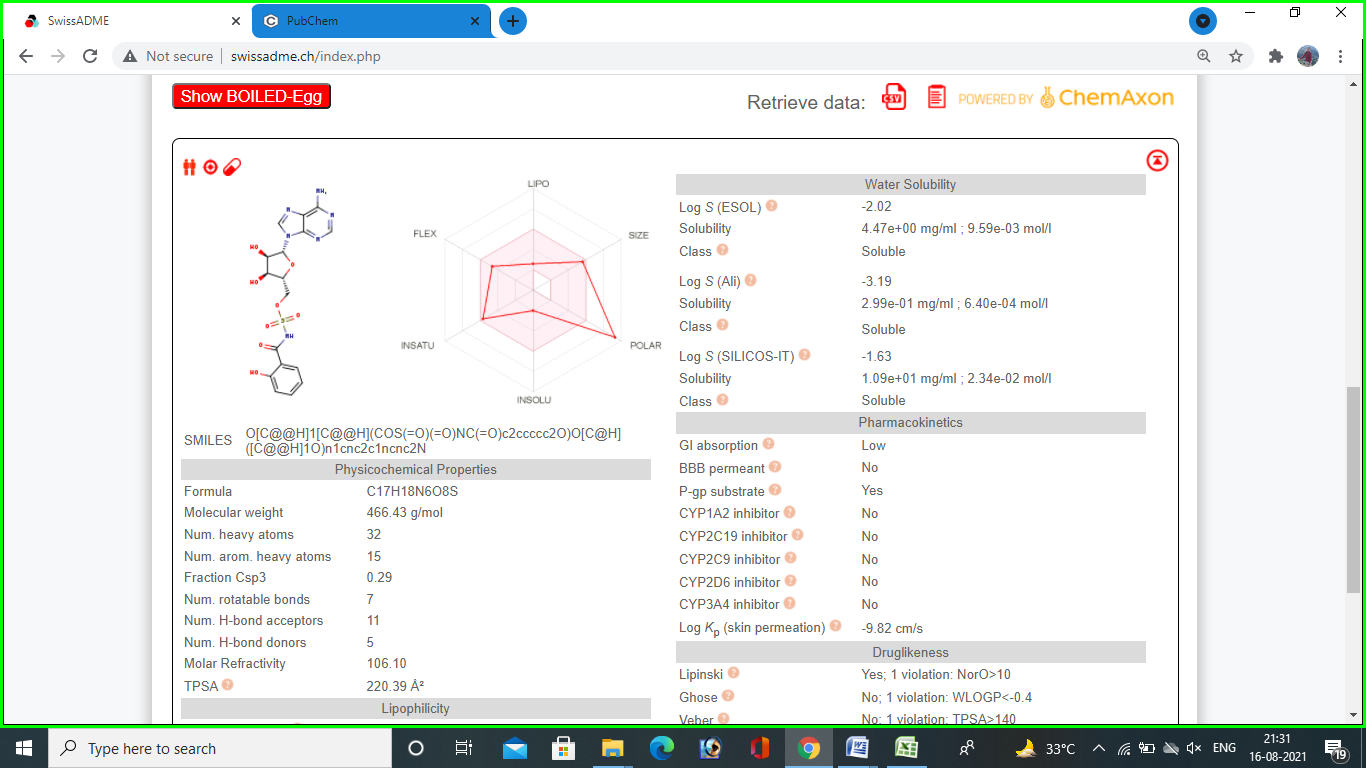 | 1.47 | -2.02 | -9.82 | No, 1 violation | Low | 0  alert |
|  | **MMV022029** | 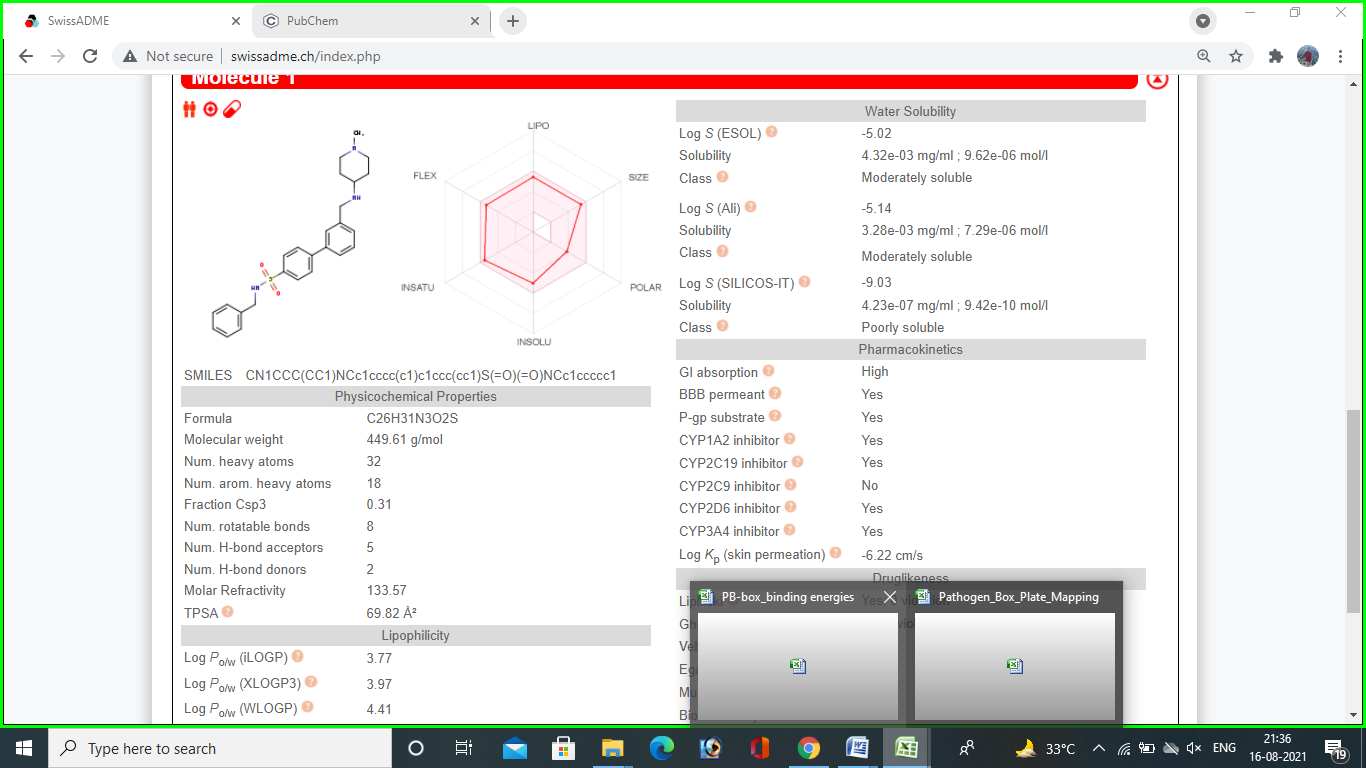 | 3.77 | -5.02 | -6.22 | Yes  0 violation | High | 0  alert |
|  | **MMV676603** | **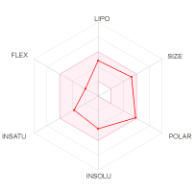** | **2.88** | **-4.41** | **-6.87** | **Yes**  **0 violation** | **High** | **0**  **alert** |
|  | **MMV688509** | 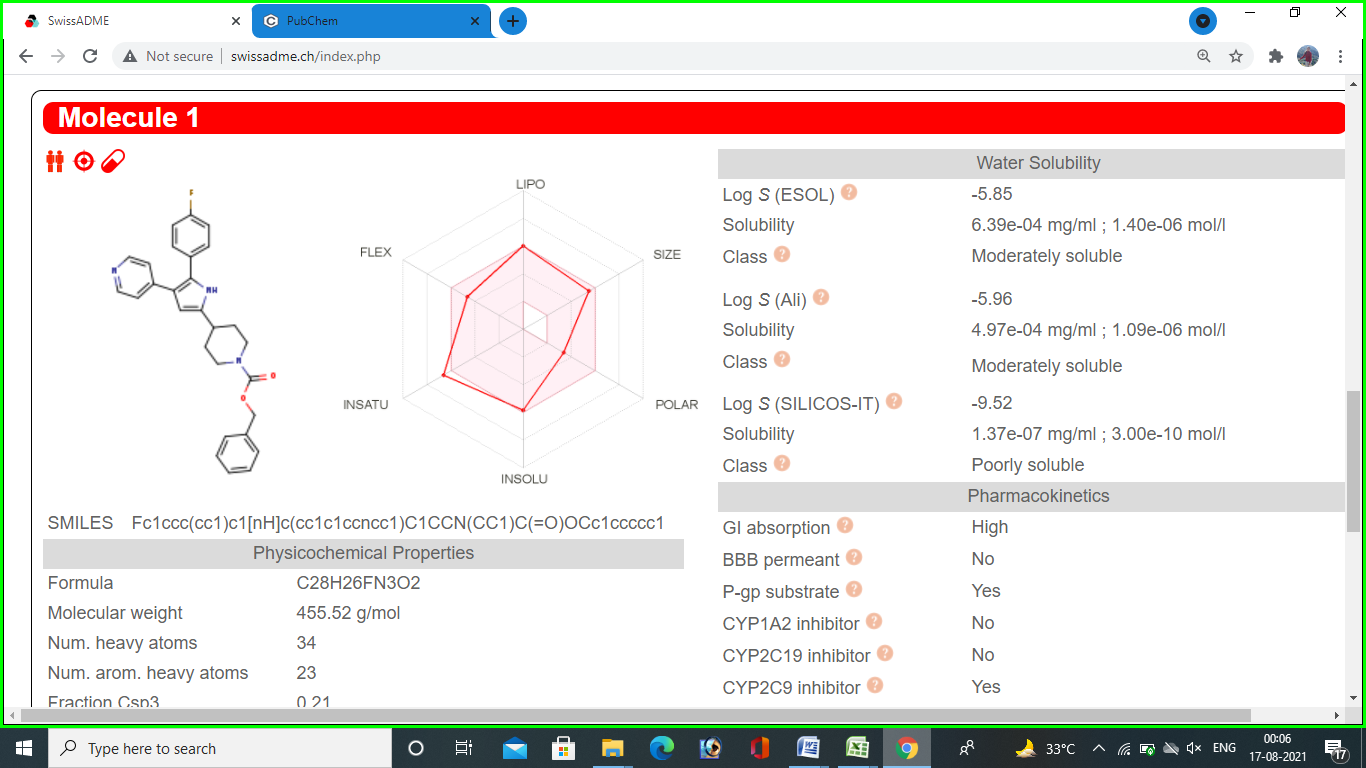 | 4.14 | -5.85 | -5.53 | Yes  0 violation | High | 0  alert |
|  | **MMV000858** | 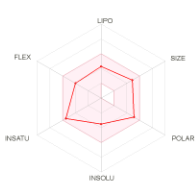 | 2.87 | -3.50 | -7.33 | Yes  0 violation | High | 0  alert |
|  | **MMV085071** | 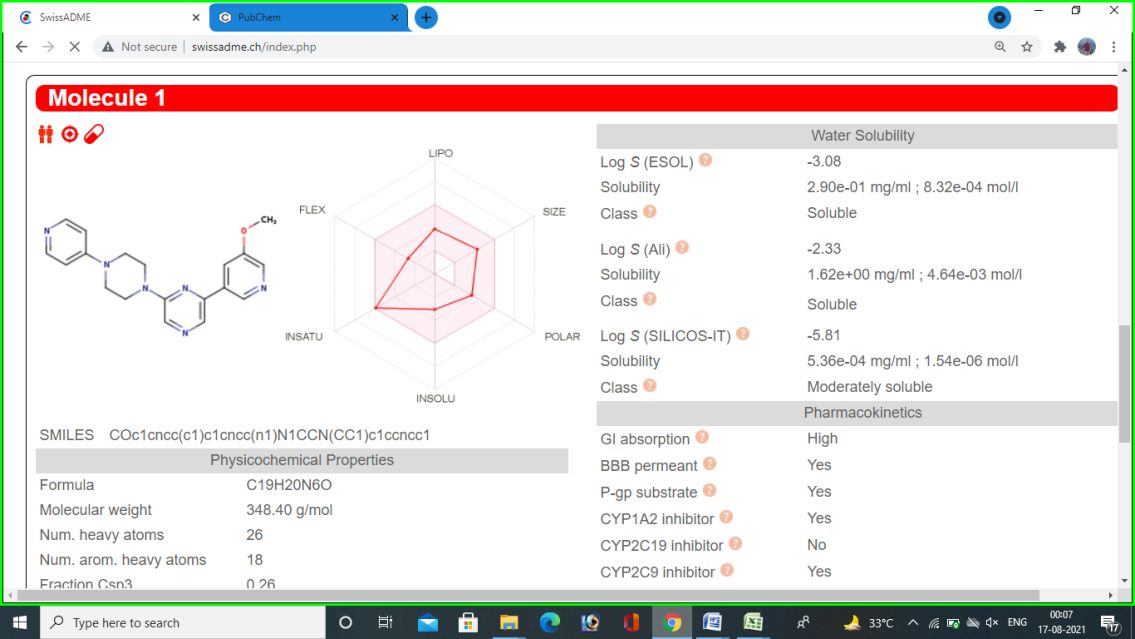 | 2.44 | -3.08 | -7.49 | Yes  0 violation | High | 0  alert |
|  | **MMV676539** | 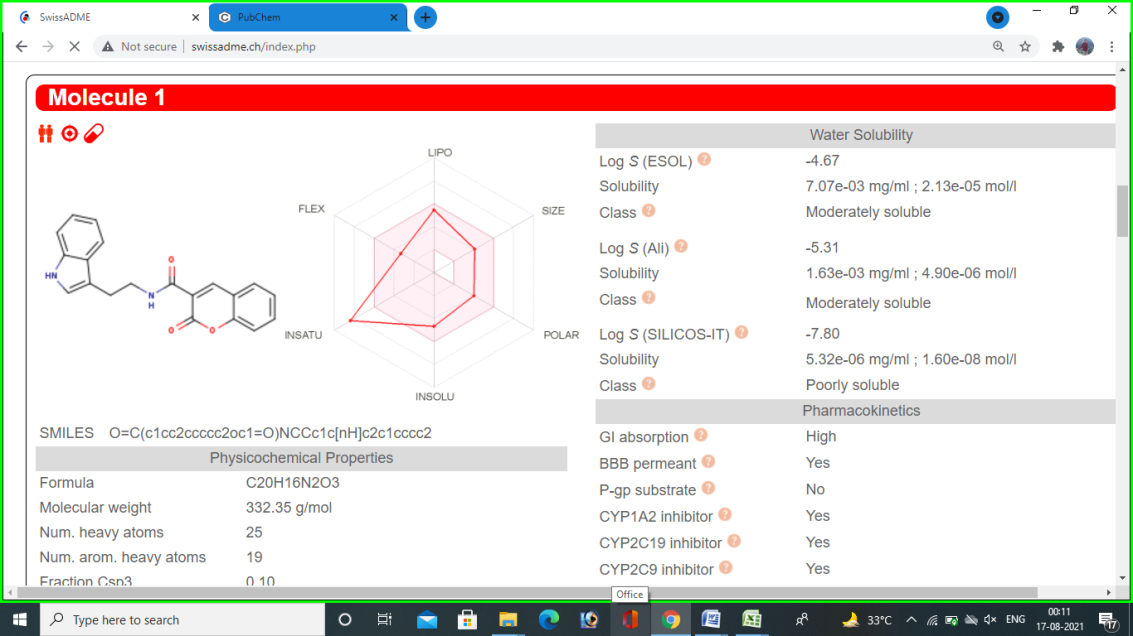 | 2.67 | -4.67 | -5.47 | Yes  0 violation | High | 0  alert |
|  | **MMV023953** | 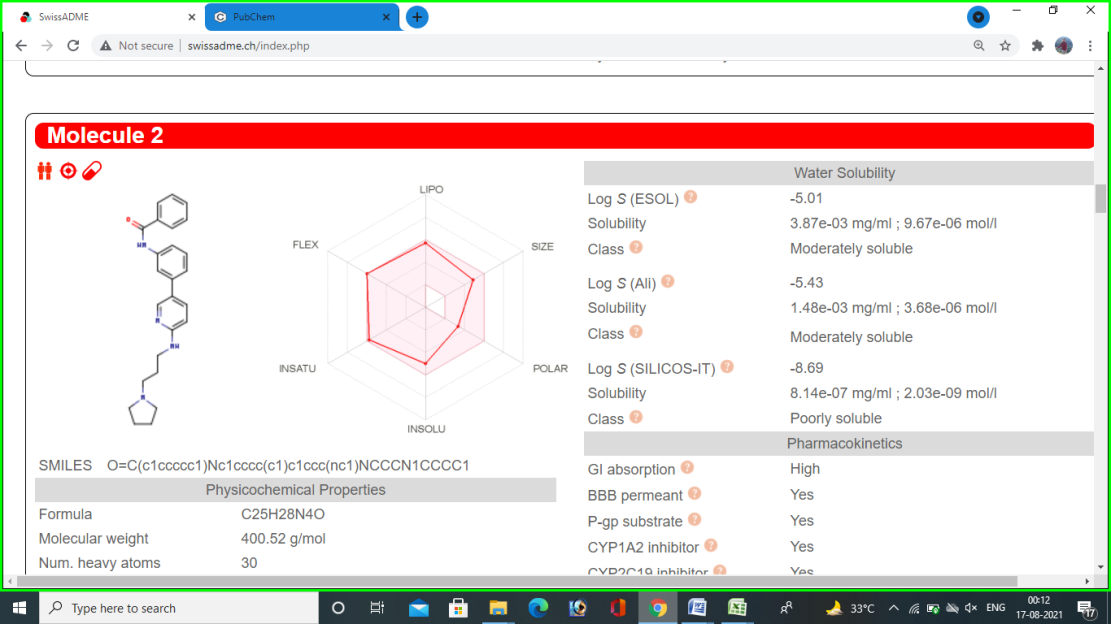 | 3.71 | -5.01 | -5.54 | Yes  0 violation | High | 0  alert |
|  | **MMV020289** | 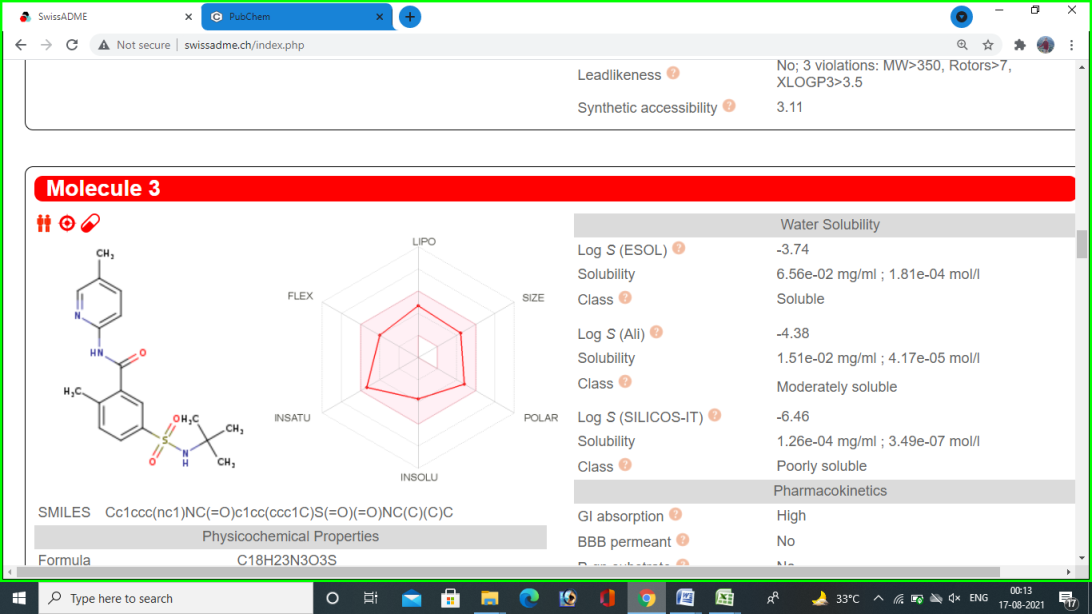 | 2.60 | -3.74 | -6.59 | Yes  0 violation | High | 0  alert |
|  | **MMV658993** | 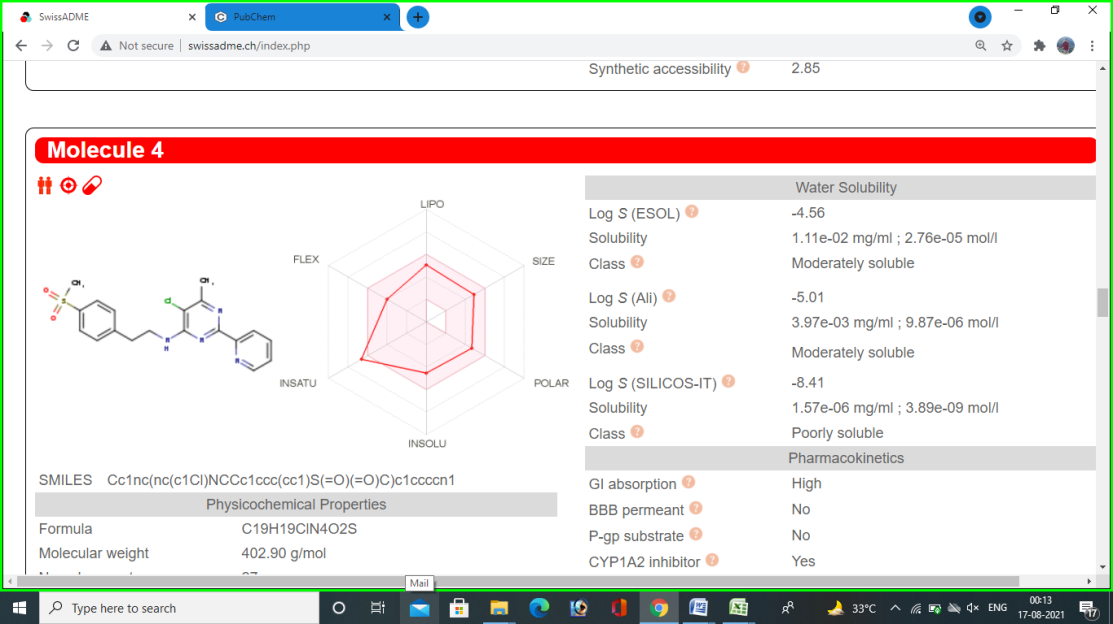 | 2.97 | -4.56 | -6.36 | Yes  0 violation | High | 0  alert |
|  | **MMV676528** | 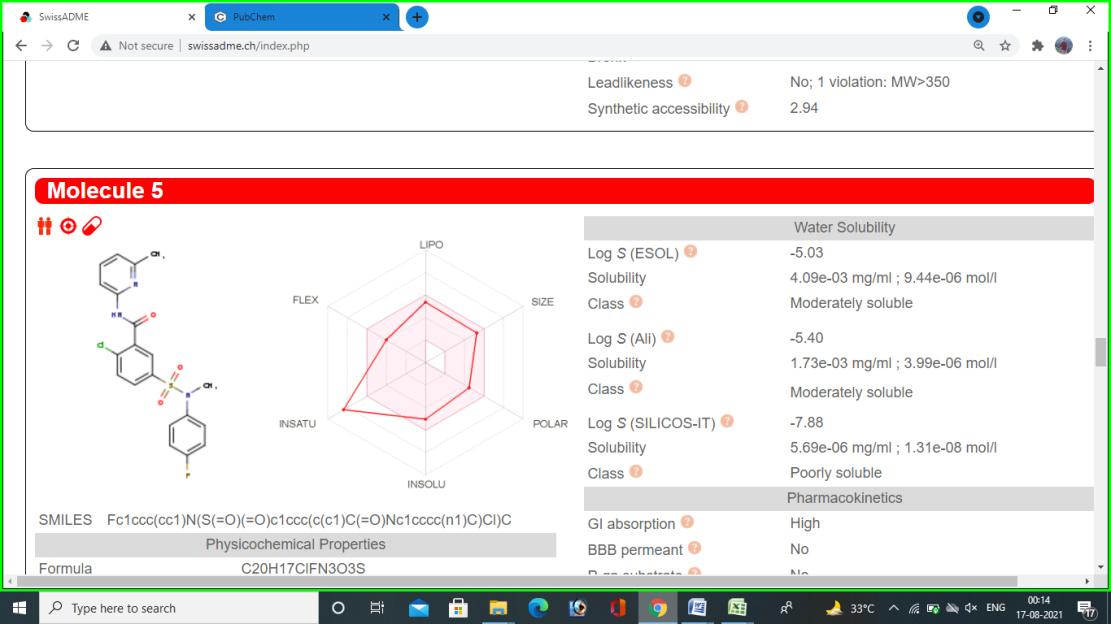 | 2.54 | -5.03 | -6.21 | Yes  0 violation | High | 0  alert |
|  | **MMV687239** | 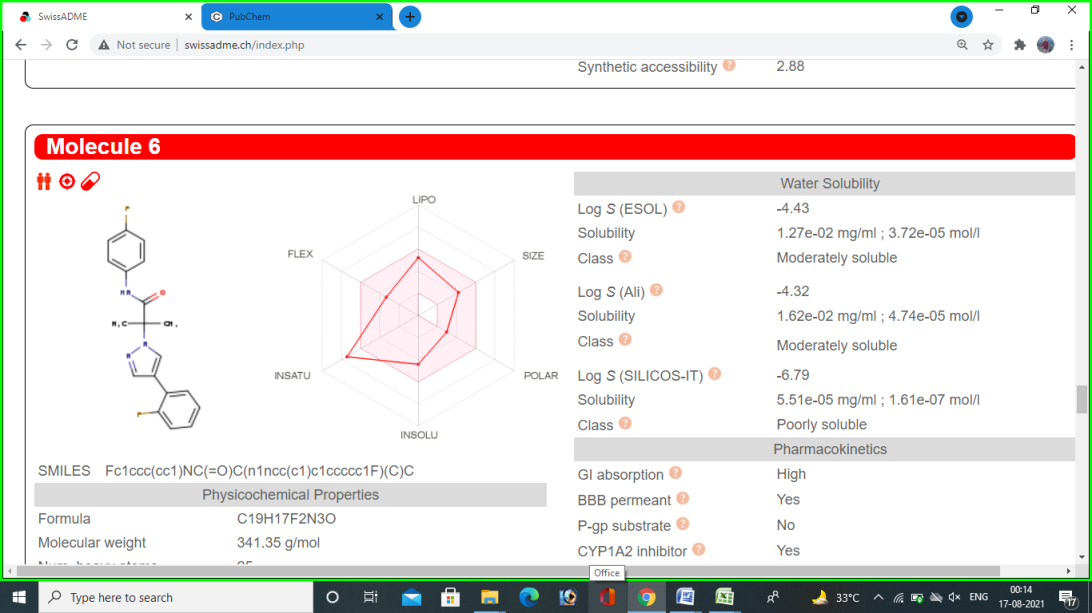 | 2.89 | -4.43 | -5.79 | Yes  0 violation | High | 0  alert |
|  | **MMV007638** | 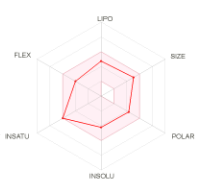 | 2.35 | -5.10 | -5.73 | Yes  0 violation | High | 0  alert |
|  | **MMV688361** | 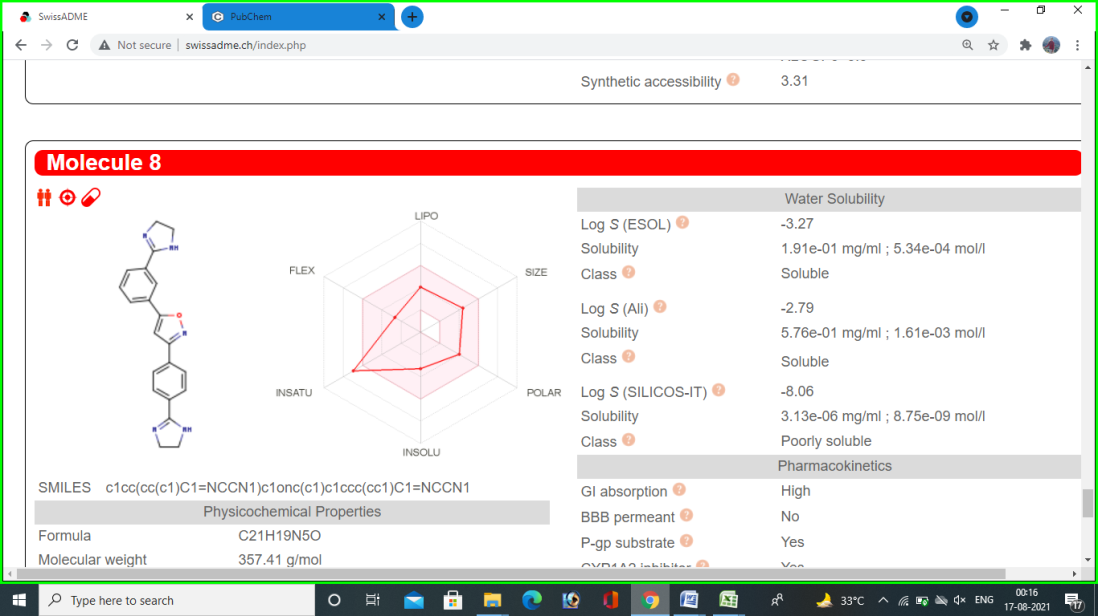 | 2.89 | -3.27 | -7.34 | Yes  0 violation | High | 0  alert |
|  | **MMV007471** | 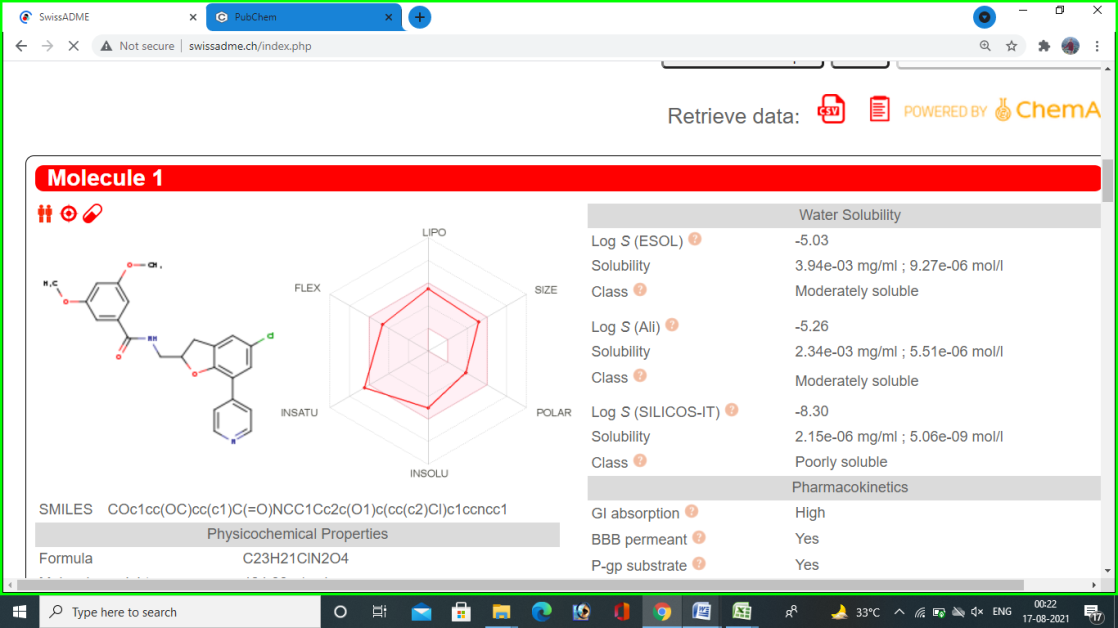 | 3.80 | -5.03 | -5.99 | Yes  0 violation | High | 0  alert |
|  | **MMV676380** | 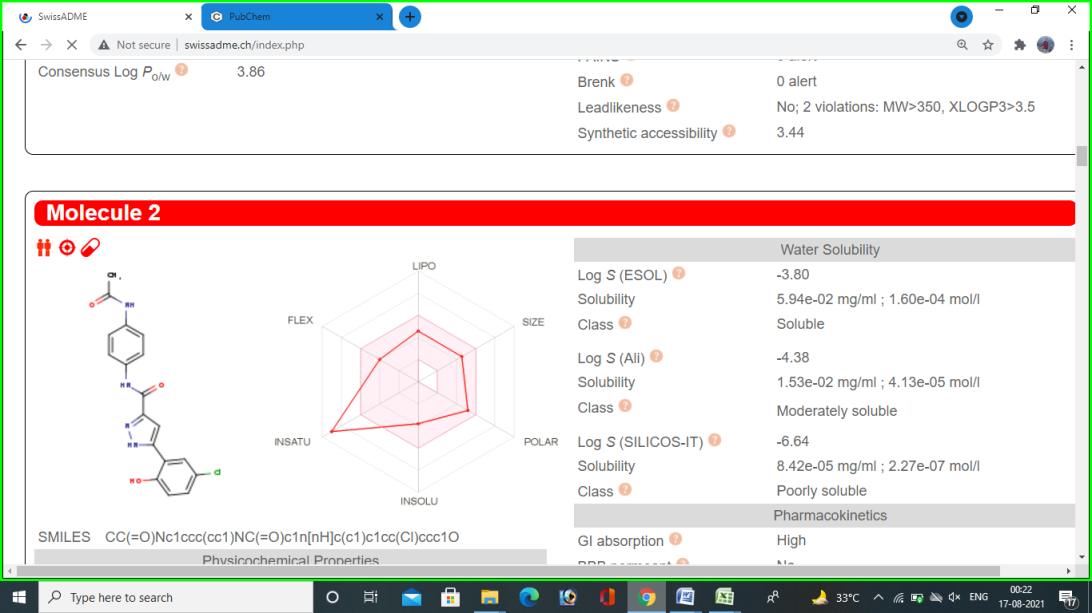 | 1.45 | -3.80 | -6.79 | Yes  0 violation | High | 0  alert |
|  | **MMV688704** | **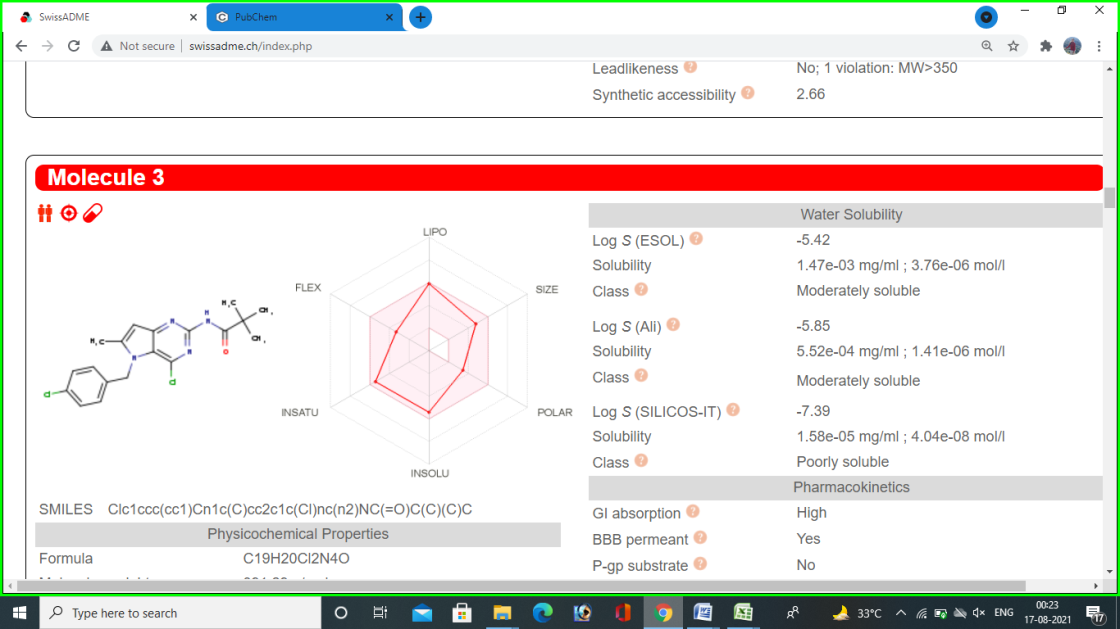** | **3.57** | **-5.42** | **-5.24** | **Yes**  **0 violation** | **High** | **0**  **alert** |
|  | **MMV010576** | 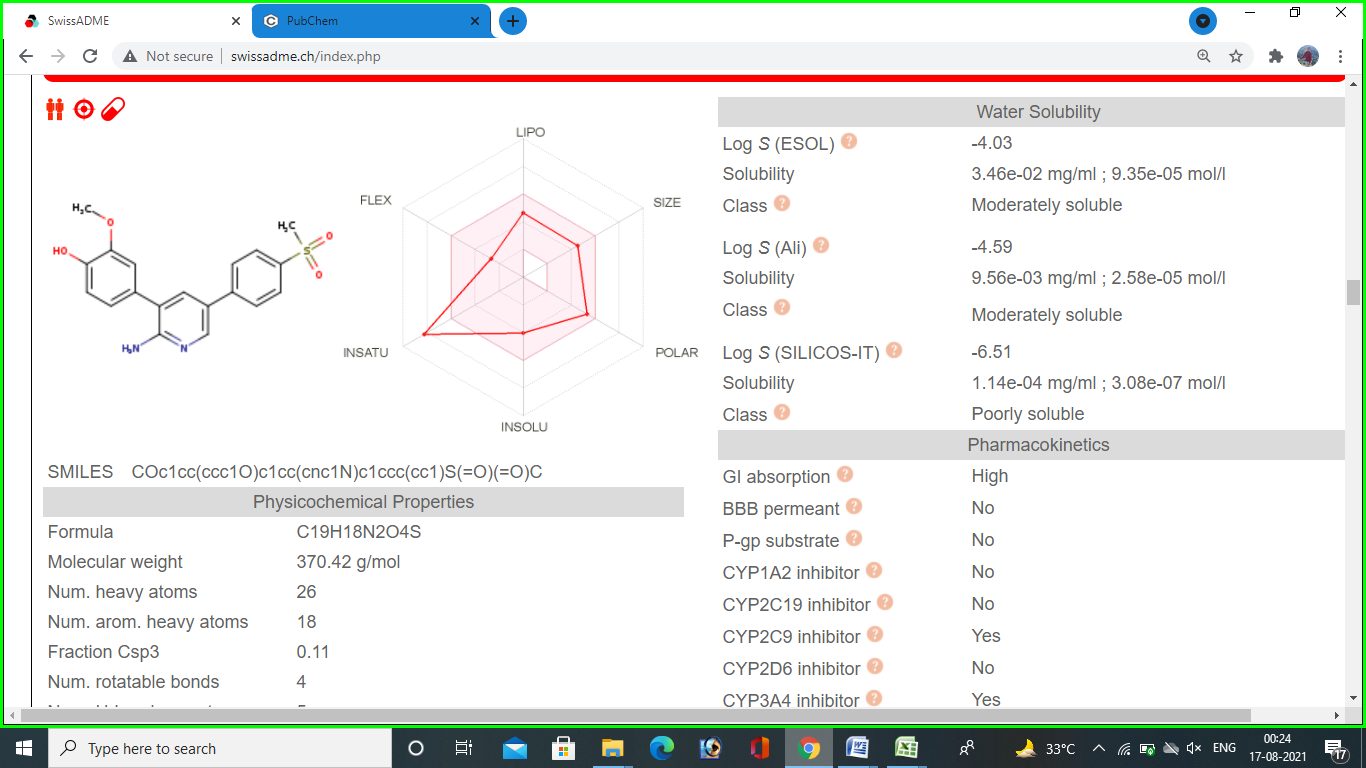 | 2.28 | -4.03 | -6.71 | Yes  0 violation | High | 0  alert |
|  | **MMV634140** | 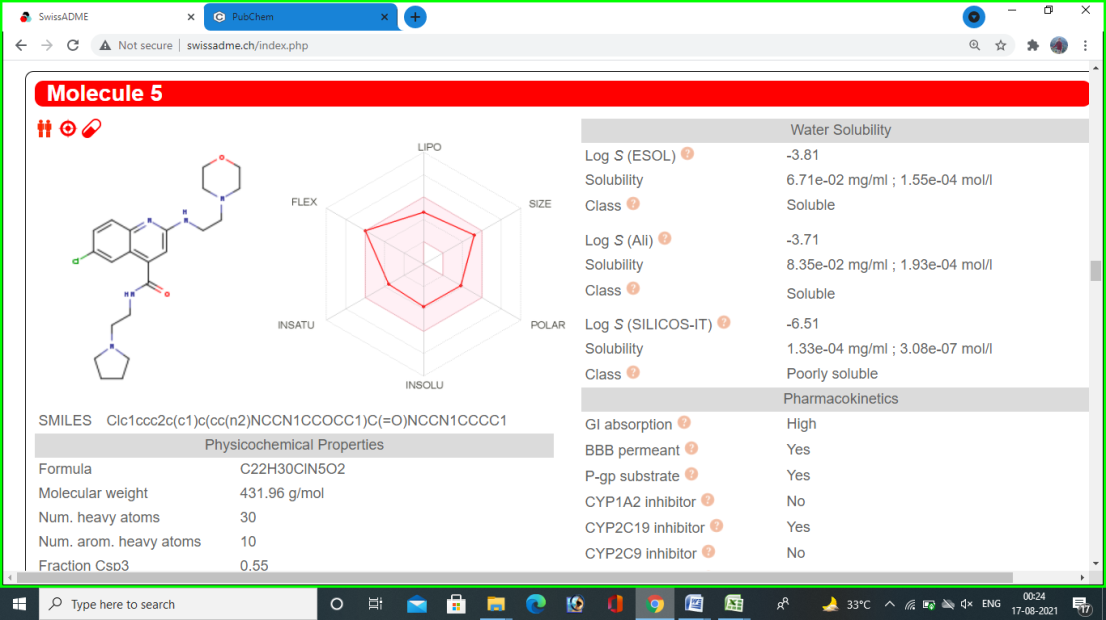 | 4.03 | -3.81 | -7.09 | Yes  0 violation | High | 0  alert |
|  | **MMV688761** | 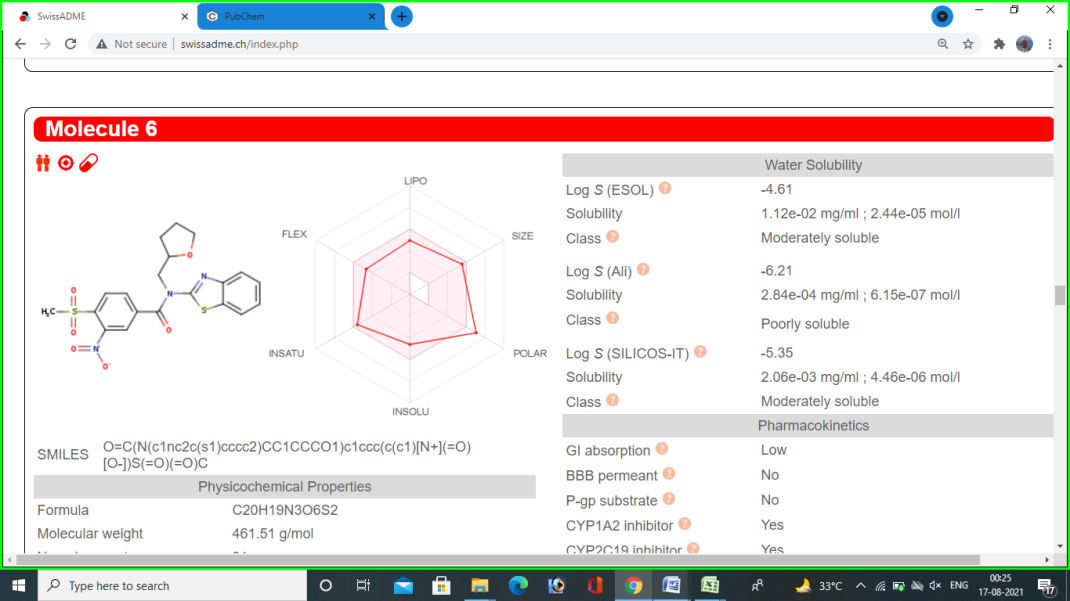 | 2.70 | -4.61 | -6.84 | Yes  0 violation | Low | 0  alert |
|  | **MMV687798** | 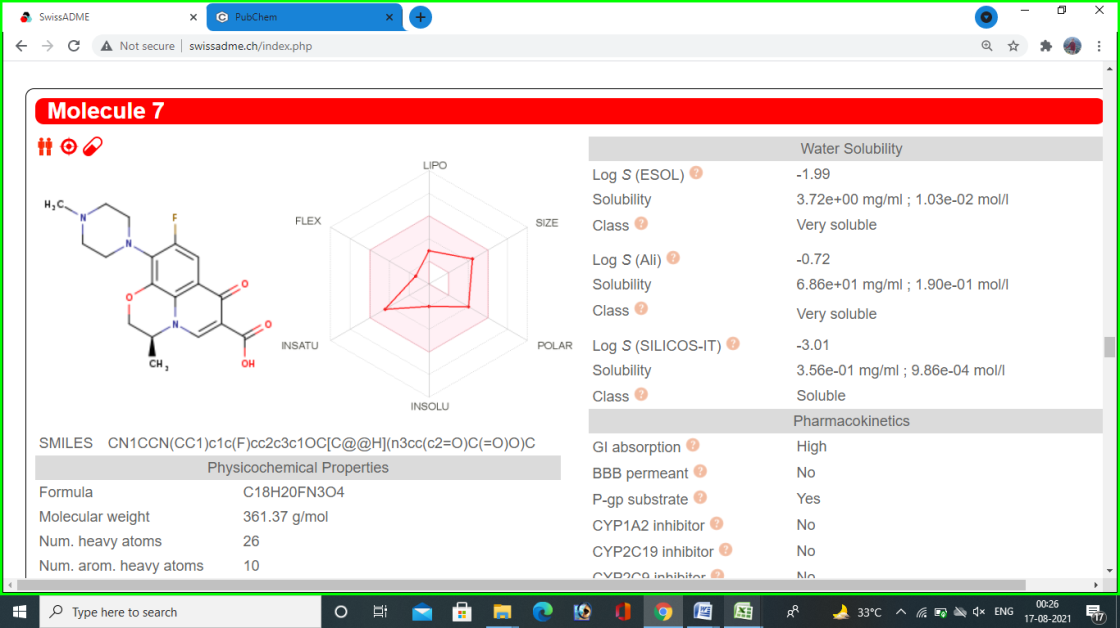 | 2.25 | -1.99 | -8.78 | Yes  0 violation | High | 0  alert |
|  | **MMV024937** | 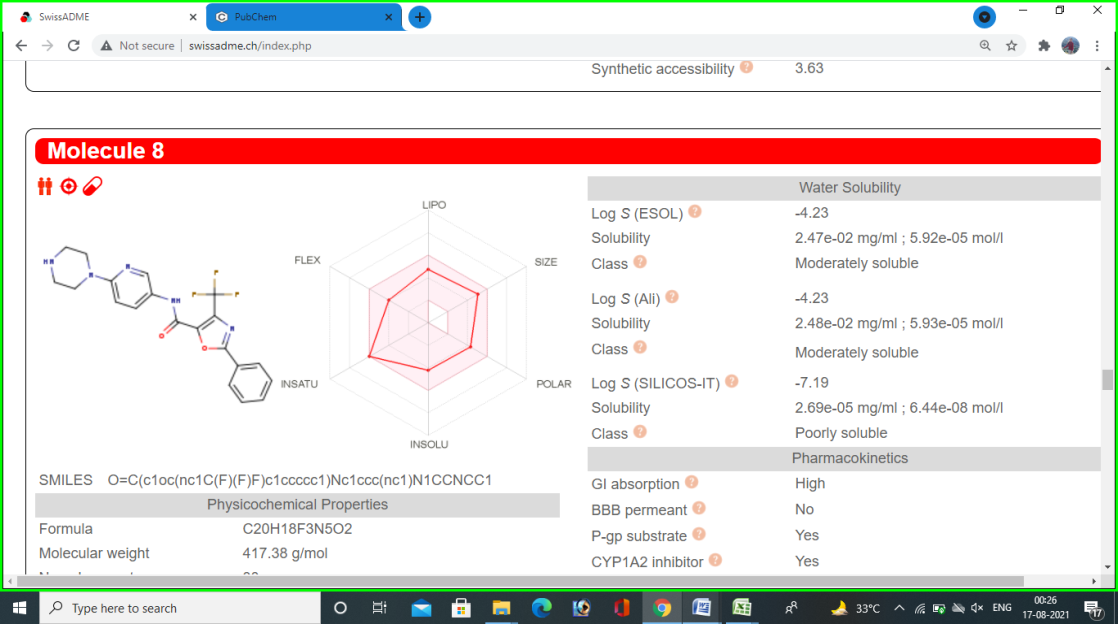 | 3.13 | -4.23 | -6.84 | Yes  0 violation | High | 0  alert |
|  | **MMV688796** | 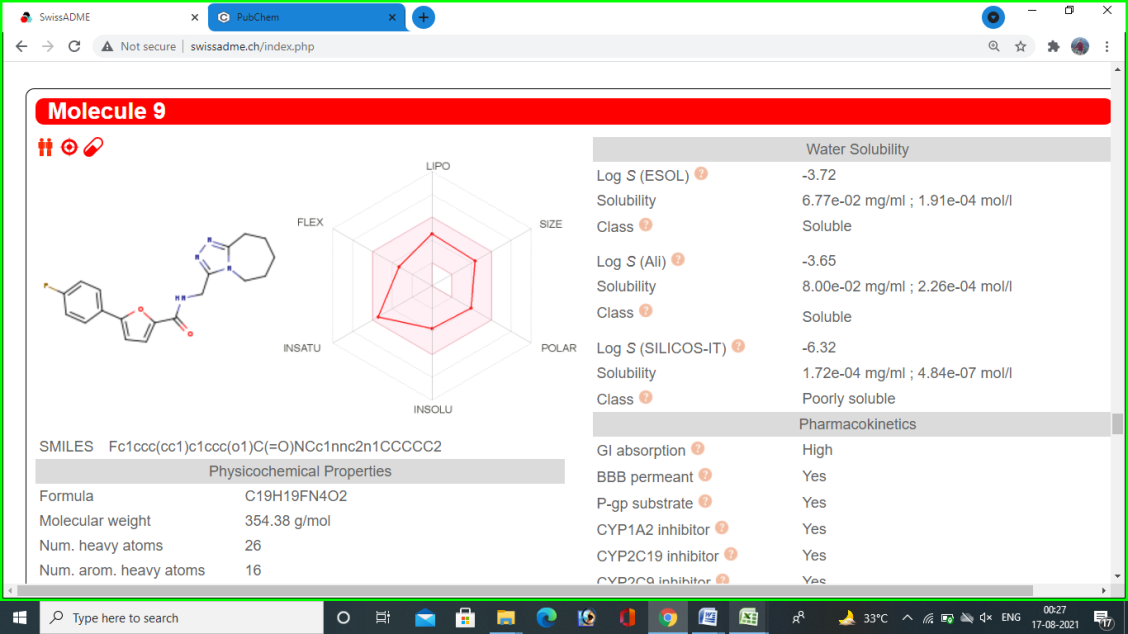 | 3.04 | -3.72 | -6.71 | Yes  0 violation | High | 0  alert |
|  | **MMV020591** | 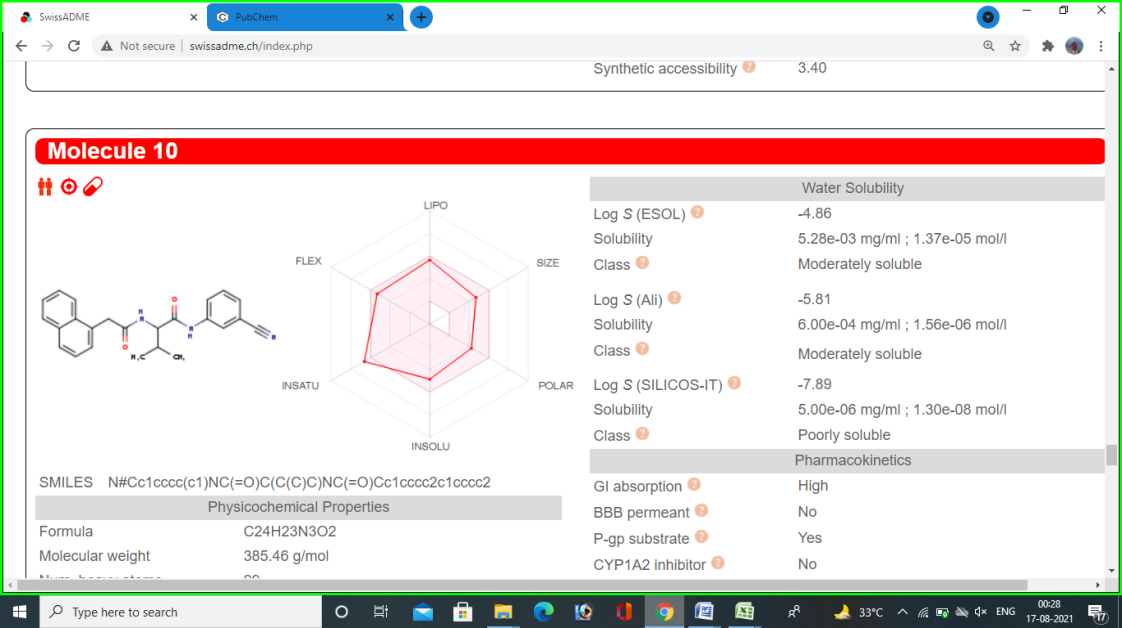 | 2.50 | -4.86 | -5.55 | Yes  0 violation | High | 0  alert |
|  | **MMV688178** | 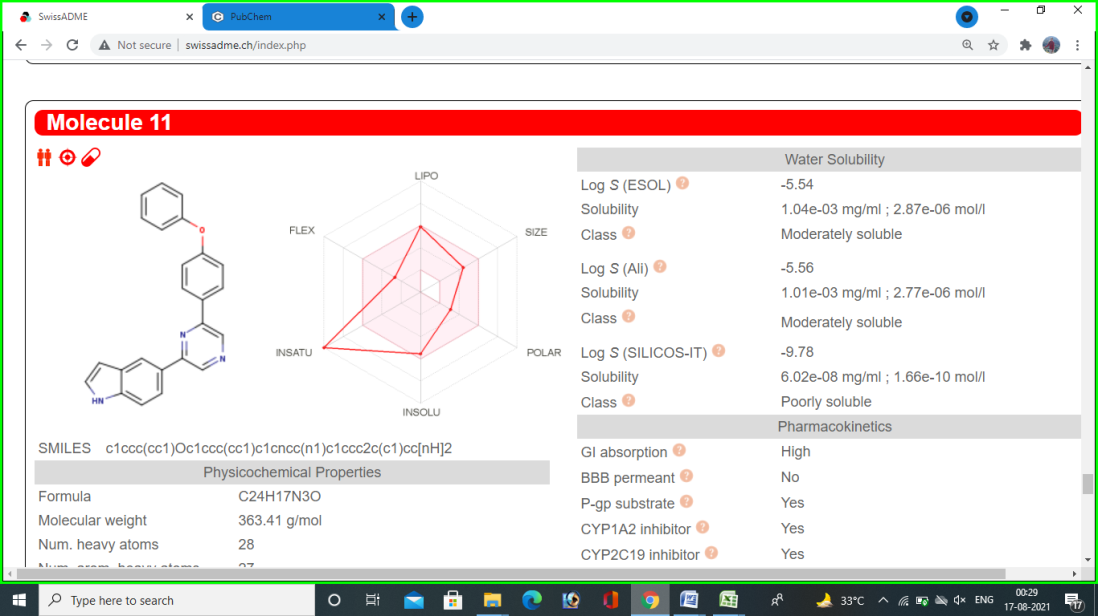 | 3.31 | -5.54 | -5.14 | Yes  0 violation | High | 0  alert |
|  | **MMV007133** | 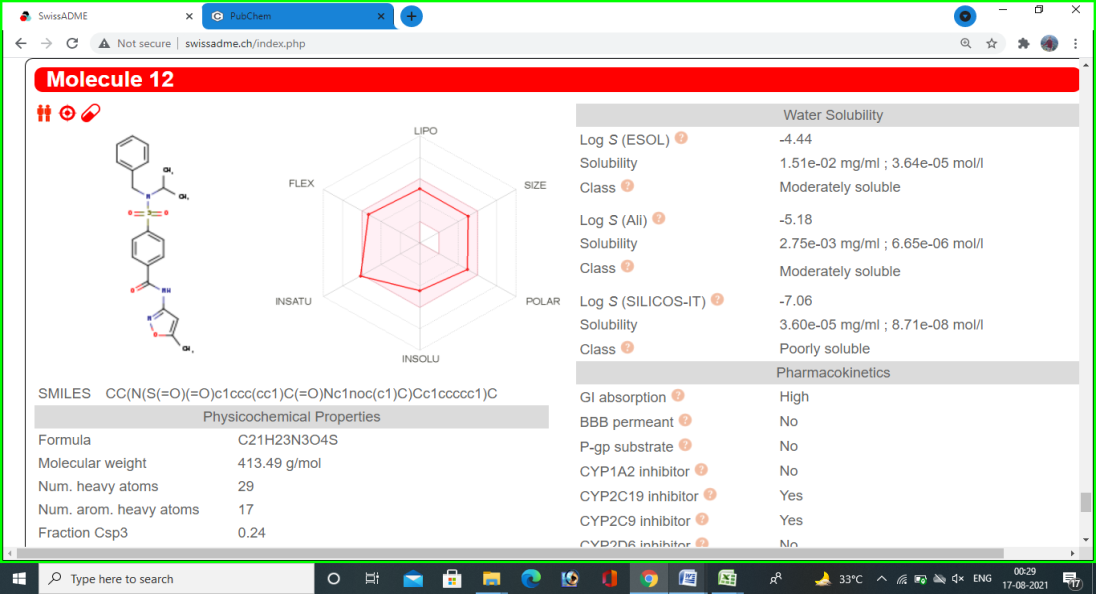 | 2.39 | -4.44 | -6.42 | Yes  0 violation | High | 0  alert |
|  | **MMV676476** | 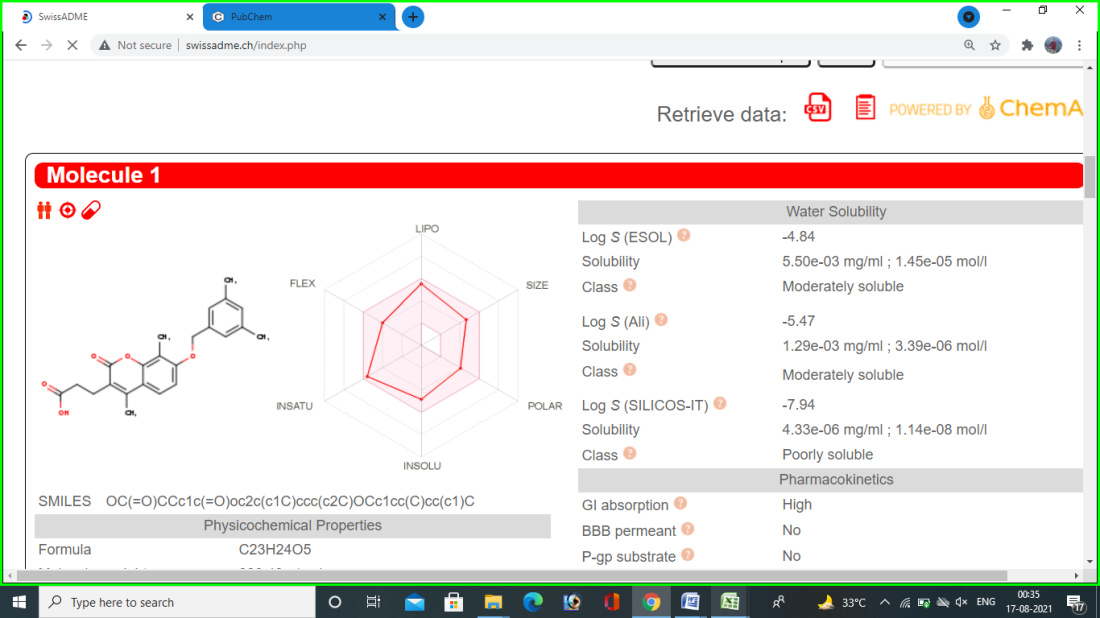 | 3.39 | -4.84 | -5.67 | Yes  0 violation | High | 0  alert |
|  | **MMV688472** | 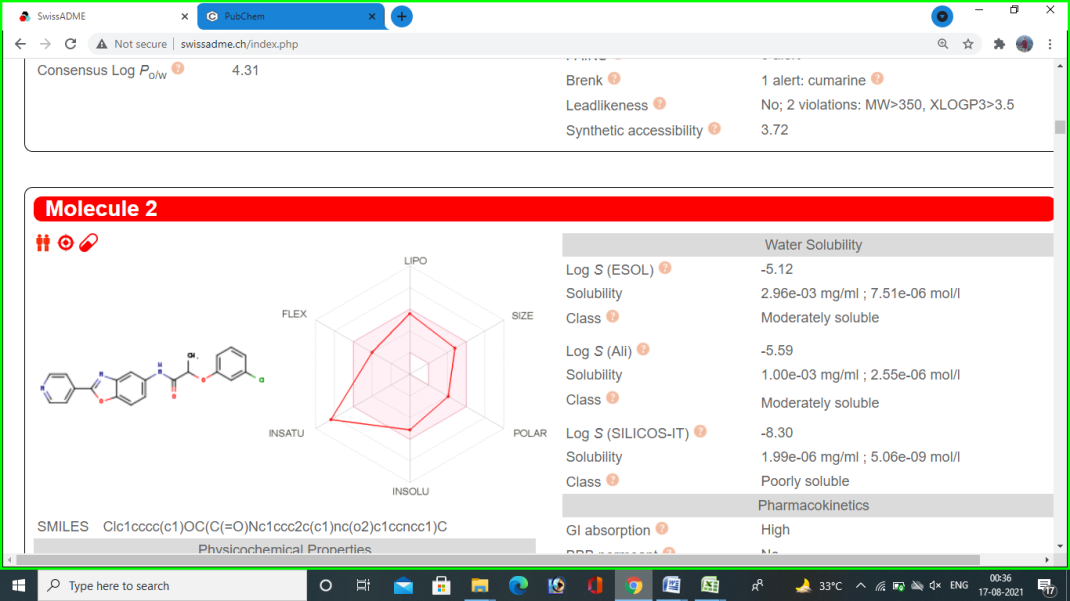 | 3.43 | -5.12 | -5.68 | Yes  0 violation | High | 0  alert |
|  | **MMV659004** | 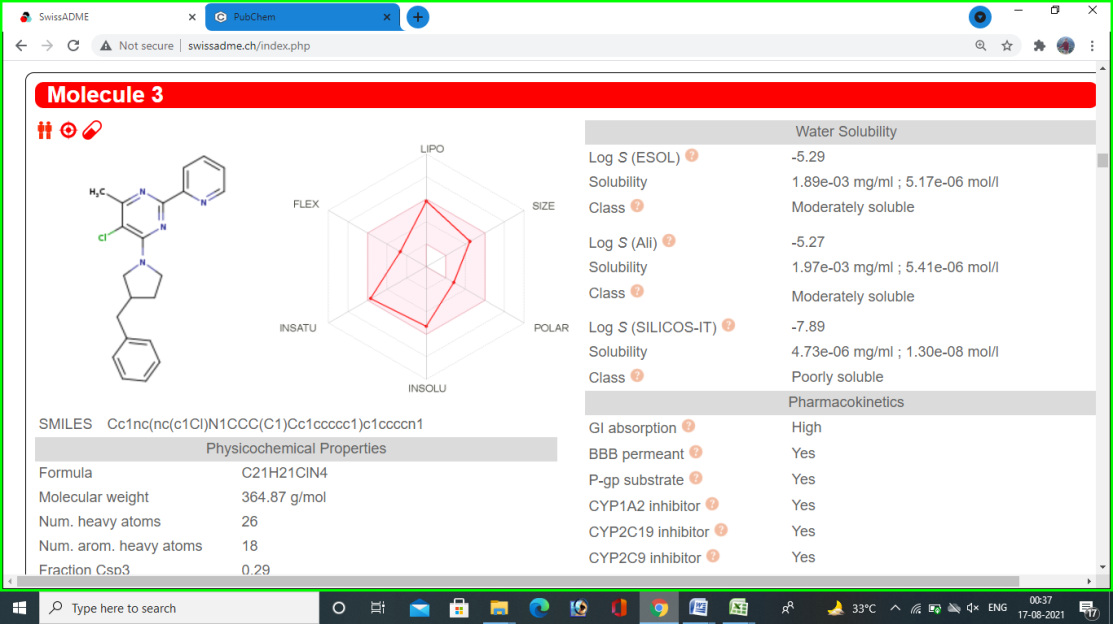 | 3.67 | -5.29 | -5.22 | Yes  0 violation | High | 0  alert |
|  | **MMV688991** | 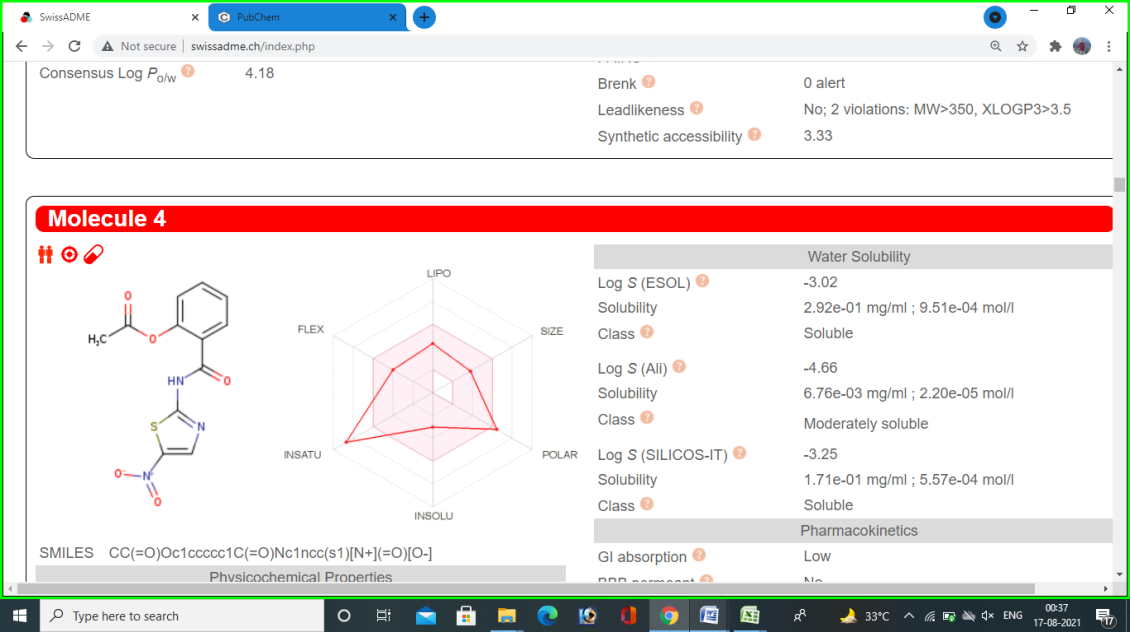 | 0.90 | -3.02 | -6.73 | Yes  0 violation | Low | 0  alert |
|  | **MMV687172** | 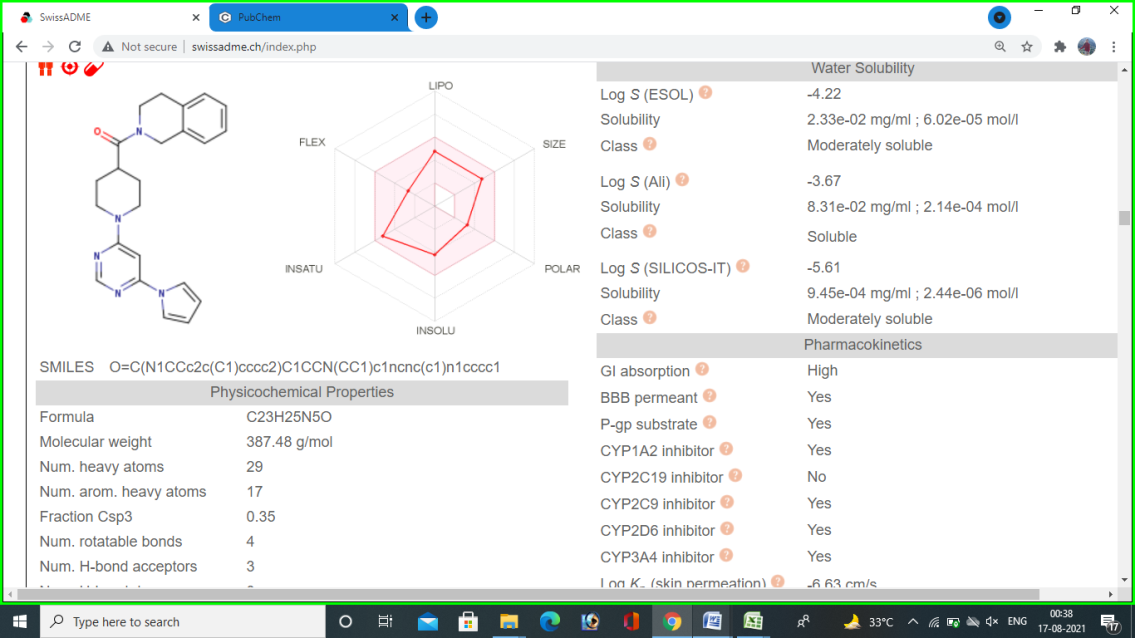 | 3.52 | -4.22 | -6.63 | Yes  0 violation | High | 0  alert |
|  | **MMV688180** | 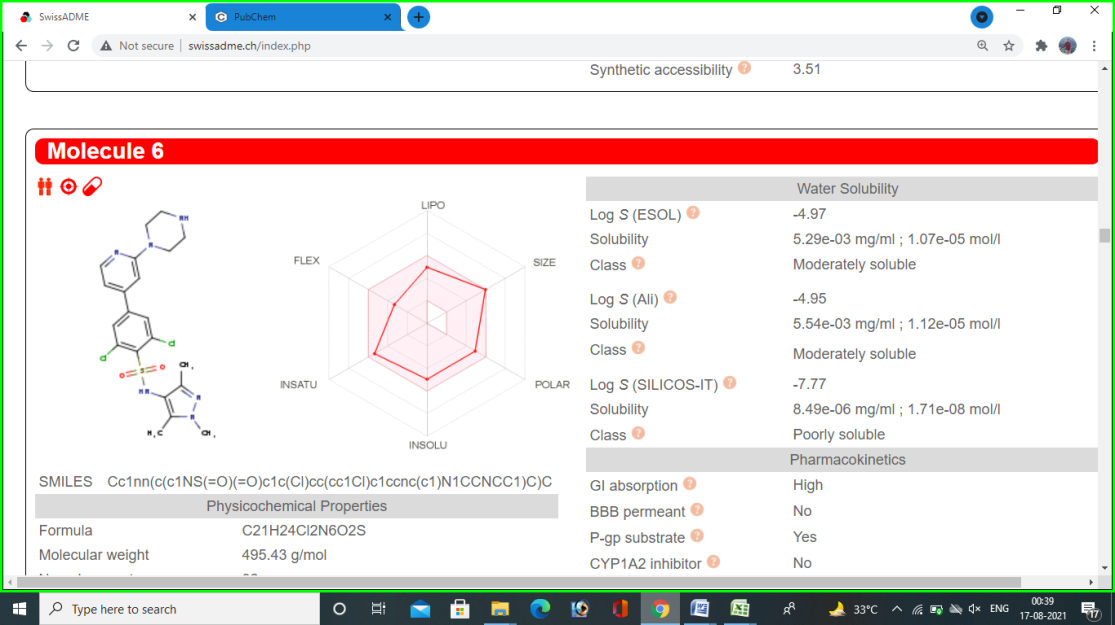 | 2.73 | -4.97 | -7.07 | Yes  0 violation | High | 0  alert |
|  | **MMV688552** | 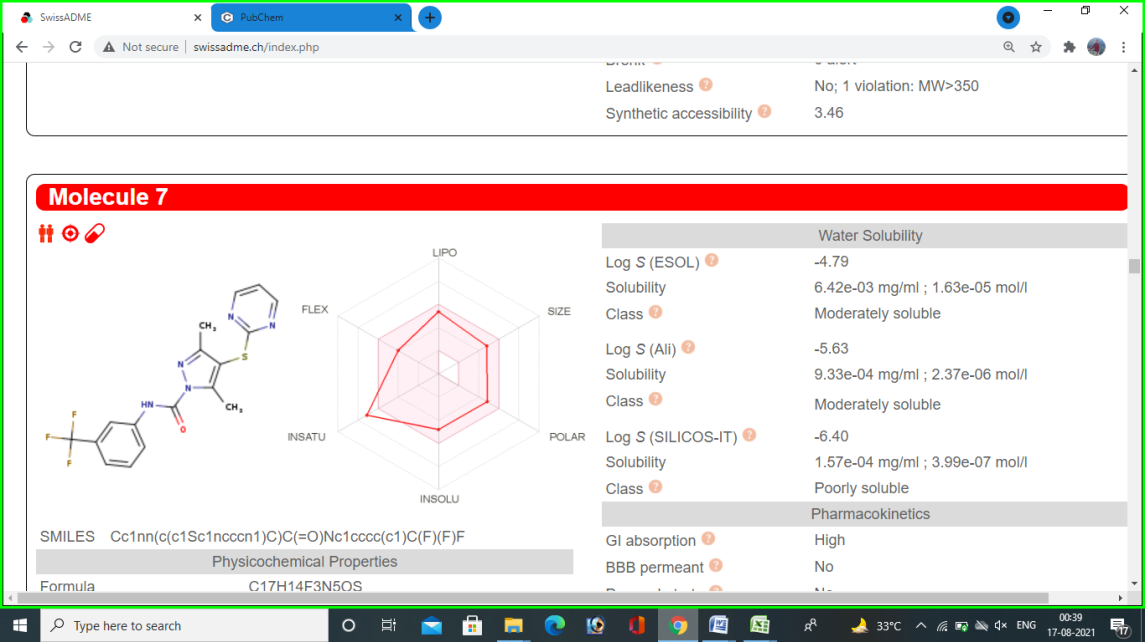 | 3.41 | -4.79 | -5.95 | Yes  0 violation | High | 0  alert |
|  | **MMV020136** | 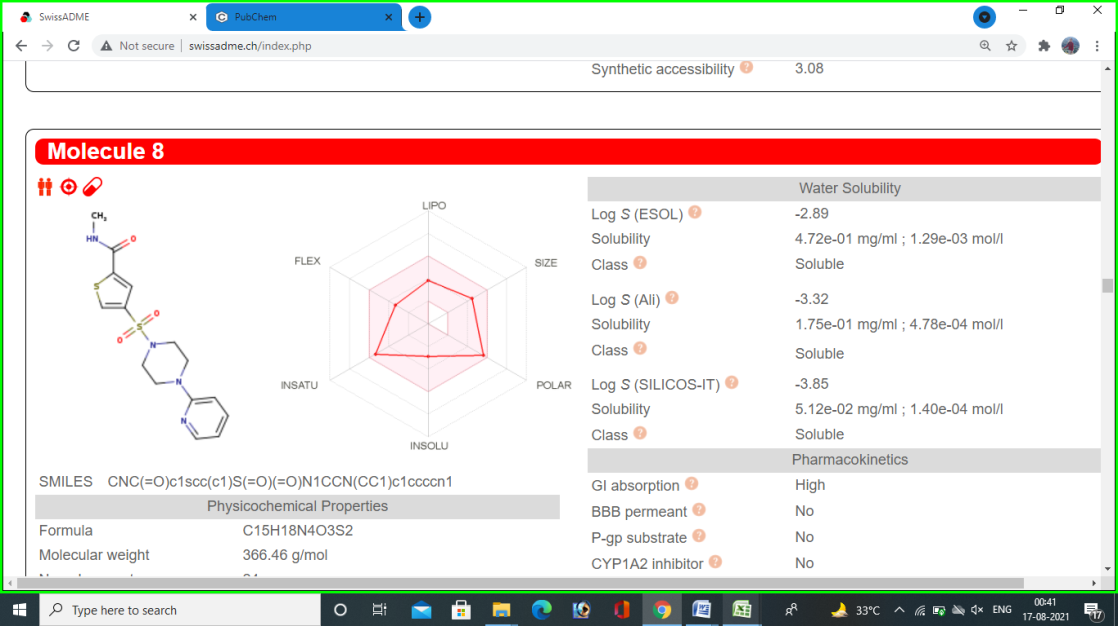 | 2.41 | -2.89 | -7.67 | Yes  0 violation | High | 0  alert |
|  | **MMV688407** | 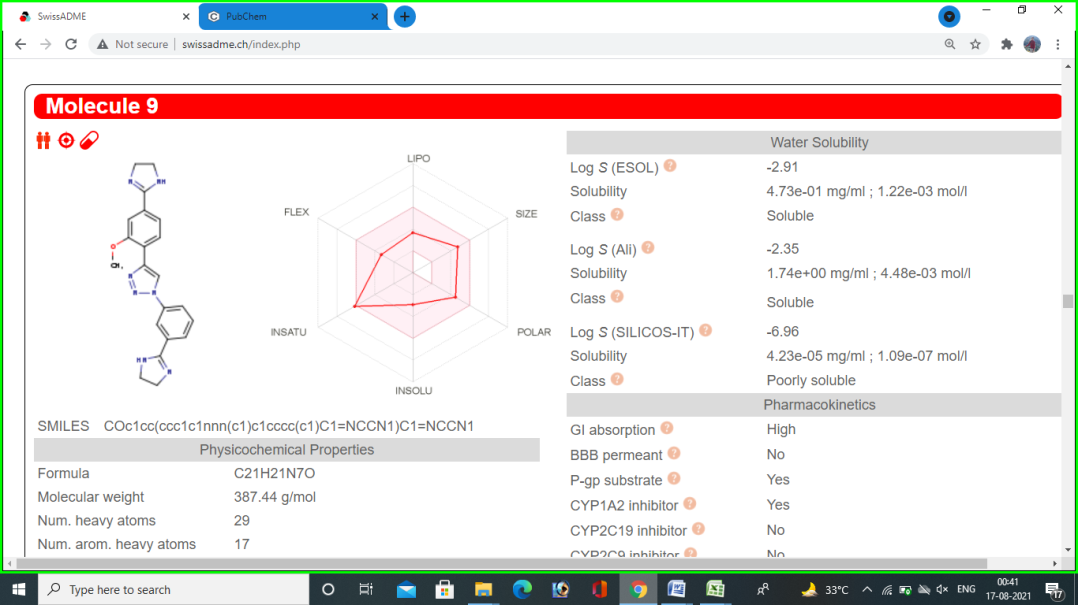 | 2.96 | -2.91 | -8.02 | Yes  0 violation | High | 0  alert |
|  | **MMV688262** | 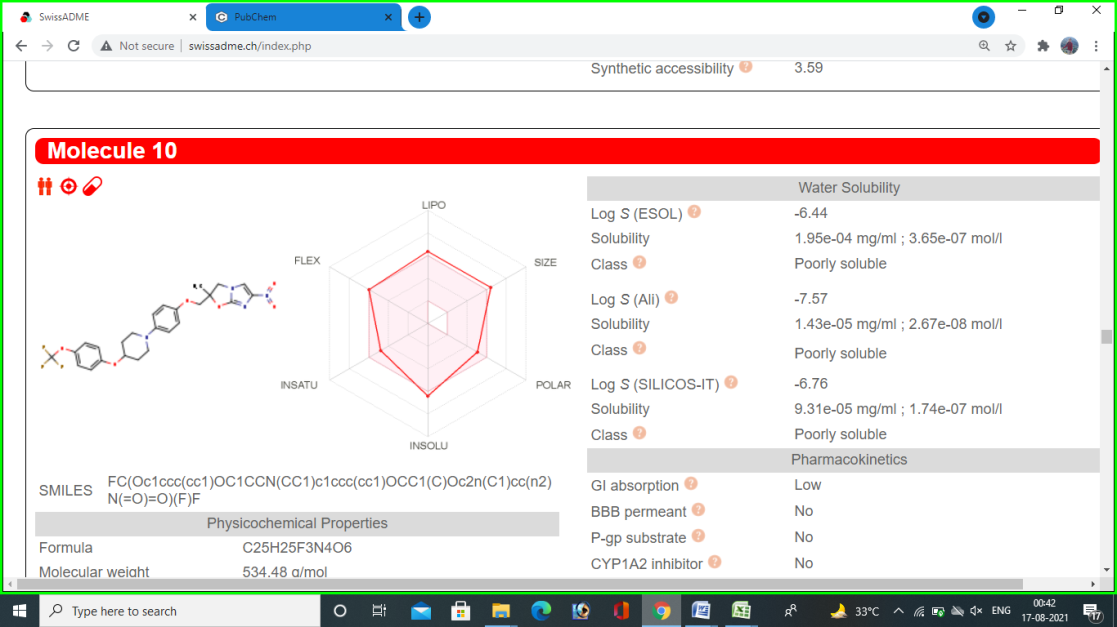 | 3.81 | -6.44 | -5.56 | No, 1 violation | Low | 1  alert |
|  | **MMV676597** | 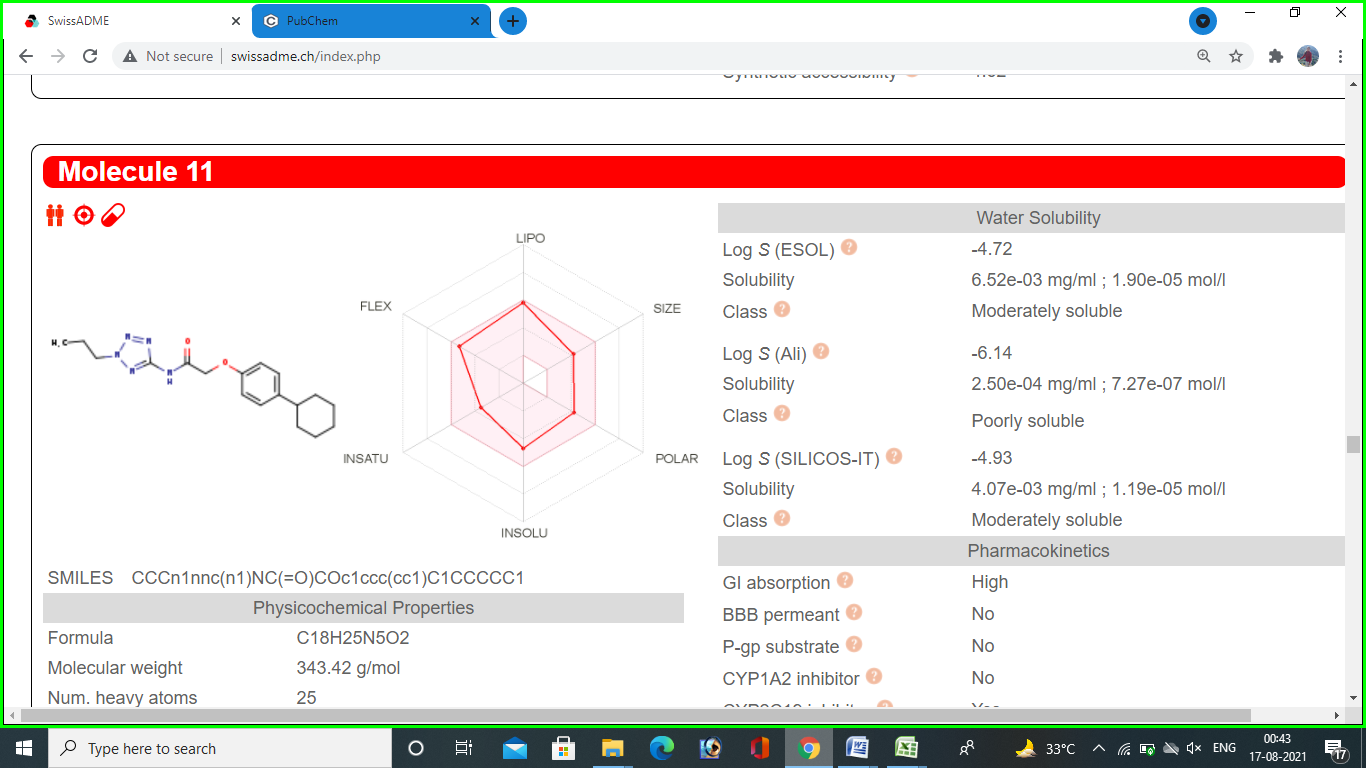 | 2.97 | -4.72 | -5.06 | Yes  0 violation | High | 0  alert |
|  | **MMV085499** | 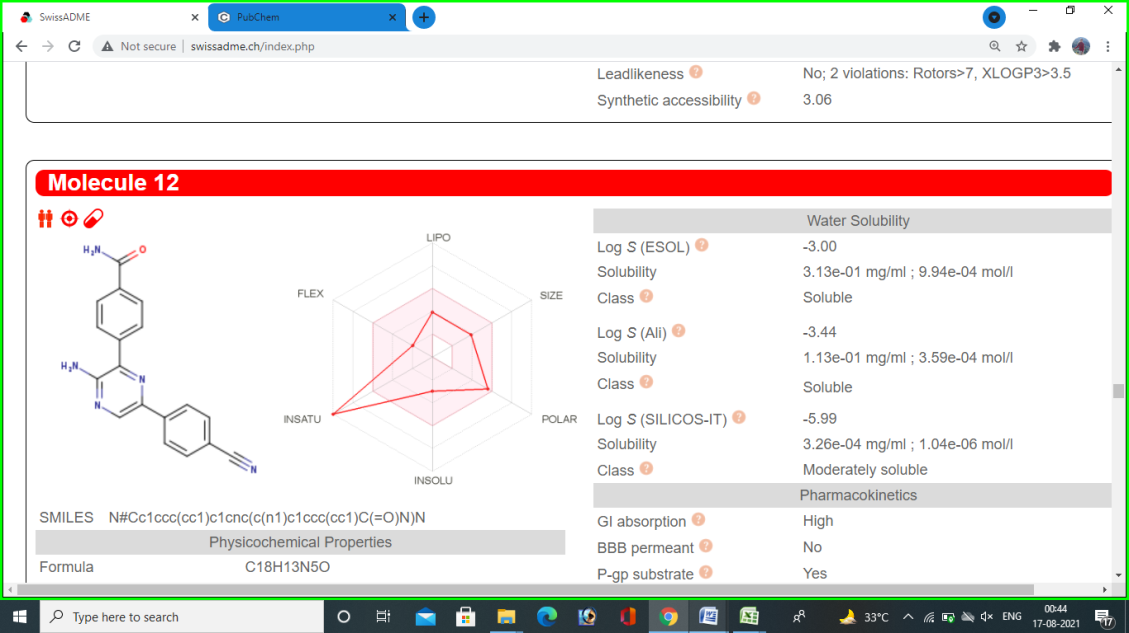 | 1.76 | -3.00 | -7.27 | Yes  0 violation | High | 0  alert |
|  | **MMV688474** | 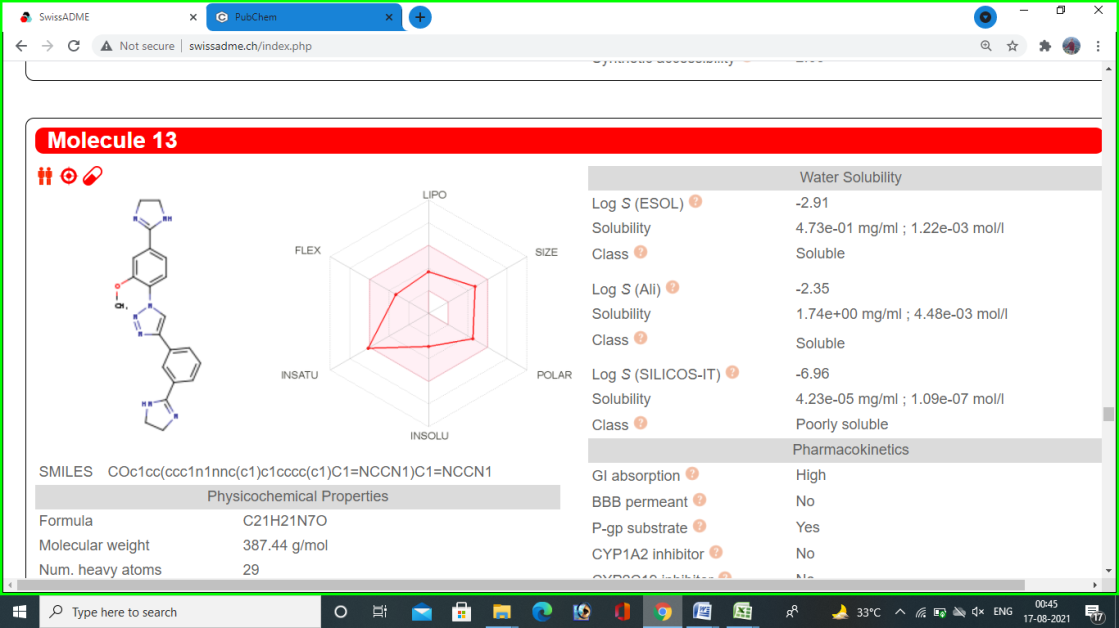 | 2.74 | -2.91 | -8.02 | Yes  0 violation | High | 0  alert |
|  | **MMV1029203** | 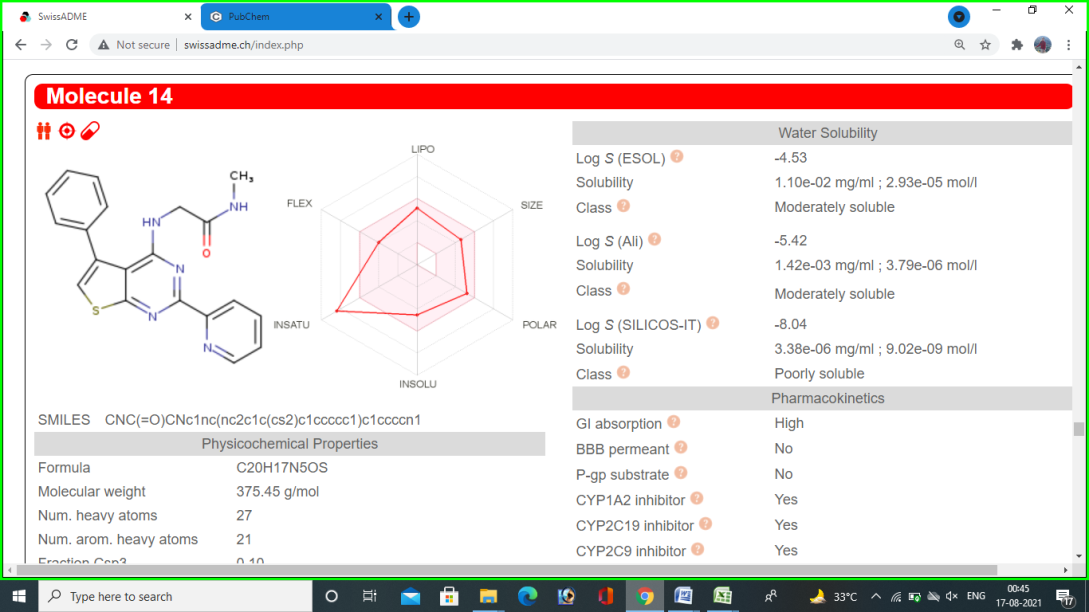 | 3.22 | -4.53 | -6.13 | Yes  0 violation | High | 0  alert |
|  | **MMV024406** | 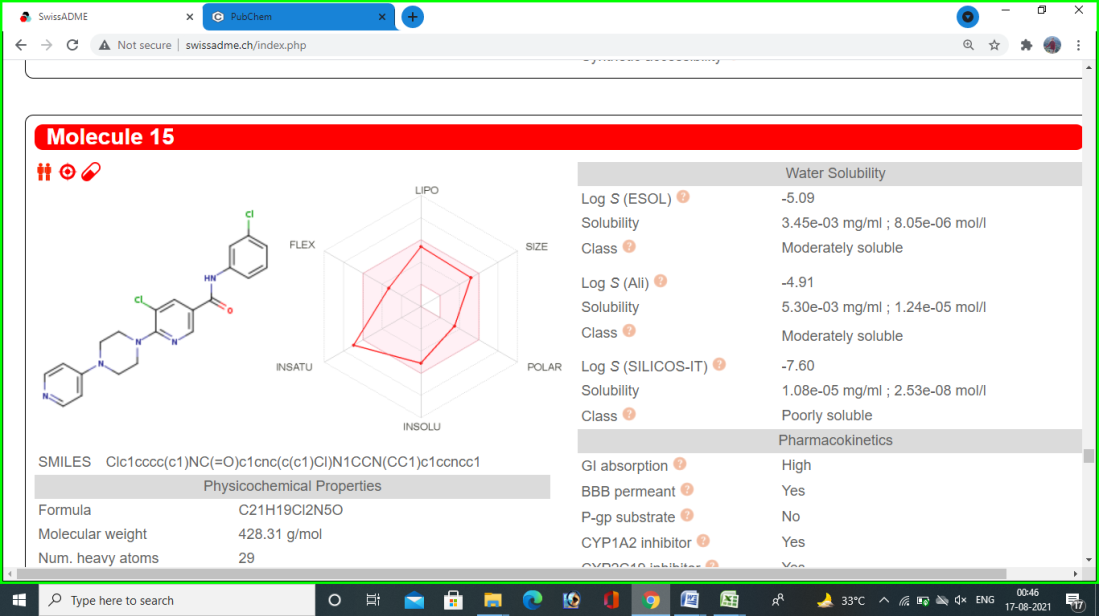 | 2.89 | -5.09 | -6.13 | Yes  0 violation | High | 0  alert |
|  | **MMV676509** | 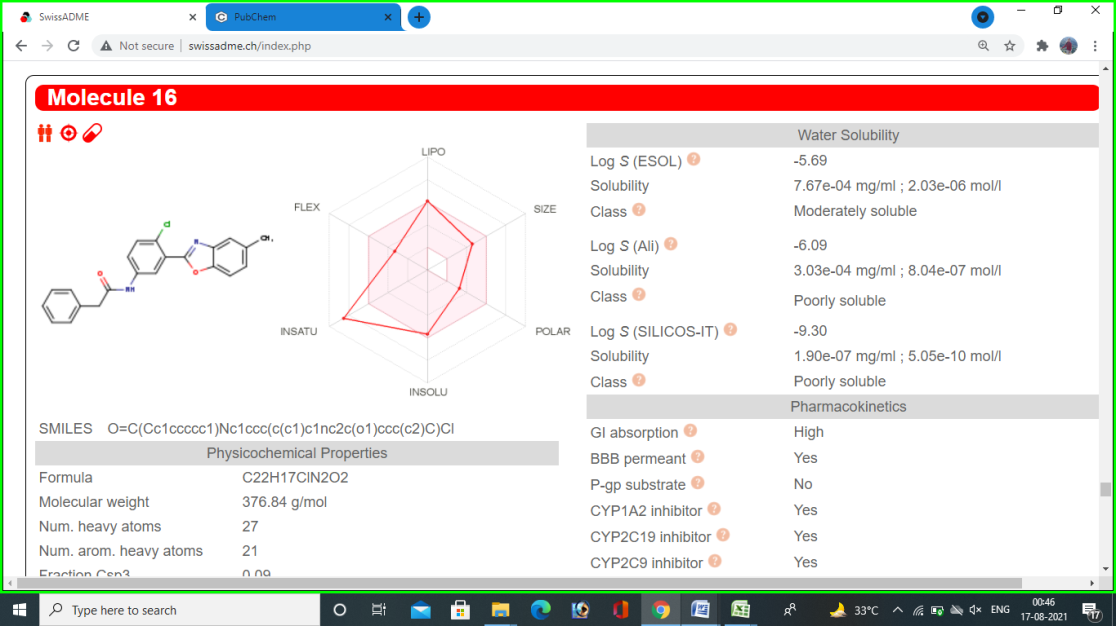 | 3.42 | -5.69 | -4.91 | Yes  0 violation | High | 0  alert |
|  | **MMV024397** | 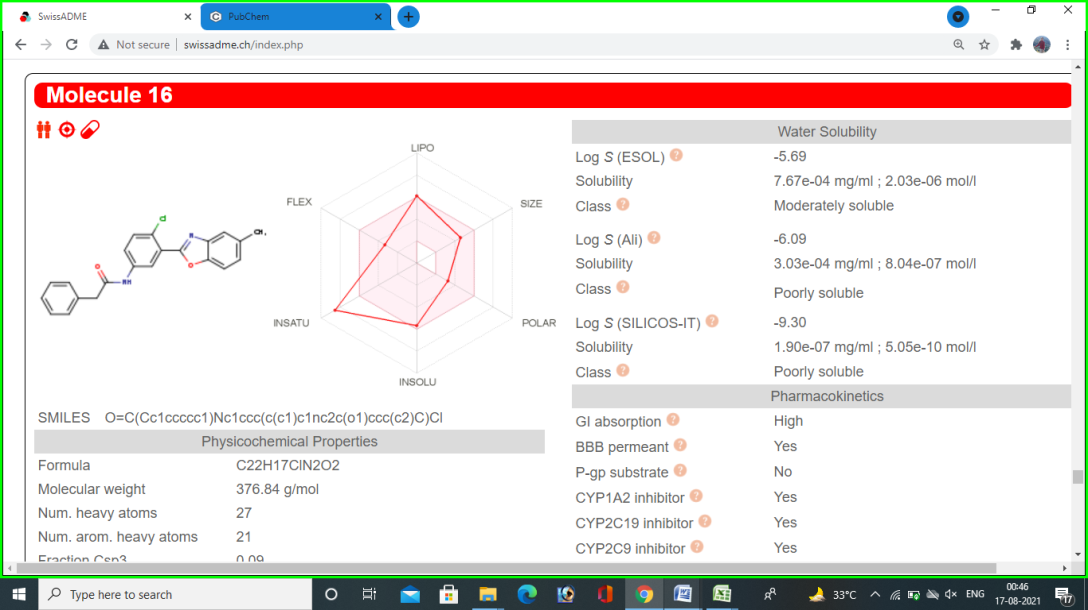 | 3.10 | -4.30 | -5.62 | Yes  0 violation | High | 0  alert |
|  | **MMV676555** | 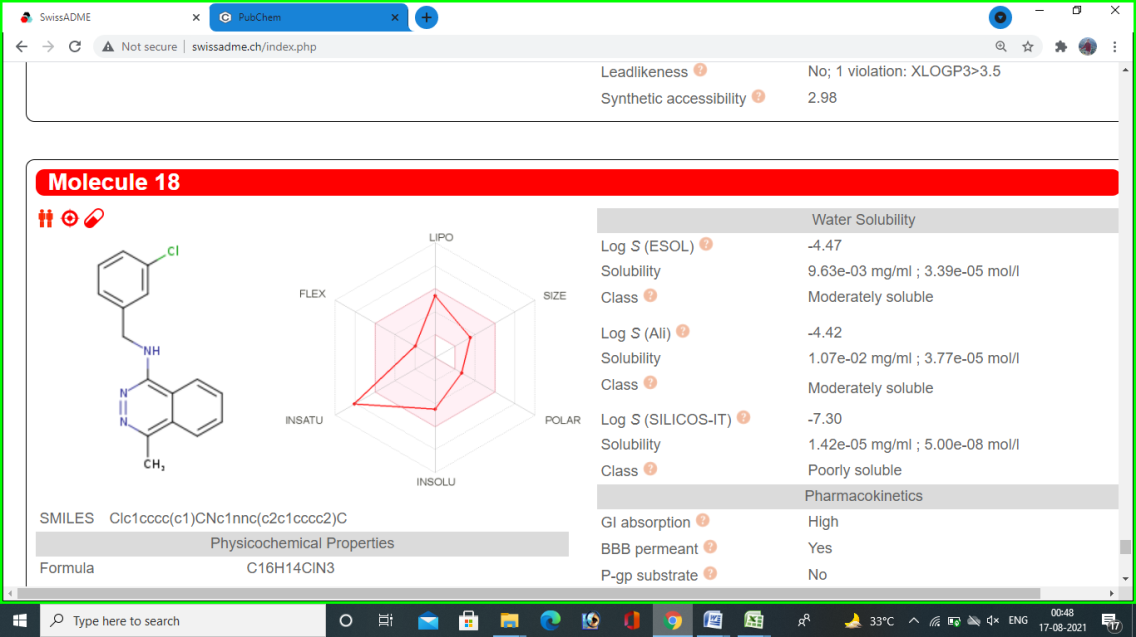 | 2.99 | -4.47 | -5.24 | Yes  0 violation | High | 0  alert |
|  | **MMV675998** | 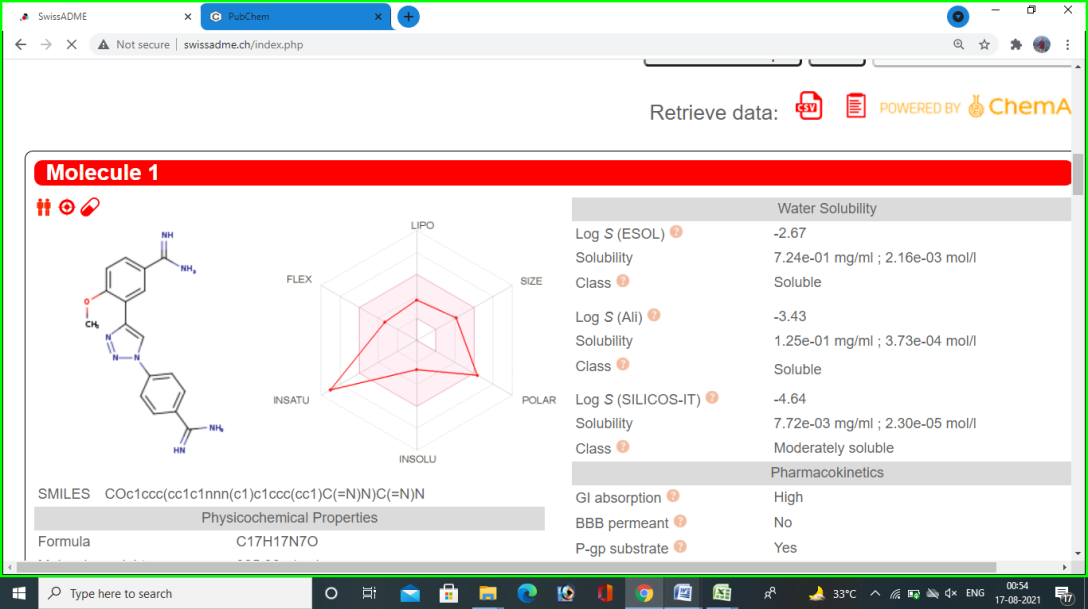 | 1.34 | -2.67 | -7.70 | Yes  0 violation | High | 0  alert |
|  | **MMV560185** | 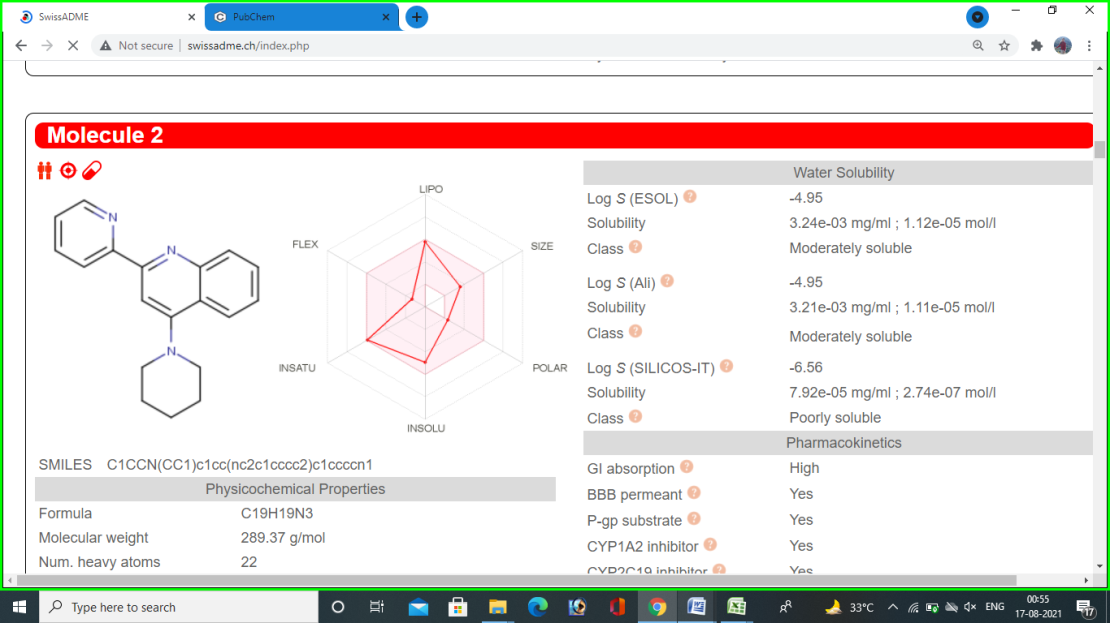 | 3.35 | -4.95 | -4.78 | Yes  0 violation | High | 0  alert |
|  | **MMV688471** | 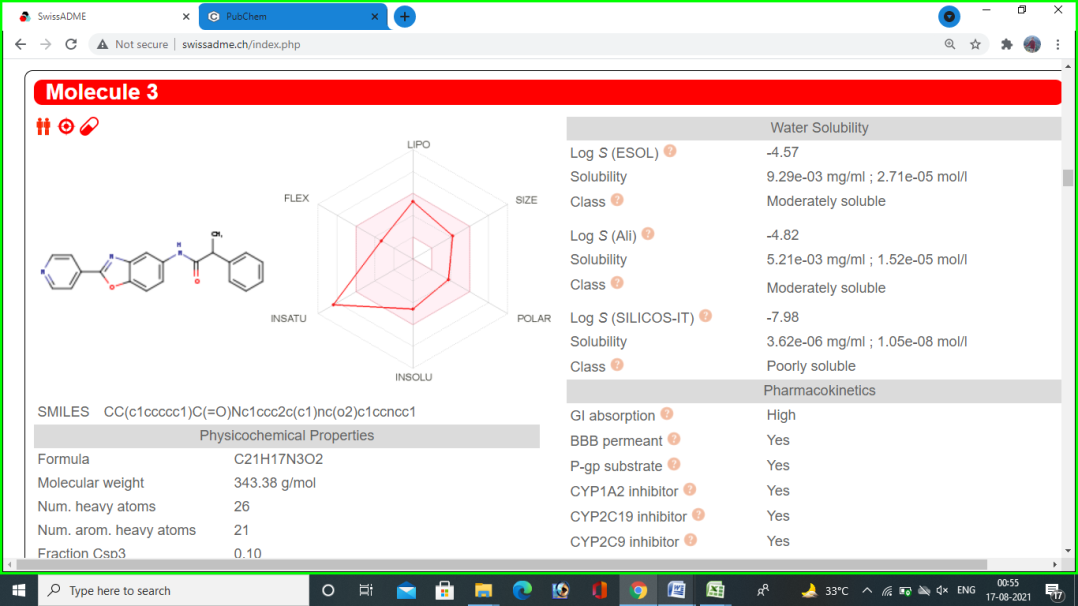 | 2.83 | -4.57 | -5.77 | Yes  0 violation | High | 0  alert |
|  | **MMV090930** | 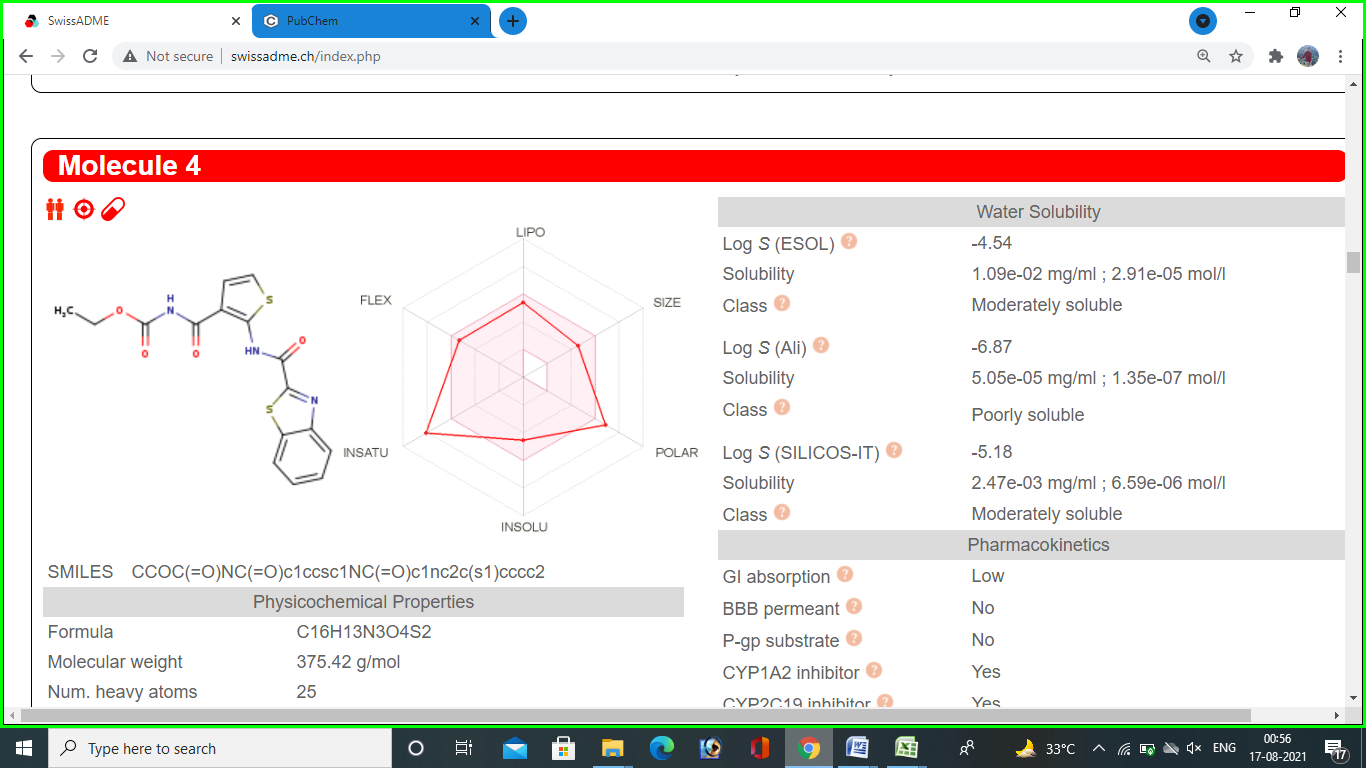 | 1.81 | -4.54 | -5.79 | Yes  0 violation | Low | 0  alert |
|  | **MMV687189** | 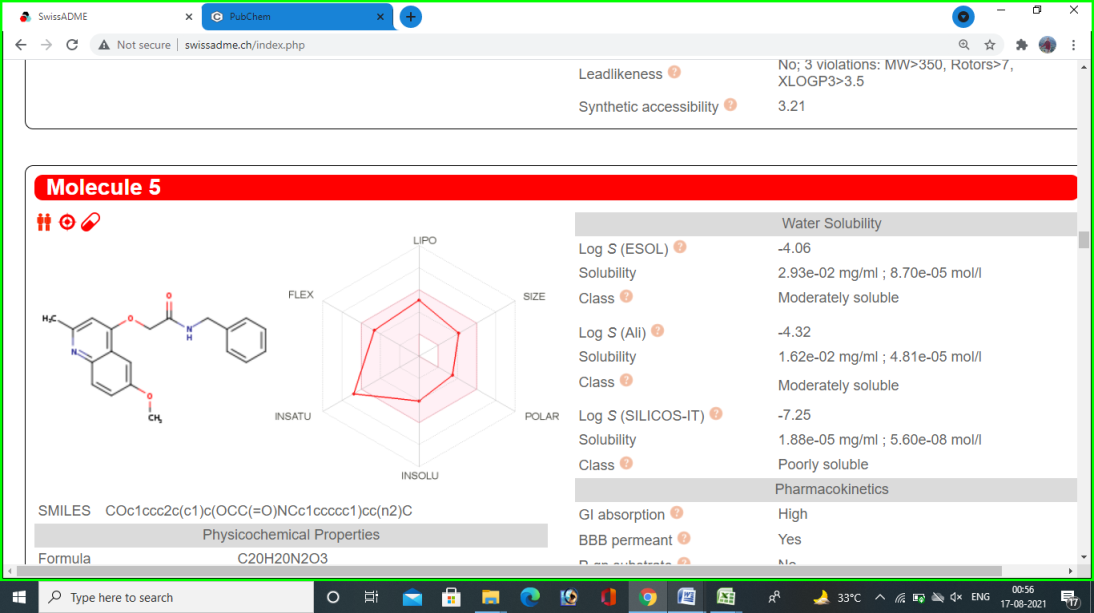 | 2.98 | -4.06 | -5.96 | Yes  0 violation | High | 0  alert |
|  | **MMV688362** | 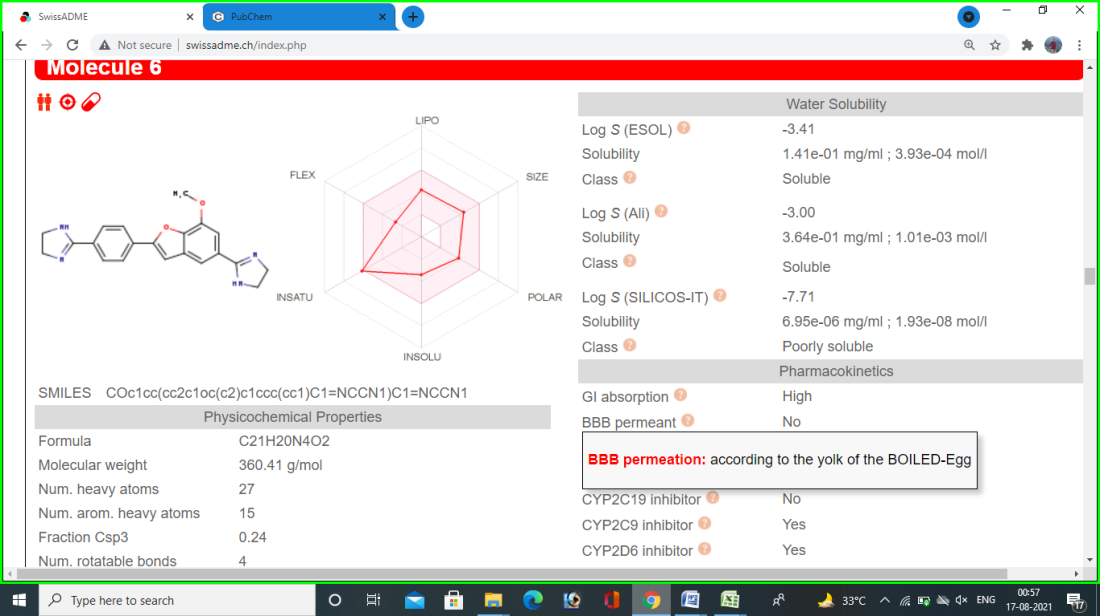 | 3.01 | -3.41 | -7.16 | Yes  0 violation | High | 0  alert |
|  | **MMV019087** | 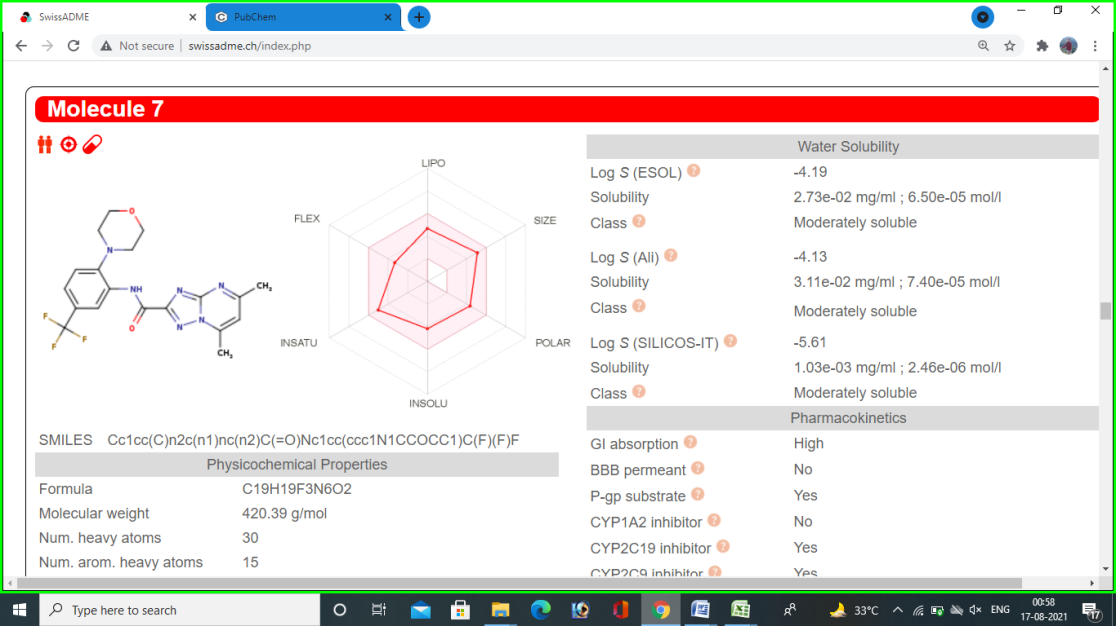 | 2.79 | -4.19 | -6.95 | Yes  0 violation | High | 0  alert |
|  | **MMV689061** | 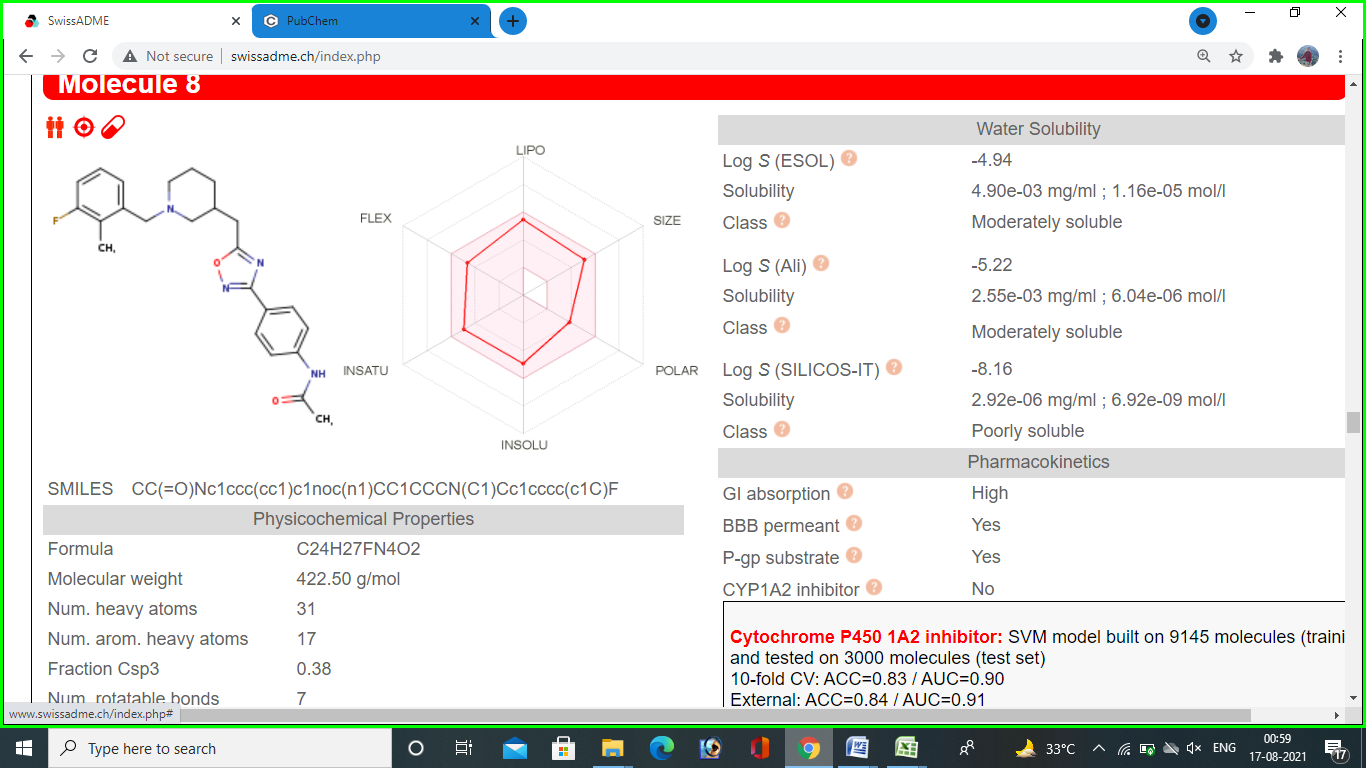 | 3.87 | -4.94 | -6.02 | Yes  0 violation | High | 0  alert |
|  | **MMV689029** | 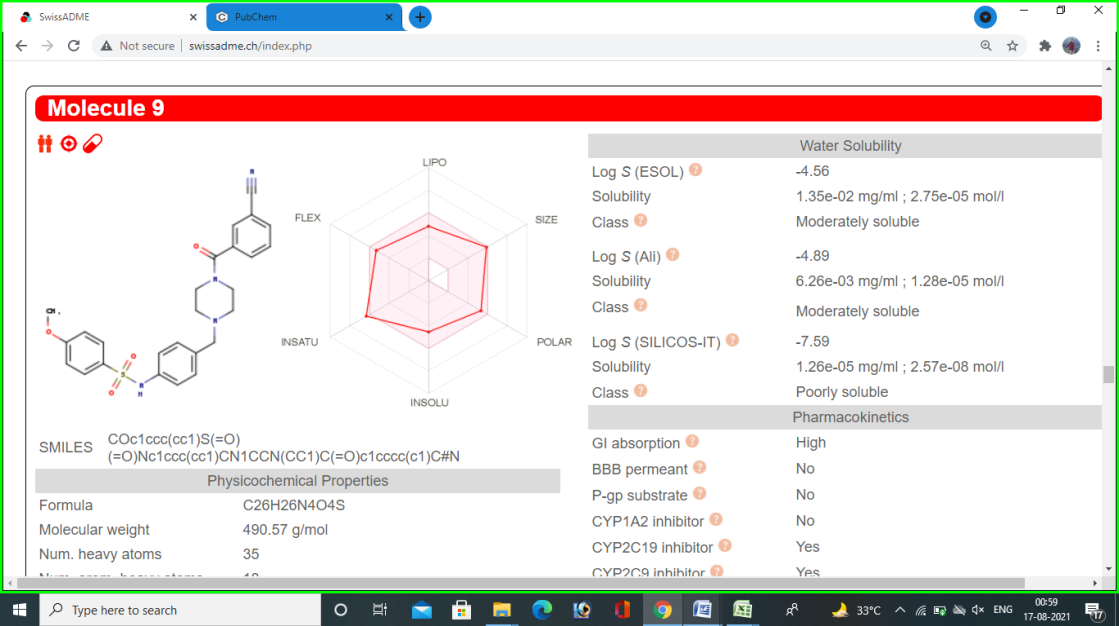 | 3.32 | -4.56 | -7.23 | Yes  0 violation | High | 0  alert |
|  | **MMV688768** | 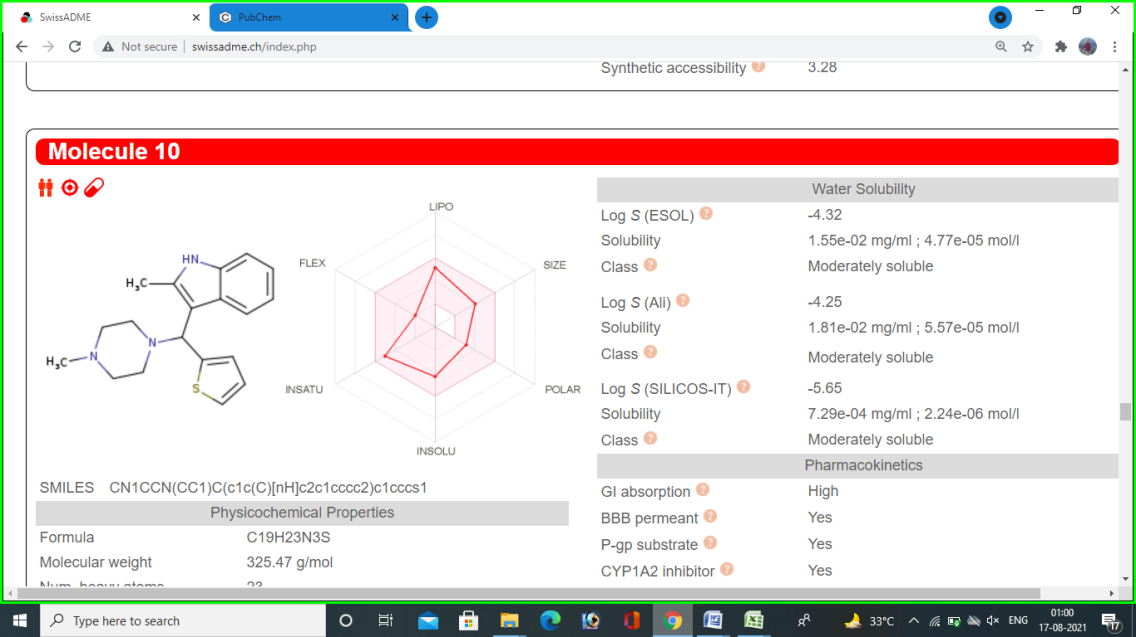 | 3.18 | -4.32 | -5.79 | Yes  0 violation | High | 1 alert |
|  | **MMV688762** | 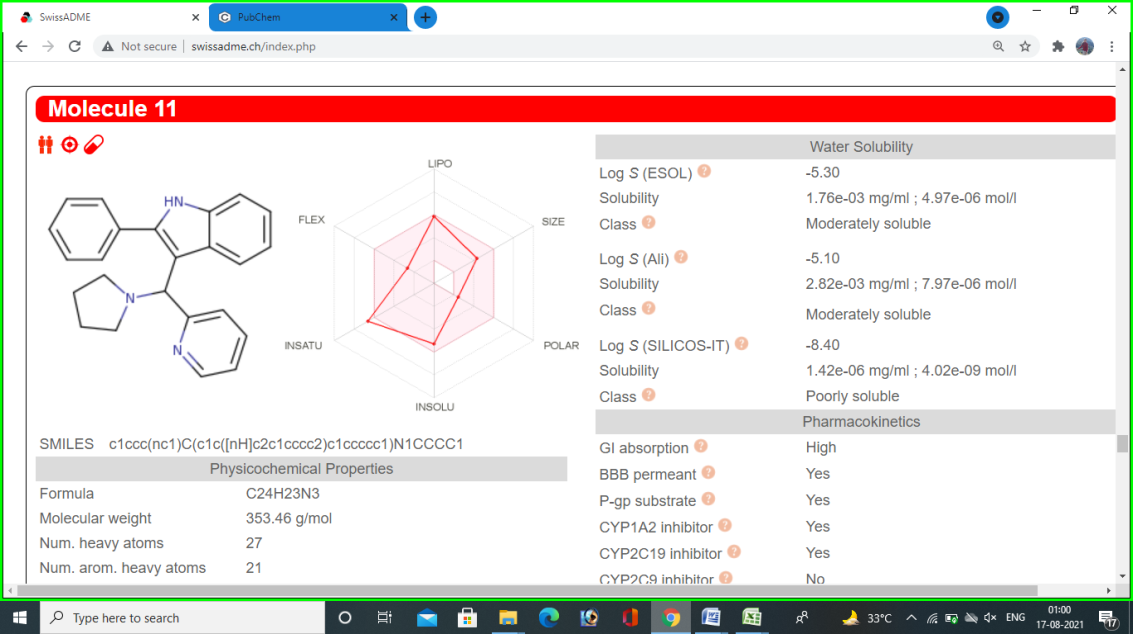 | 3.10 | -5.30 | -5.12 | Yes  0 violation | High | 0  alert |
|  | **MMV030734** | 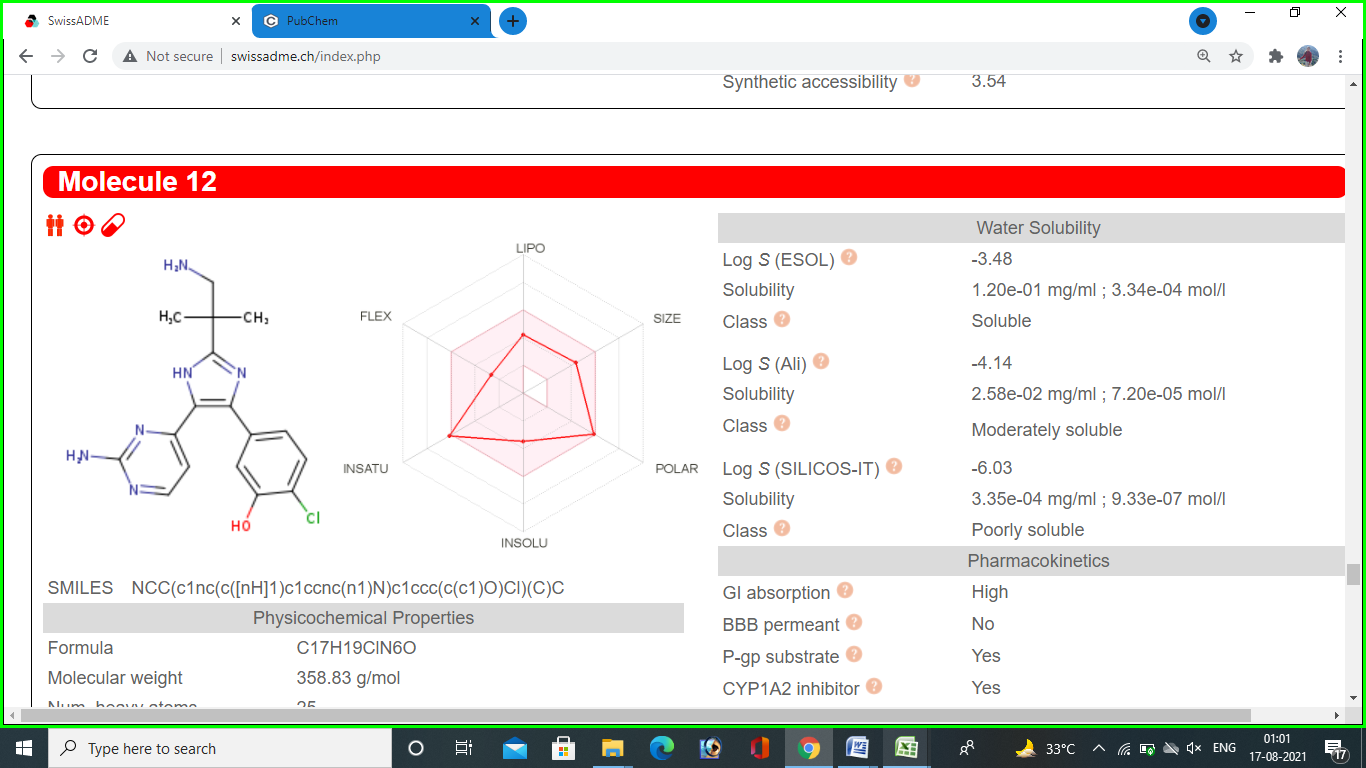 | 1.83 | -3.48 | -7.17 | Yes  0 violation | High | 0  alert |
|  | **MMV001493** | 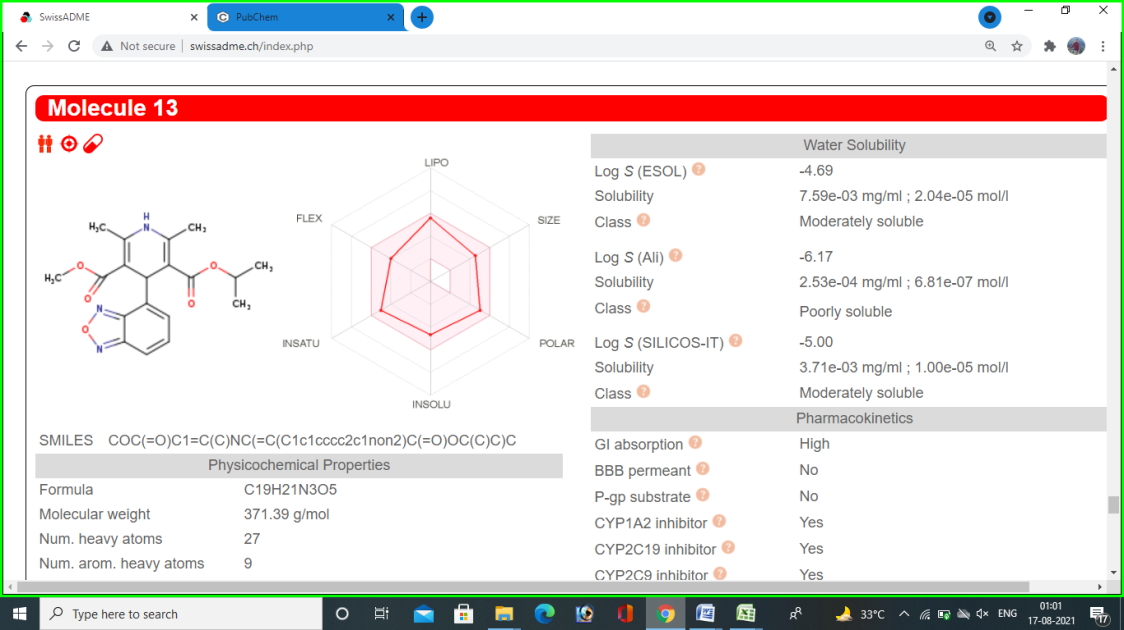 | 3.46 | -4.69 | -5.53 | Yes  0 violation | High | 0  alert |
|  | **MMV687729** | 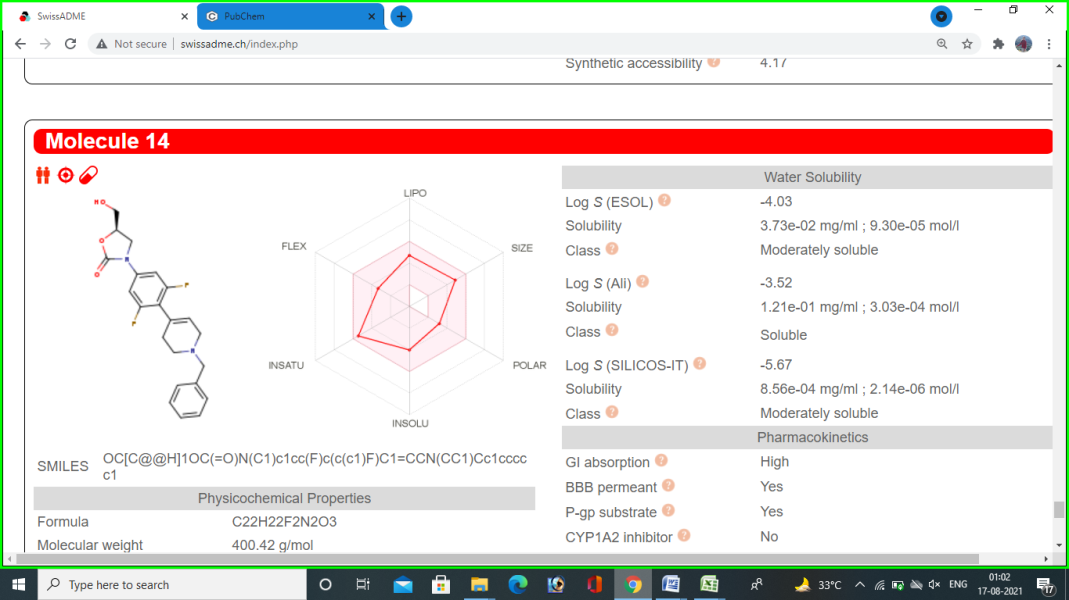 | 3.46 | -4.03 | -6.79 | Yes  0 violation | High | 0  alert |
|  | **MMV687775** | 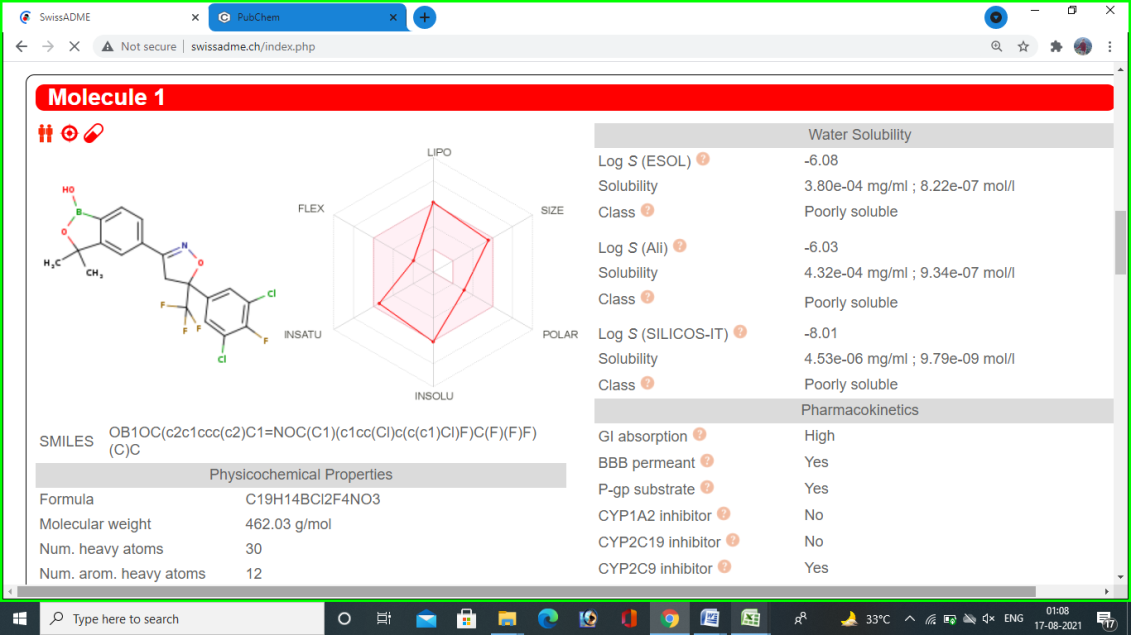 | 0.00 | -6.08 | -5.42 | Yes  0 violation | High | 0  alert |
|  | **MMV687794** | 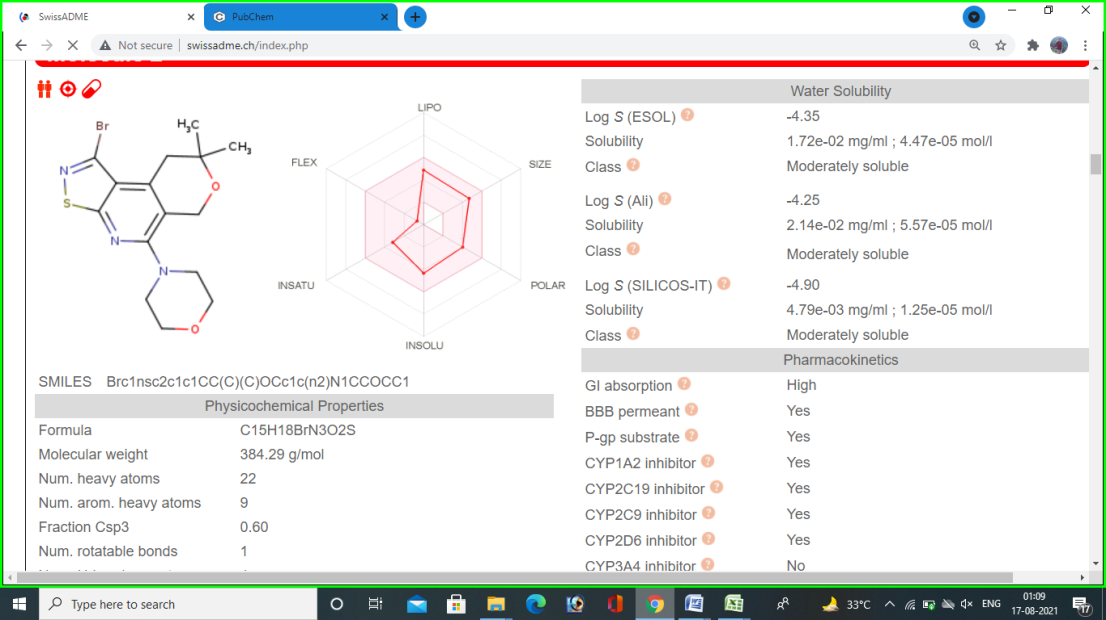 | 3.30 | -4.35 | -6.51 | Yes  0 violation | High | 0  alert |
|  | **MMV032967** | 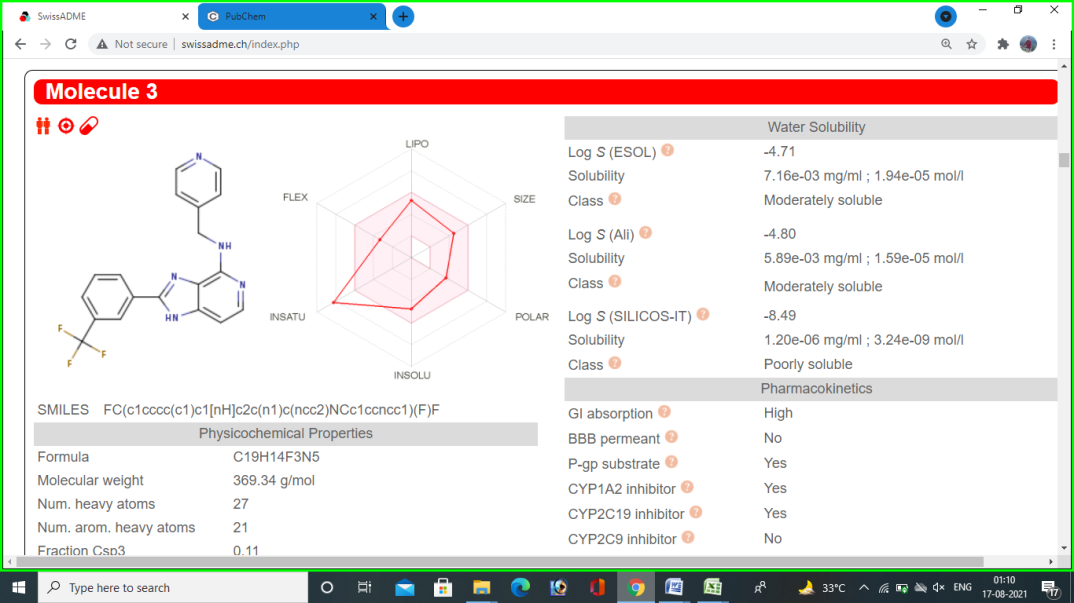 | 2.32 | -4.71 | -5.92 | Yes  0 violation | High | 0  alert |
|  | **MMV020165** | 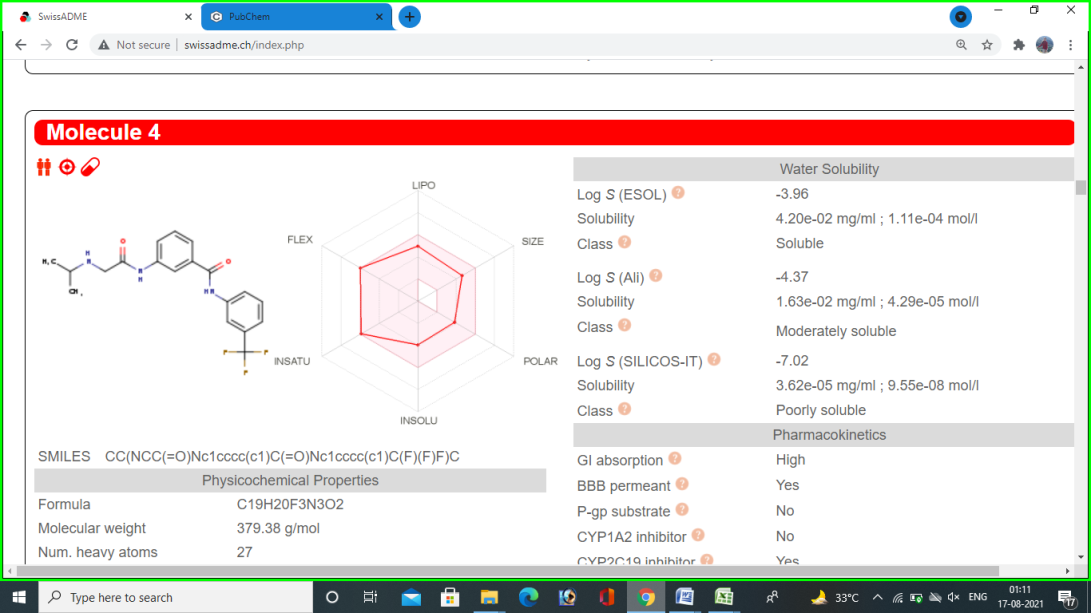 | 2.83 | -3.96 | -6.33 | Yes  0 violation | High | 0  alert |
|  | **MMV020512** | 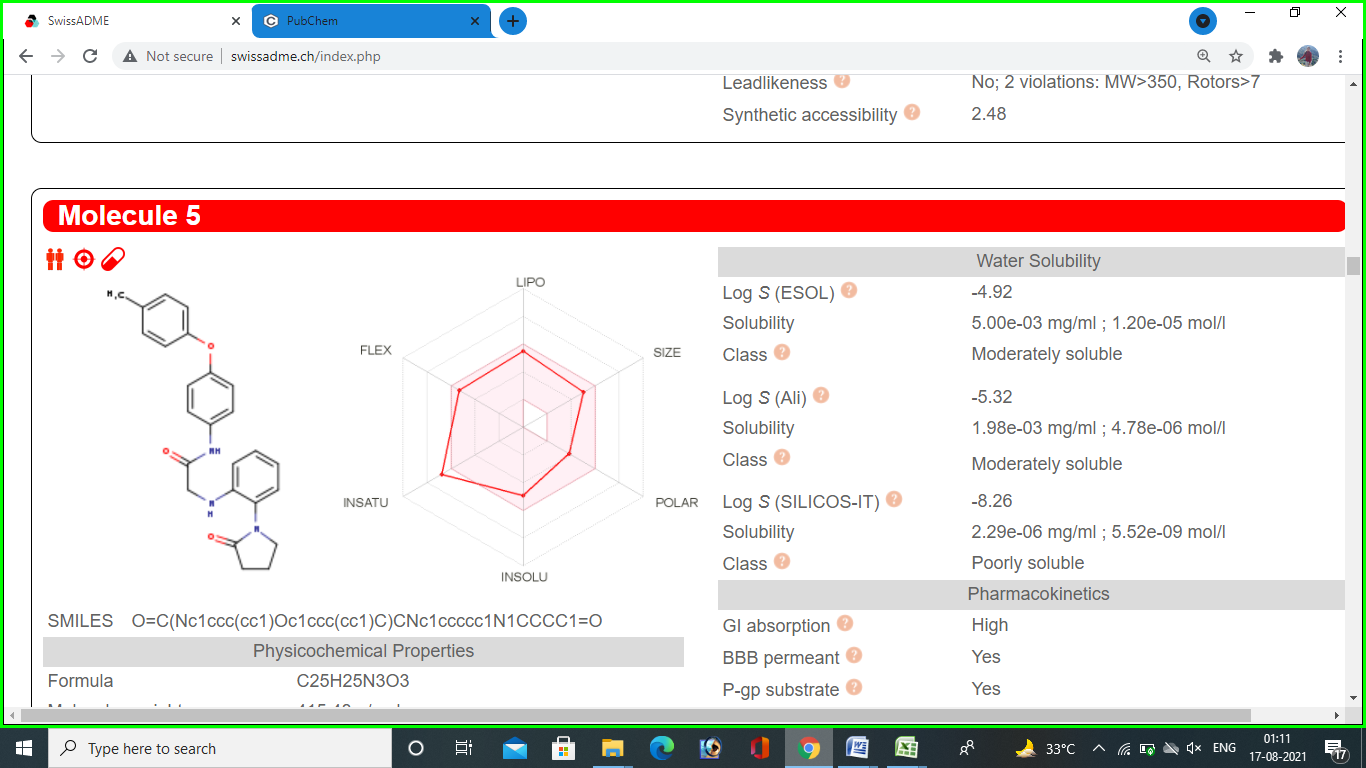 | 3.70 | -4.92 | -5.90 | Yes  0 violation | High | 0  alert |
|  | **MMV676602** | 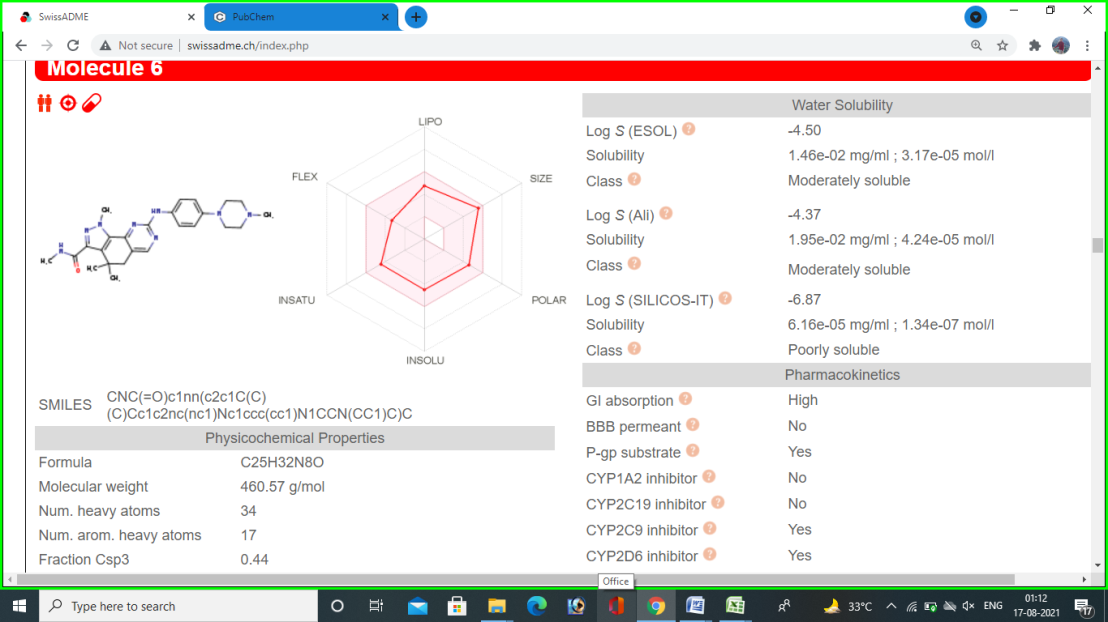 | 3.73 | -4.50 | -7.12 | Yes  0 violation | High | 1  alert |
|  | **MMV008439** | 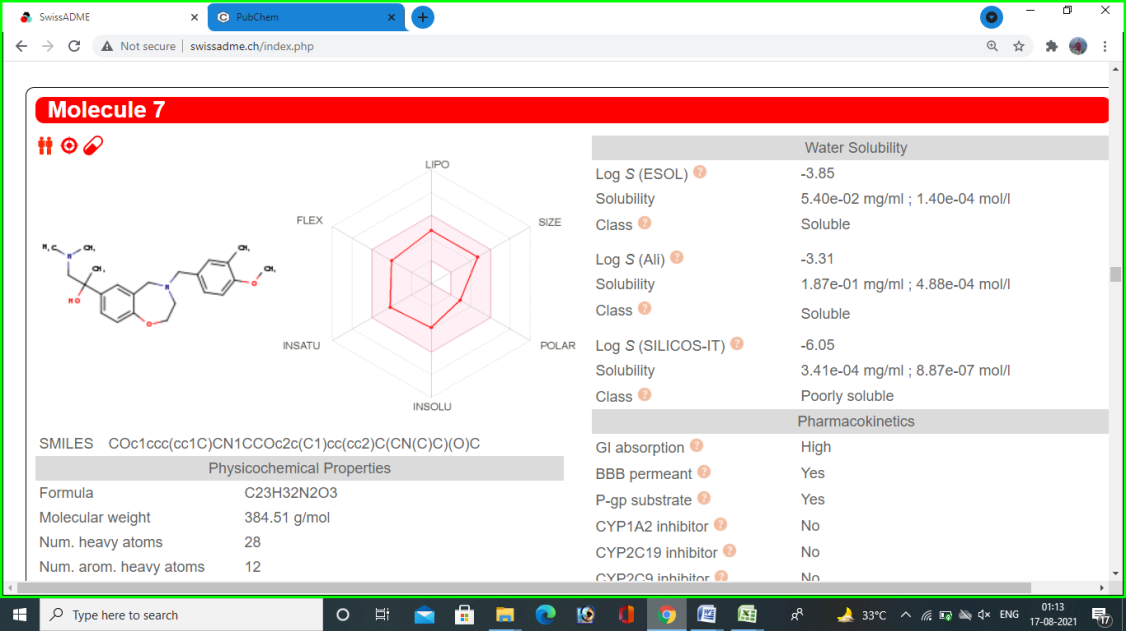 | 4.14 | -3.85 | -6.72 | Yes  0 violation | High | 0  alert |
|  | **MMV020982** | 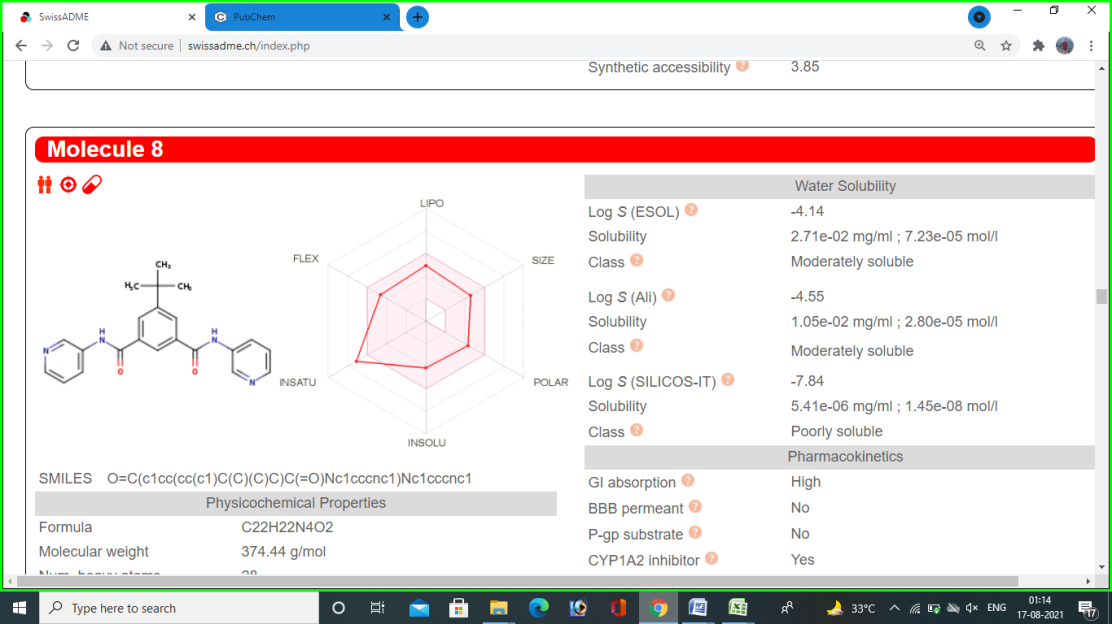 | 2.59 | -4.14 | -6.37 | Yes  0 violation | High | 0  alert |
|  | **MMV688921** | 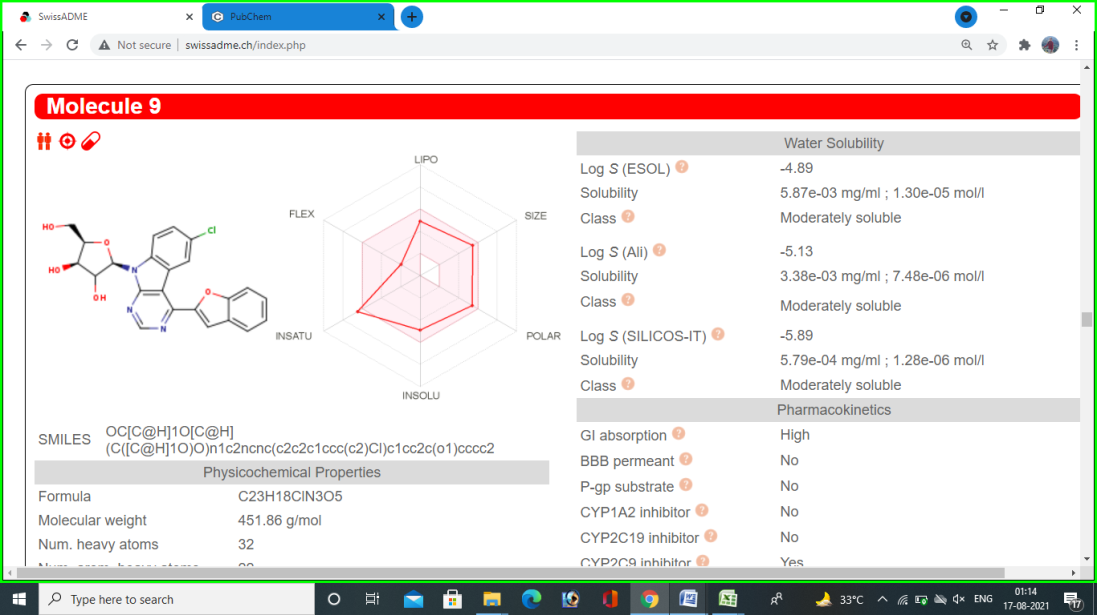 | 3.19 | -4.89 | -6.86 | Yes  0 violation | High | 0  alert |
|  | **MMV024829** | 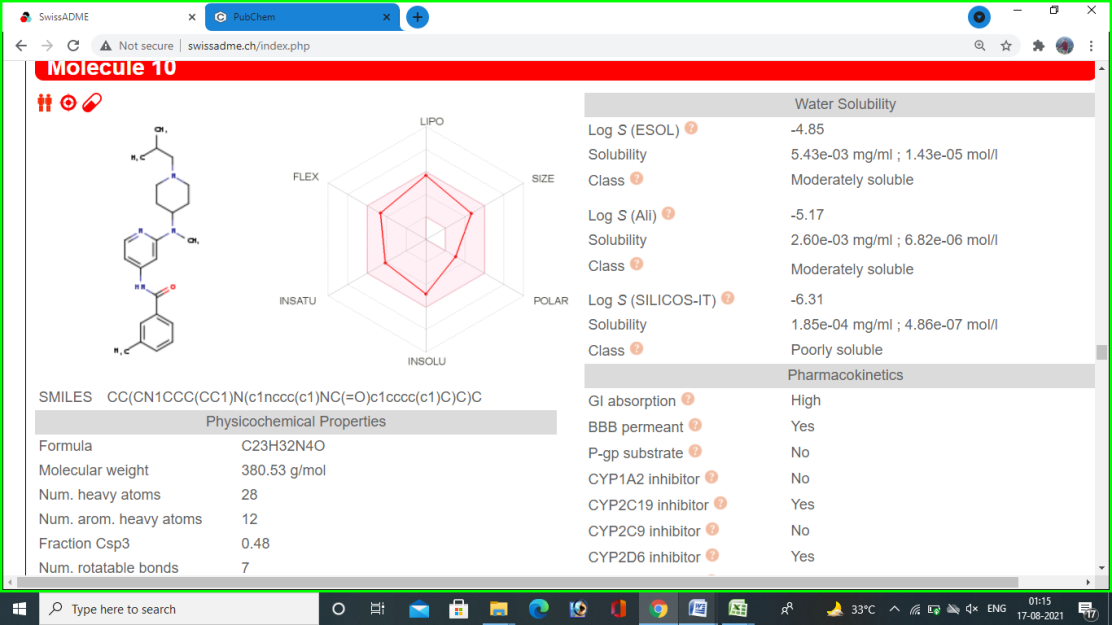 | 3.79 | -4.85 | -5.48 | Yes  0 violation | High | 0  alert |
|  | **MMV004168** | 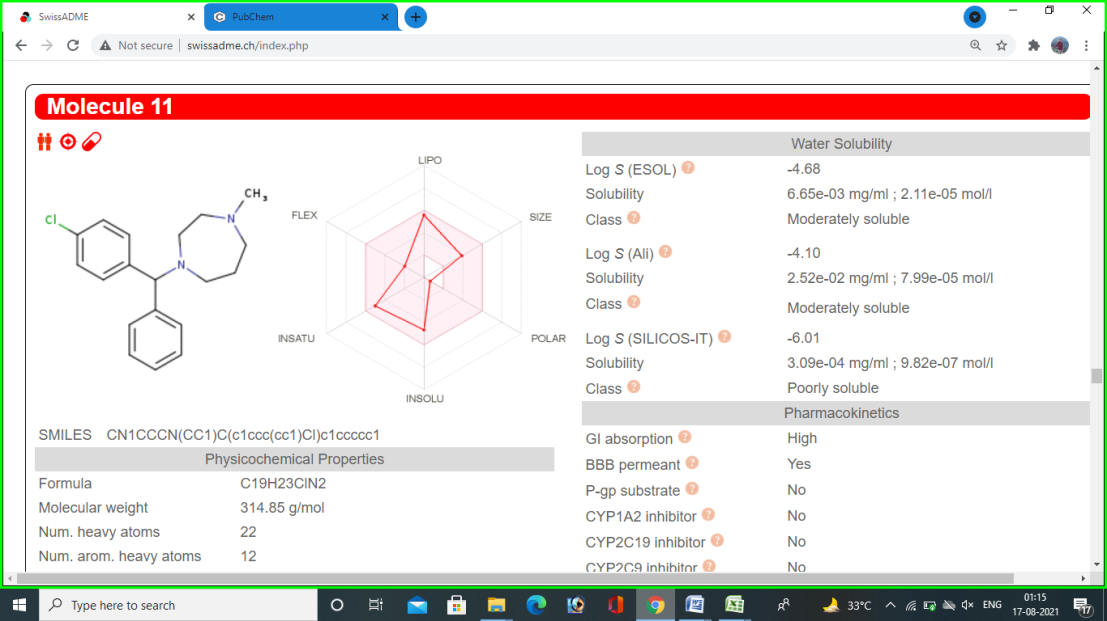 | 3.65 | -4.68 | -5.20 | Yes  0 violation | High | 0  alert |
|  | **MMV688466** | 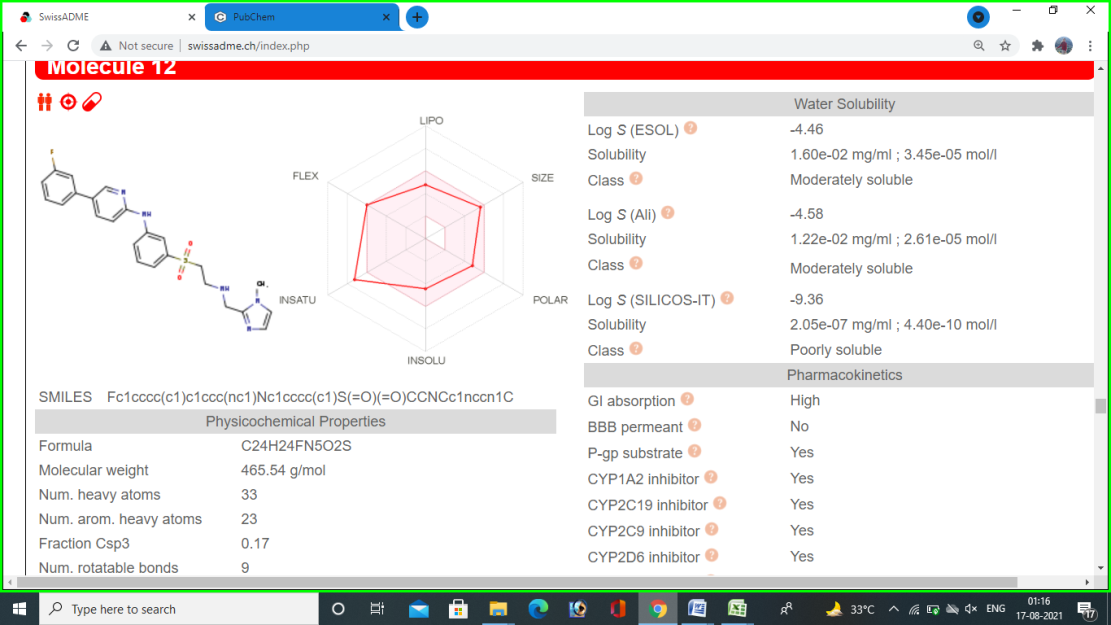 | 3.10 | -4.46 | -7.10 | Yes  0 violation | High | 0  alert |
|  | **MMV676478** | 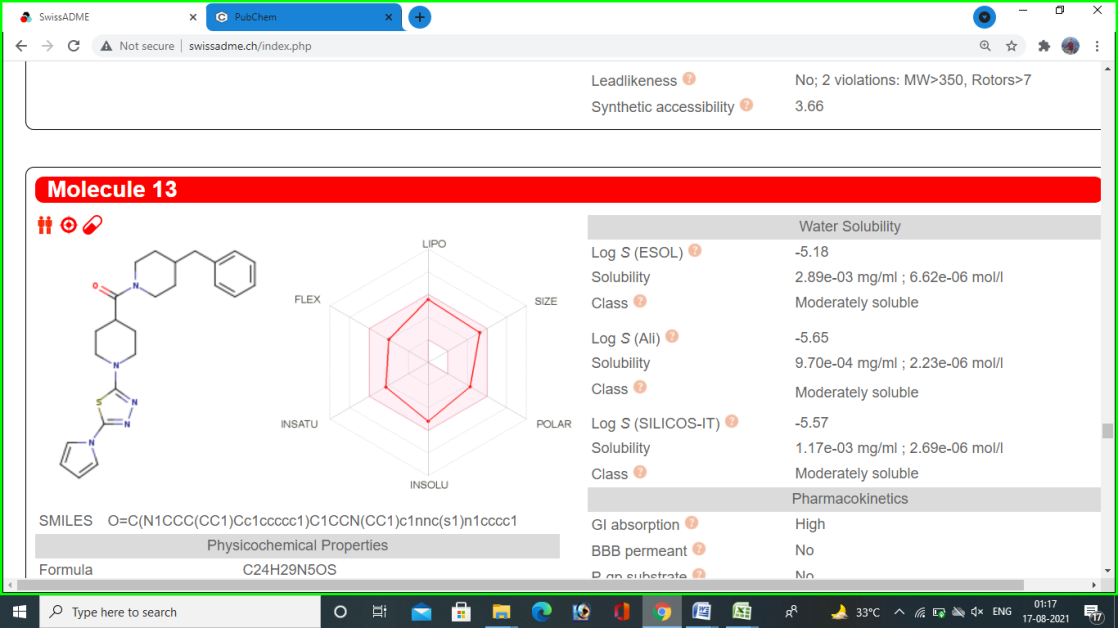 | 4.11 | -5.18 | -5.97 | Yes  0 violation | High | 0  alert |
|  | **MMV019189** | 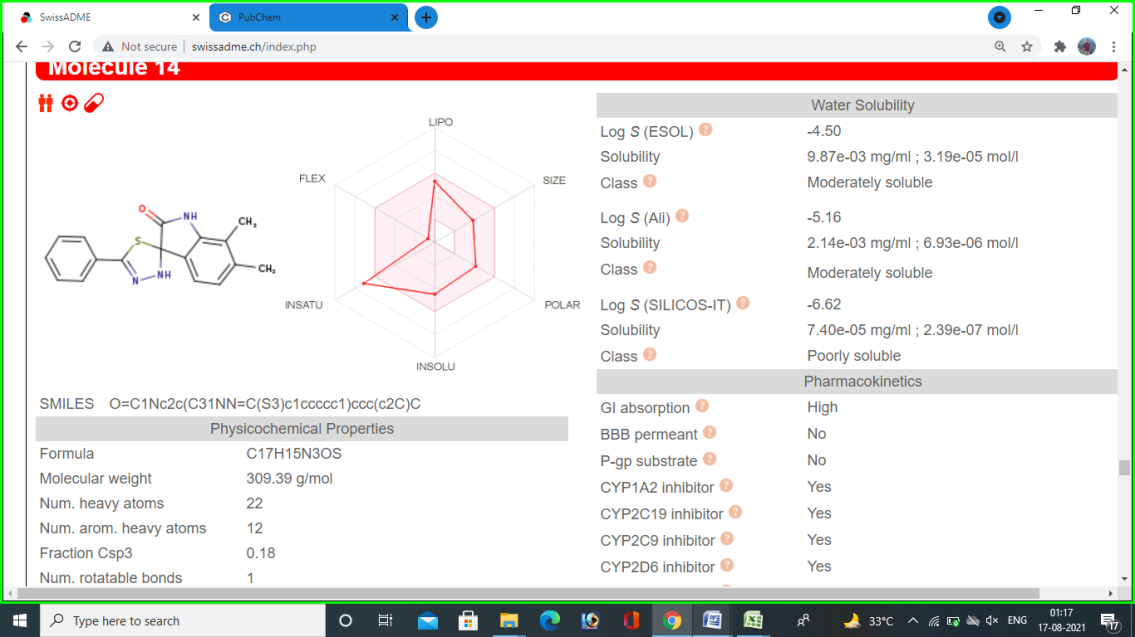 | 2.53 | -4.50 | -5.48 | Yes  0 violation | High | 1 alert |
|  | **MMV688271** | 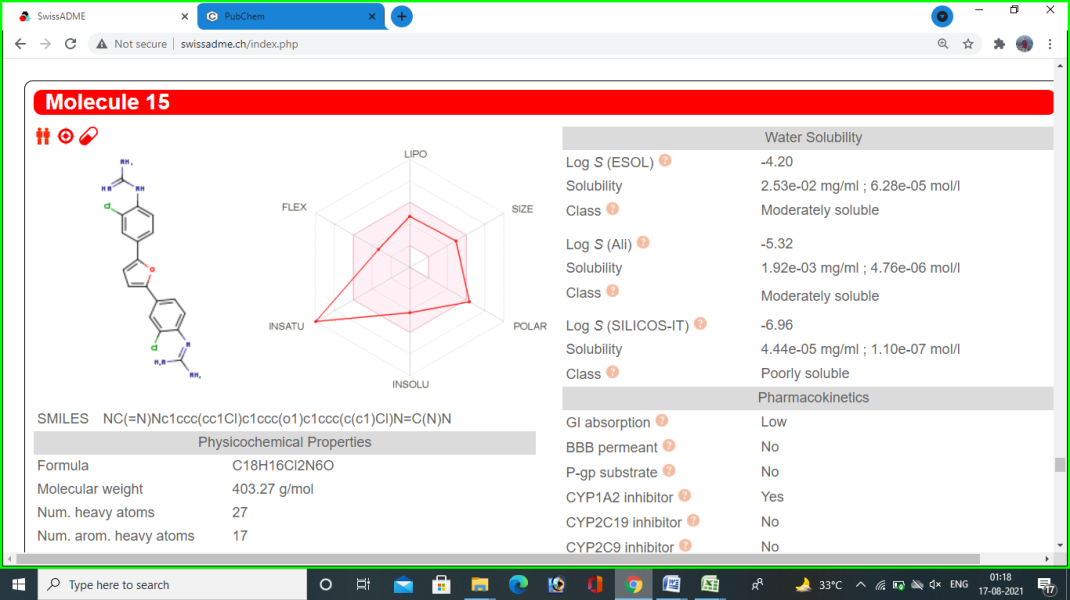 | 1.68 | -4.20 | -6.81 | Yes  0 violation | Low | 0  alert |
|  | **MMV019742** | 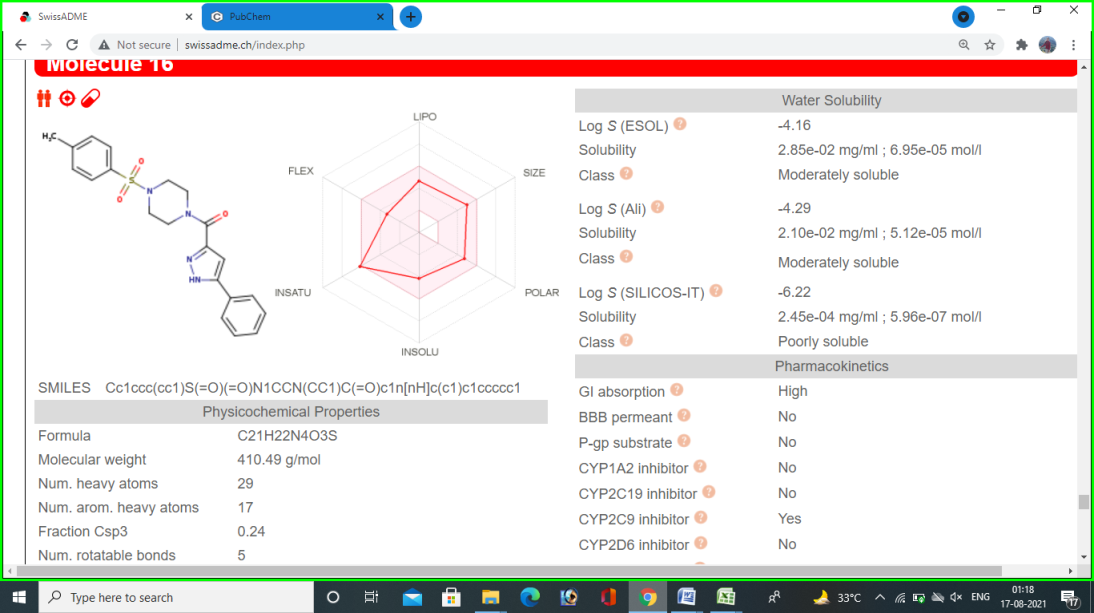 | 2.62 | -4.16 | -6.92 | Yes  0 violation | High | 0  alert |
|  | **MMV099637** | 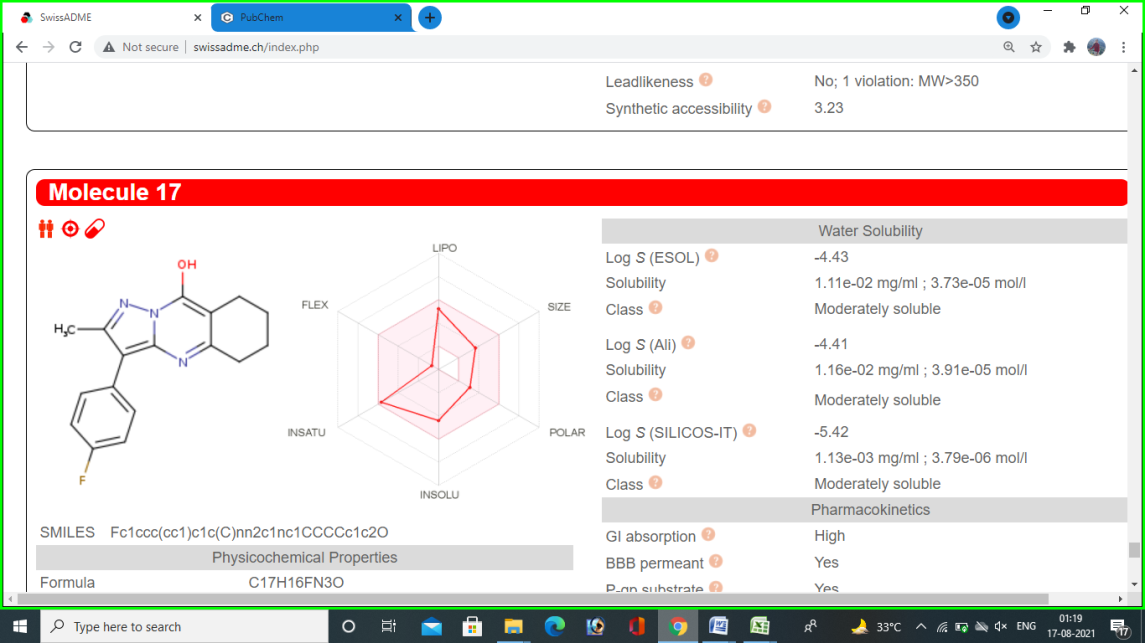 | 3.05 | -4.43 | -5.52 | Yes  0 violation | High | 0  alert |
|  | **MMV022478** | 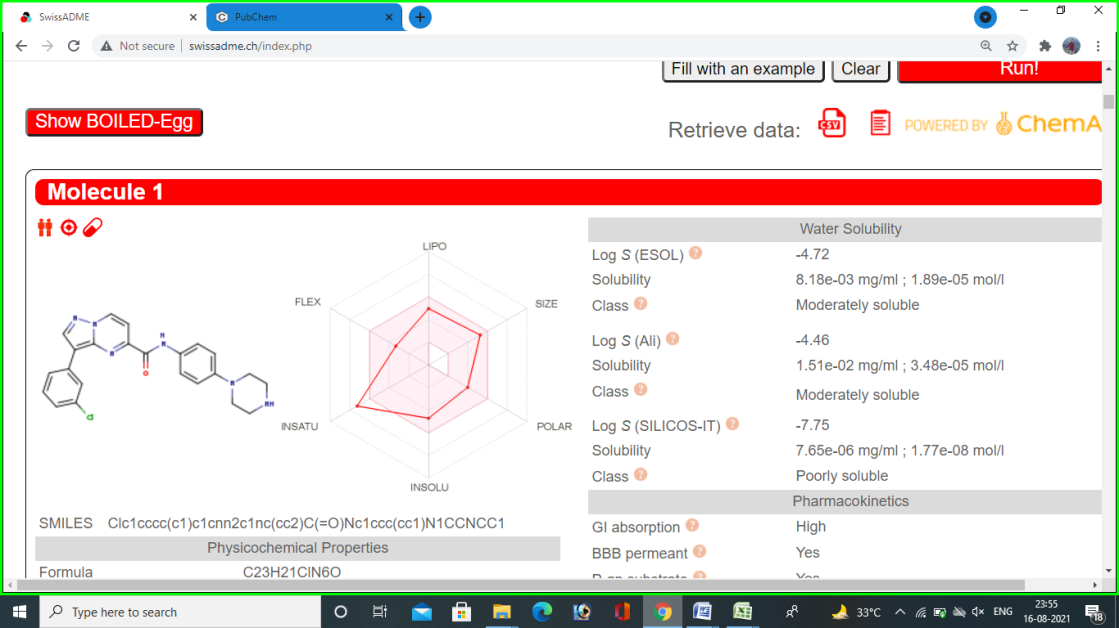 | 2.96 | -4.72 | -6.65 | Yes  0 violation | High | 1 alert |
|  | **MMV687813** | 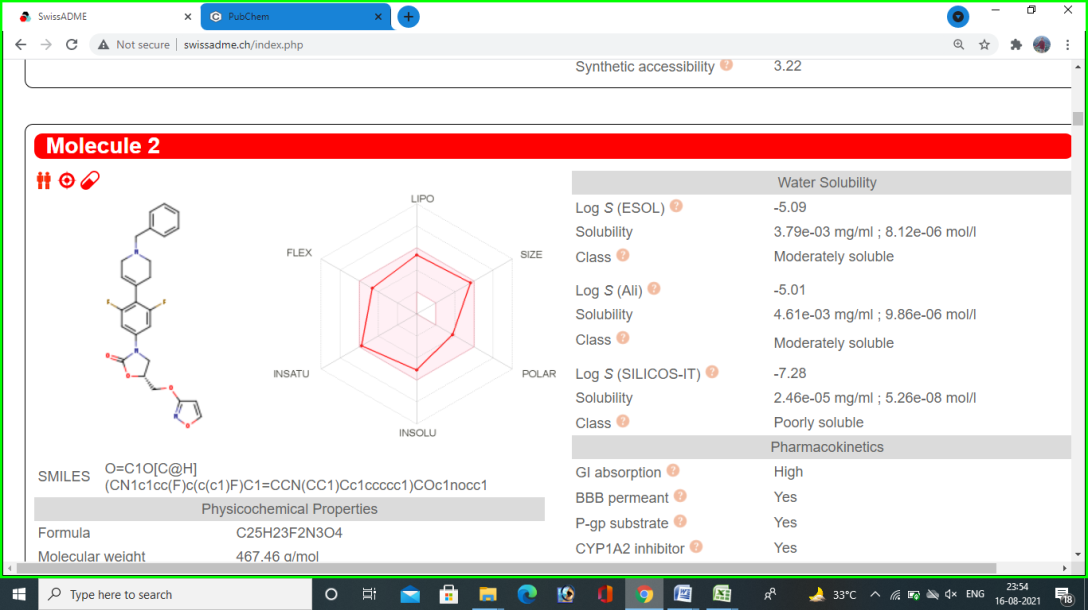 | 4.05 | -5.09 | -6.40 | Yes  0 violation | High | 0  alert |
|  | **MMV688179** | 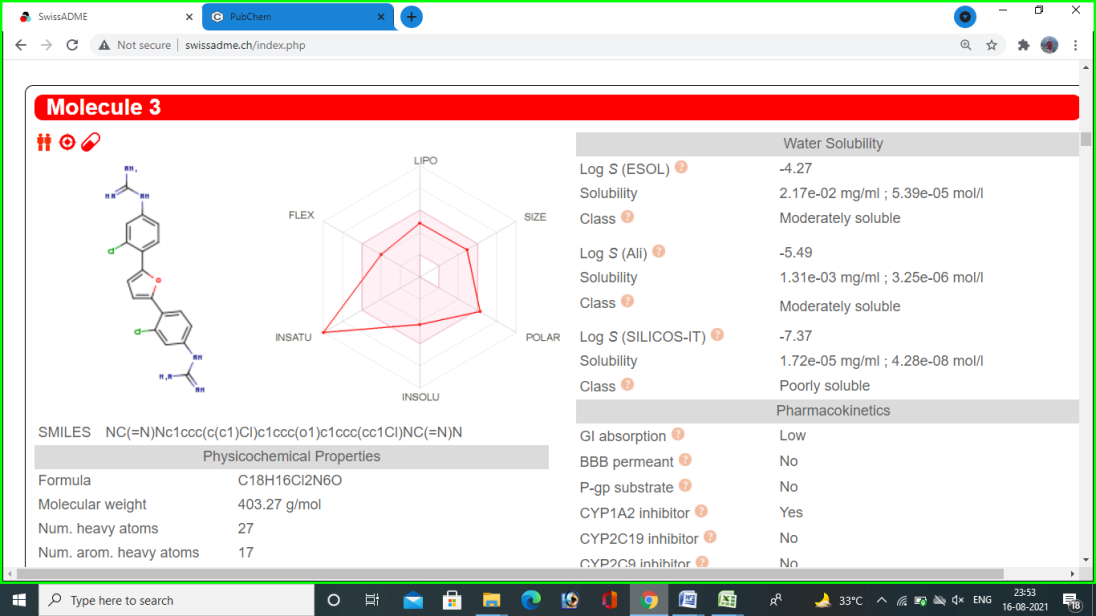 | 1.74 | -4.27 | -6.67 | No, 1 violation | Low | 0  alert |
|  | **MMV688364** | 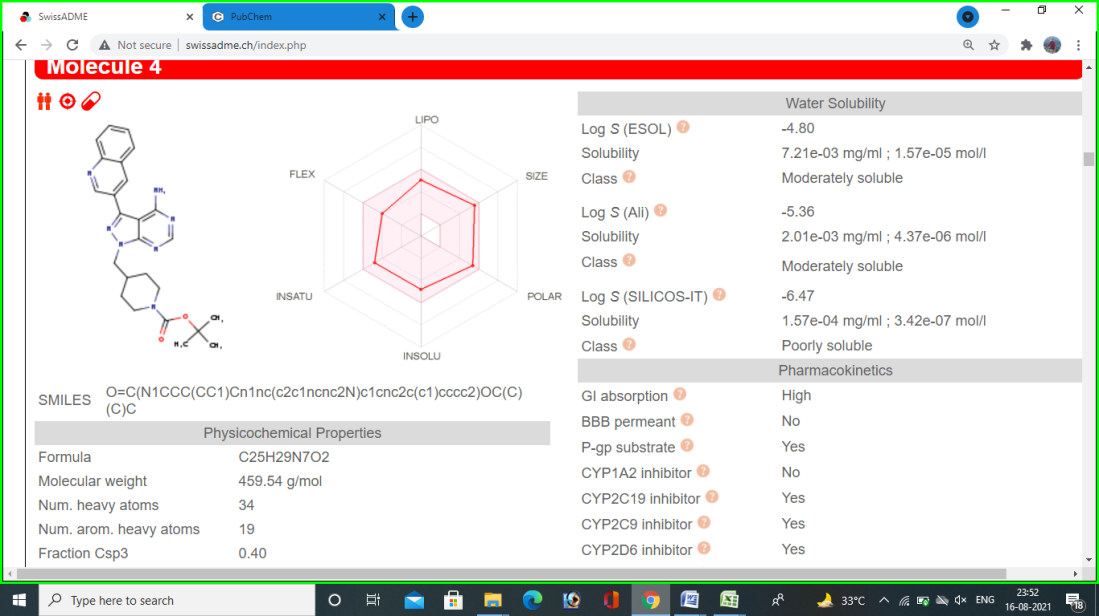 | 4.00 | -4.80 | -6.74 | Yes  0 violation | High | 0  alert |
|  | **MMV688990** | 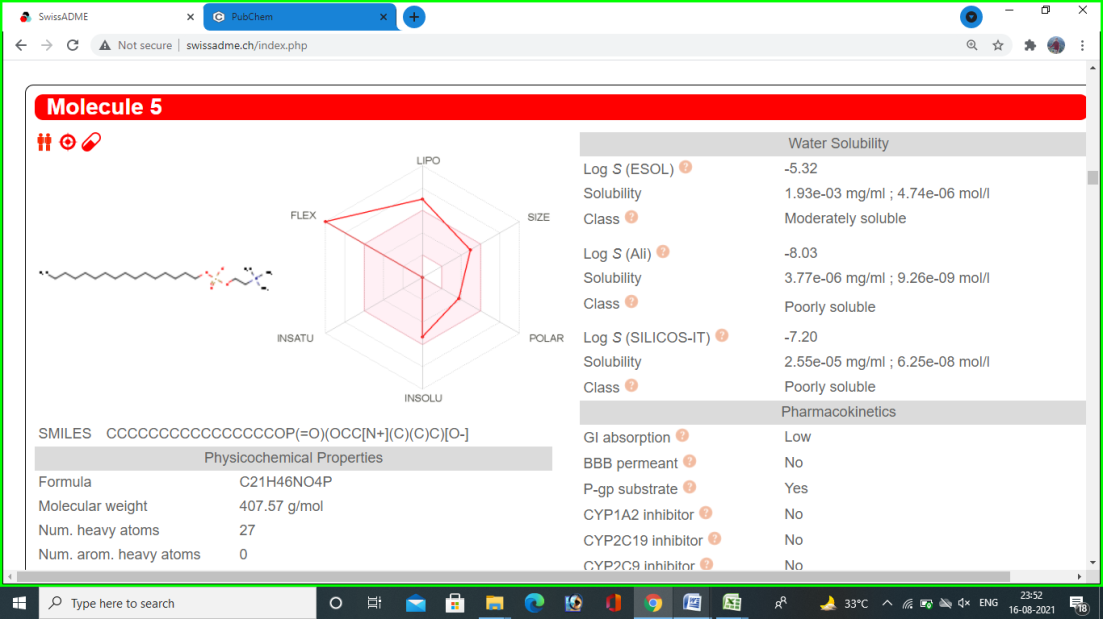 | 0.26 | -5.32 | -3.97 | Yes  0 violation | Low | 0  alert |
|  | **MMV668727** | 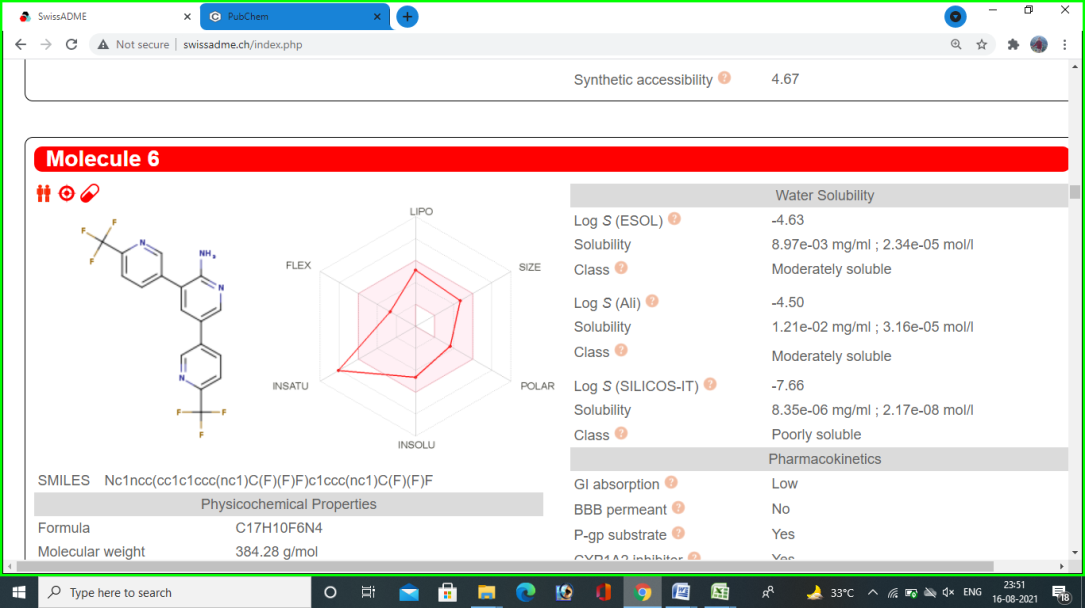 | 2.22 | -4.63 | -6.19 | Yes  0 violation | Low | 0  alert |
|  | **MMV688327** | 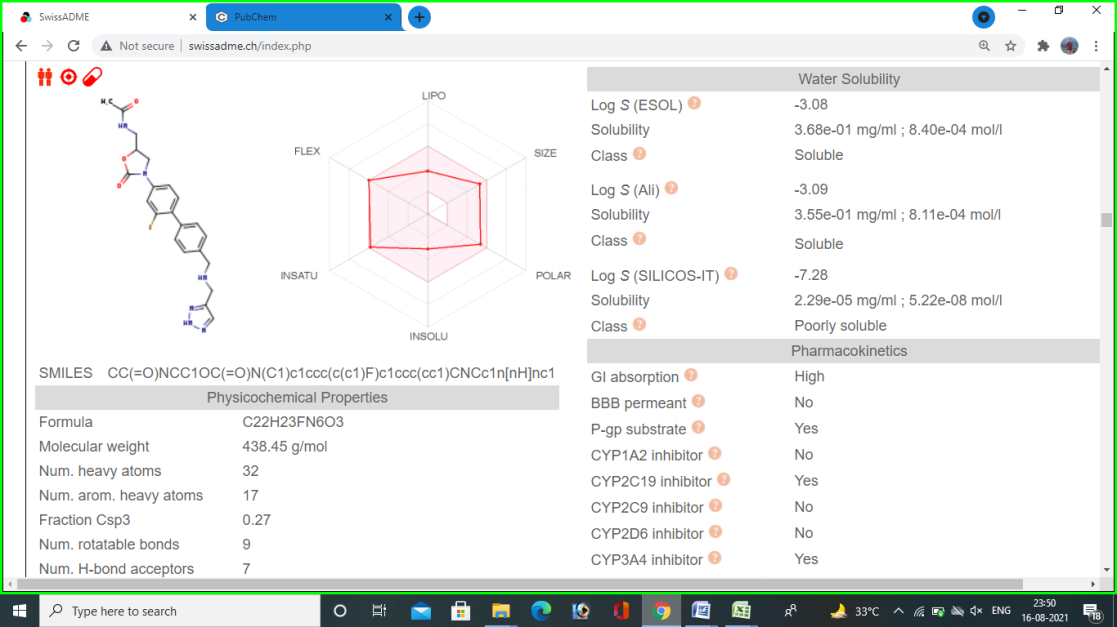 | 2.1 | -3.08 | -8.17 | Yes  0 violation | High | 0  alert |
|  | **MMV688283** | **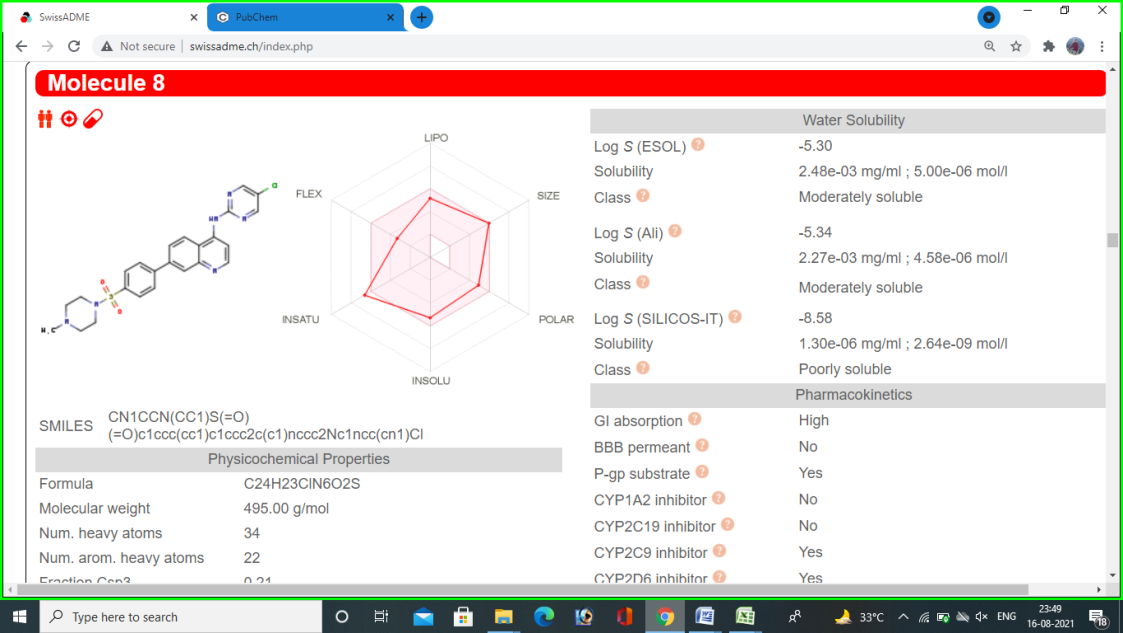** | 3.76 | -5.30 | -6.79 | Yes  0 violation | High | 0  alert |
|  | **MMV688508** | 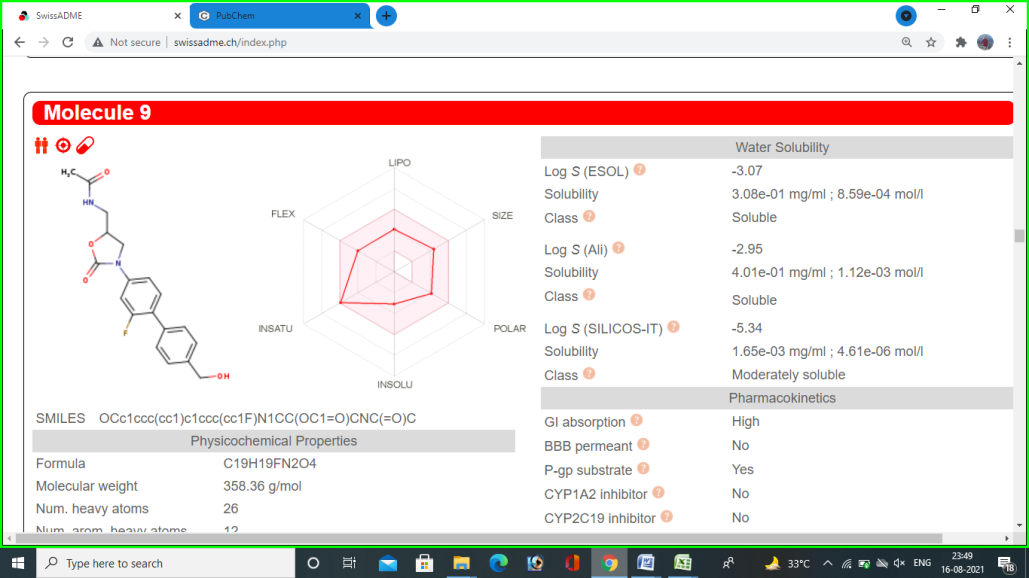 | 2.56 | -3.07 | -7.29 | Yes  0 violation | High | 0  alert |
|  | **MMV688845** | 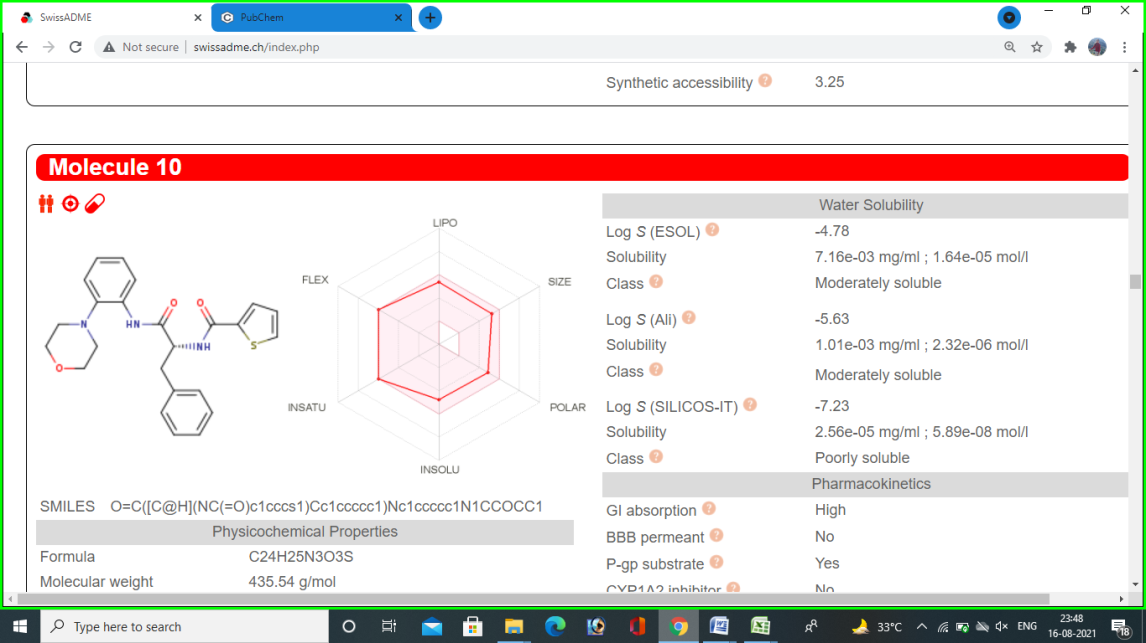 | 3.36 | -4.78 | -6.22 | Yes  0 violation | High | 0  alert |
|  | **MMV688844** | 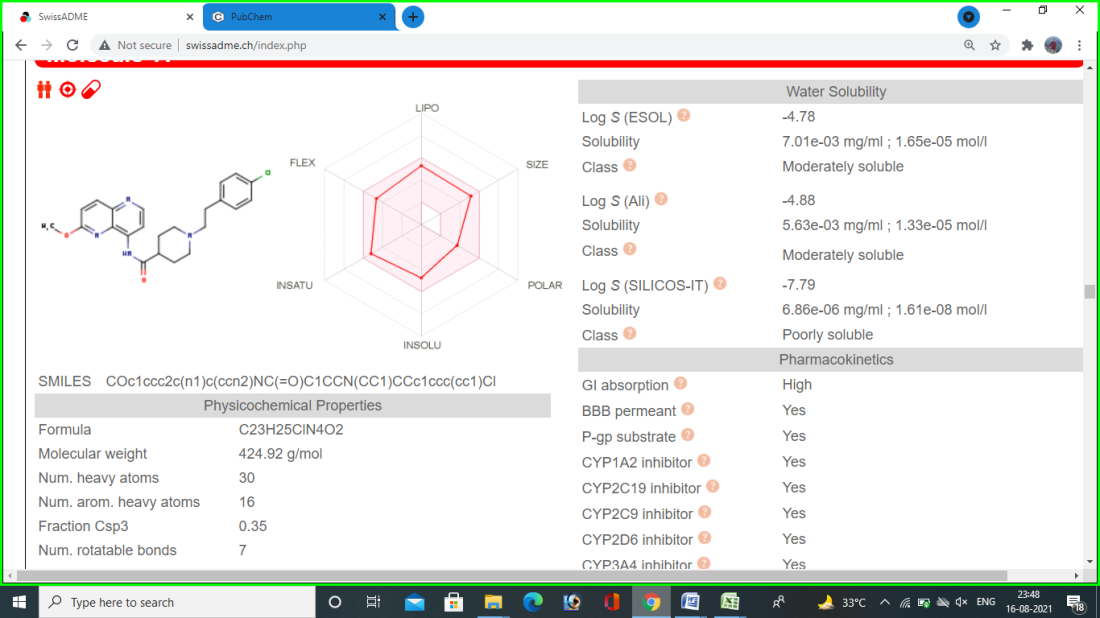 | 4.21 | -4.78 | -6.22 | Yes  0 violation | High | 0  alert |
|  | **MMV1198433** | 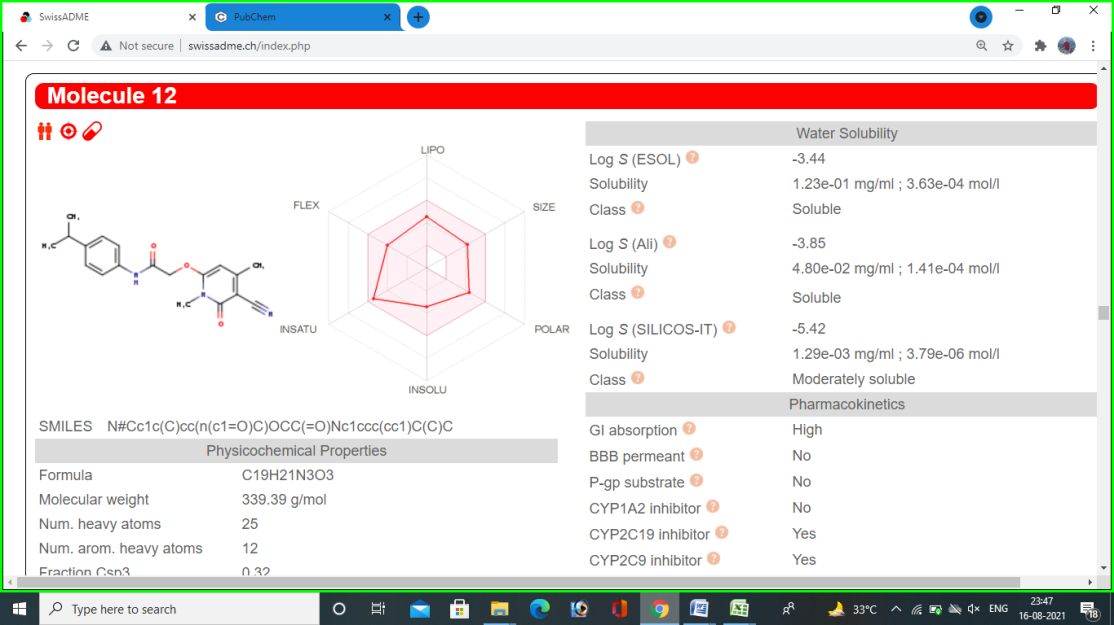 | 3.08 | -3.44 | -6.64 | Yes  0 violation | High | 0  alert |
|  | **MMV407539** | 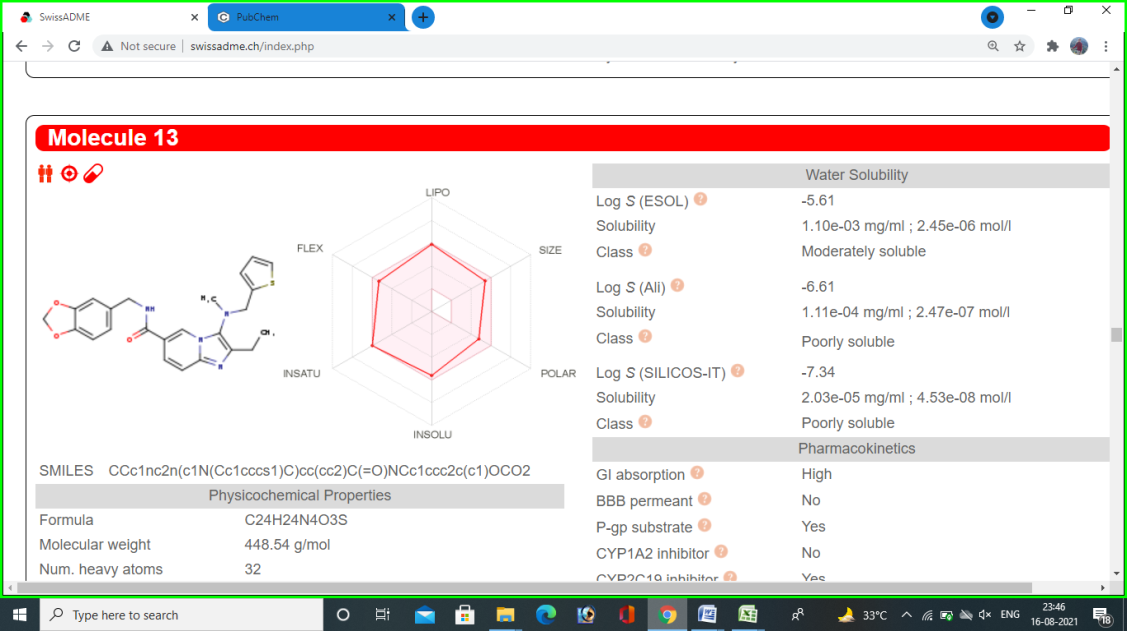 | 3.65 | -5.61 | -5.59 | Yes  0 violation | High | 0  alert |
|  | **MMV675997** | 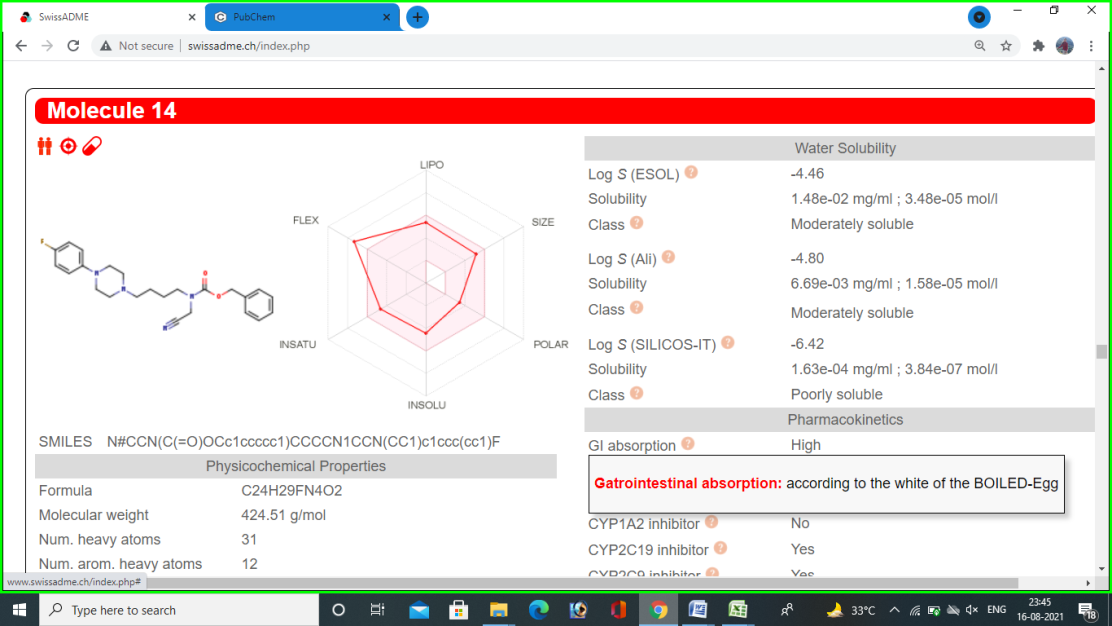 | 4.00 | -4.46 | -6.16 | Yes  0 violation | High | 0  alert |
|  | **MMV689028** | 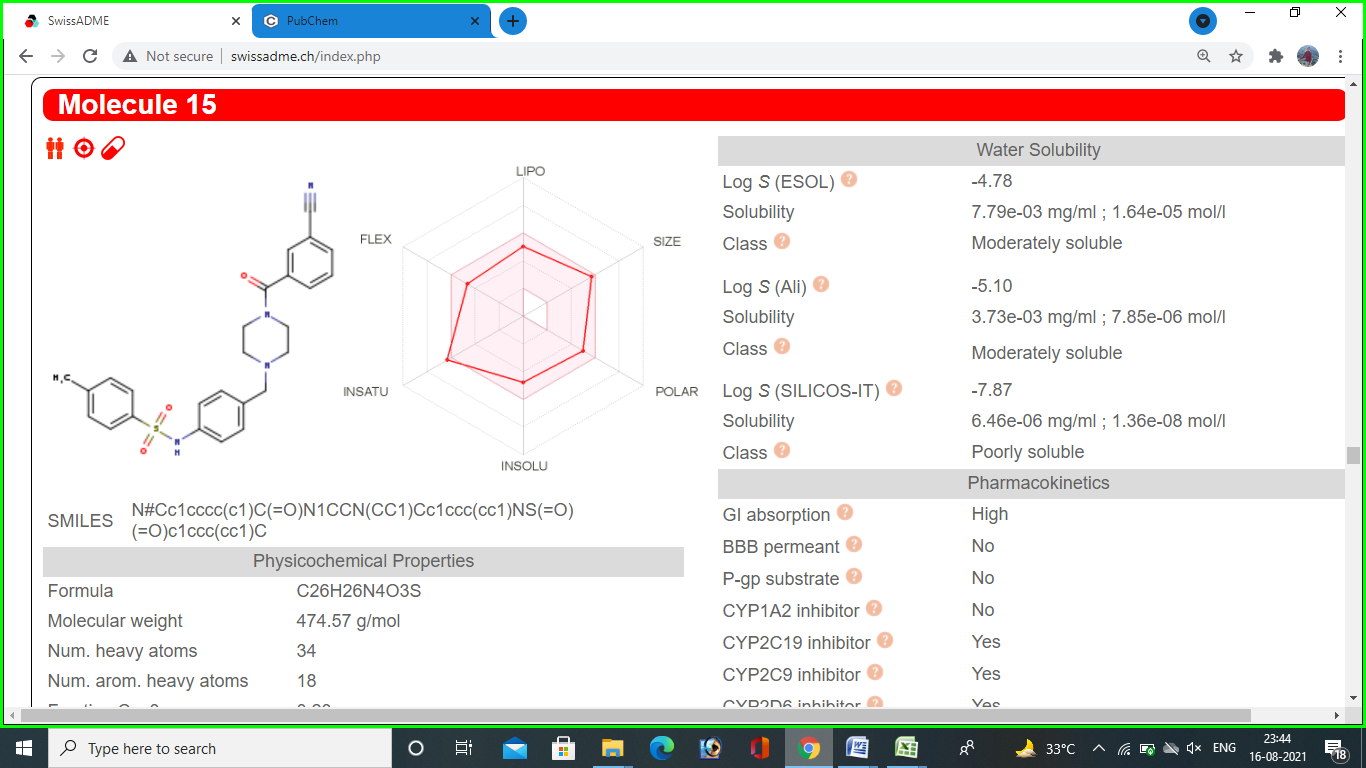 | 3.31 | -4.78 | -6.86 | Yes  0 violation | High | 0  alert |
|  | **MMV026490** | 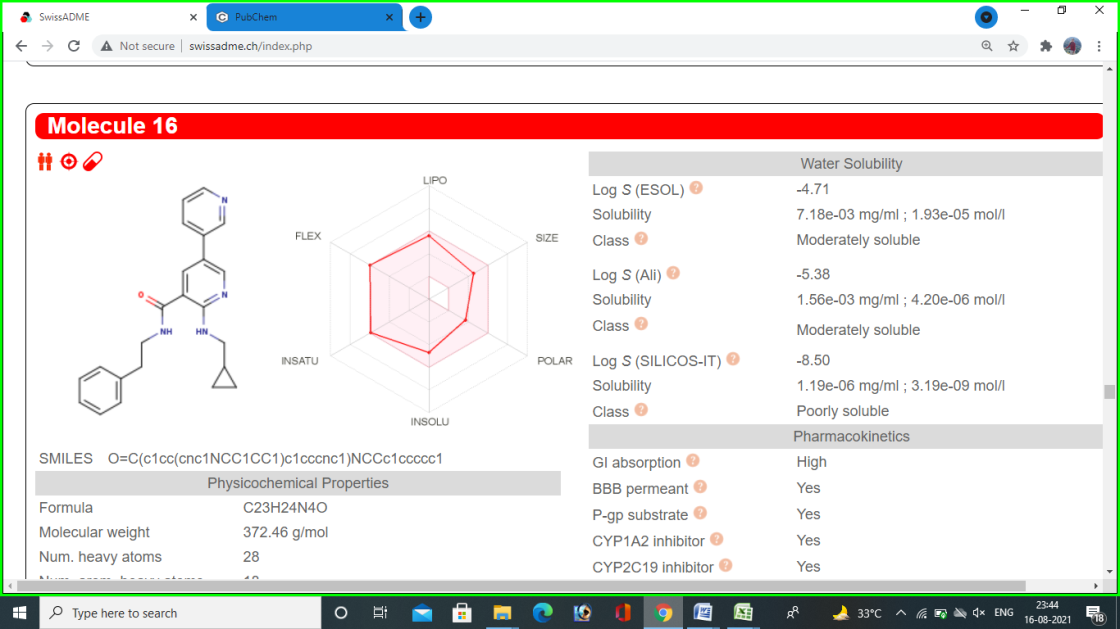 | 2.86 | -4.71 | -5.55 | Yes  0 violation | High | 0  alert |
|  | **MMV671636** | 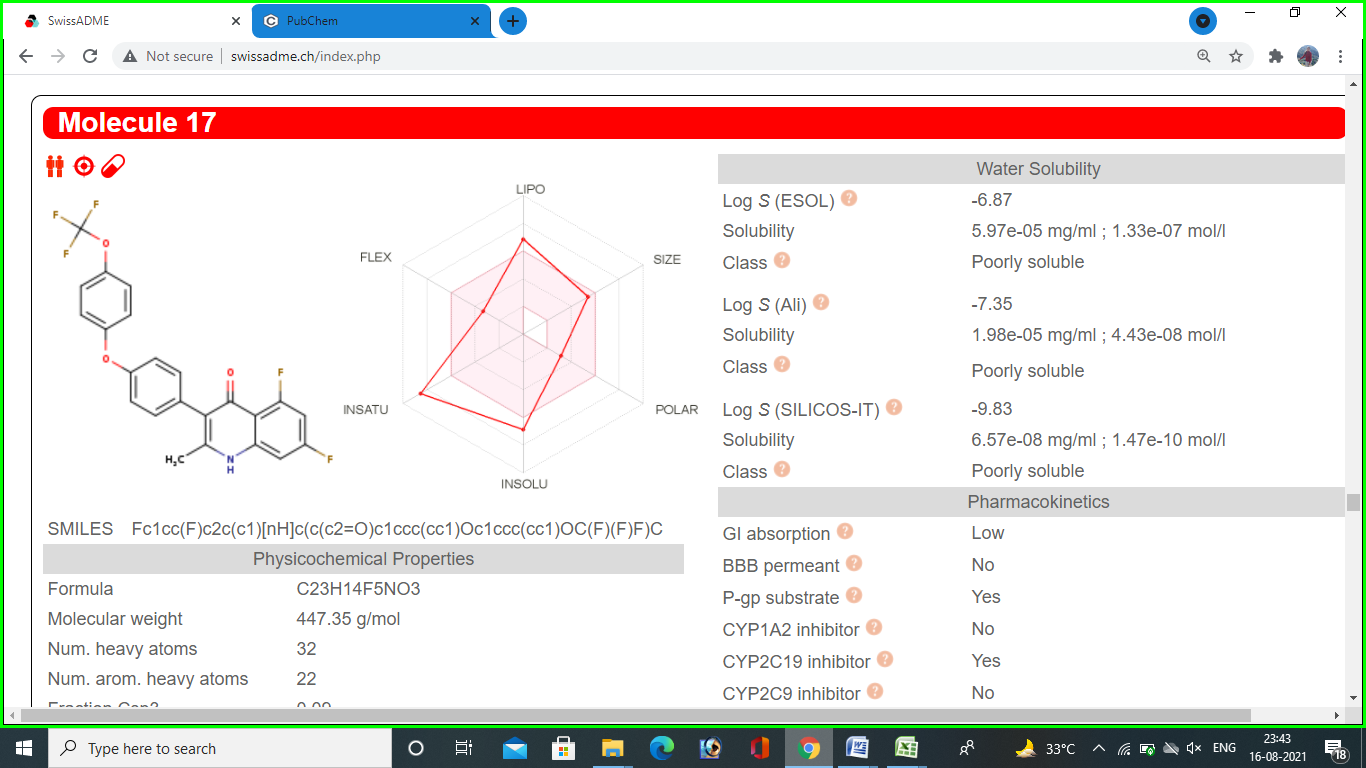 | 3.72 | -6.87 | -4.43 | Yes  0 violation | Low | 0  alert |
|  | **MMV687246** | 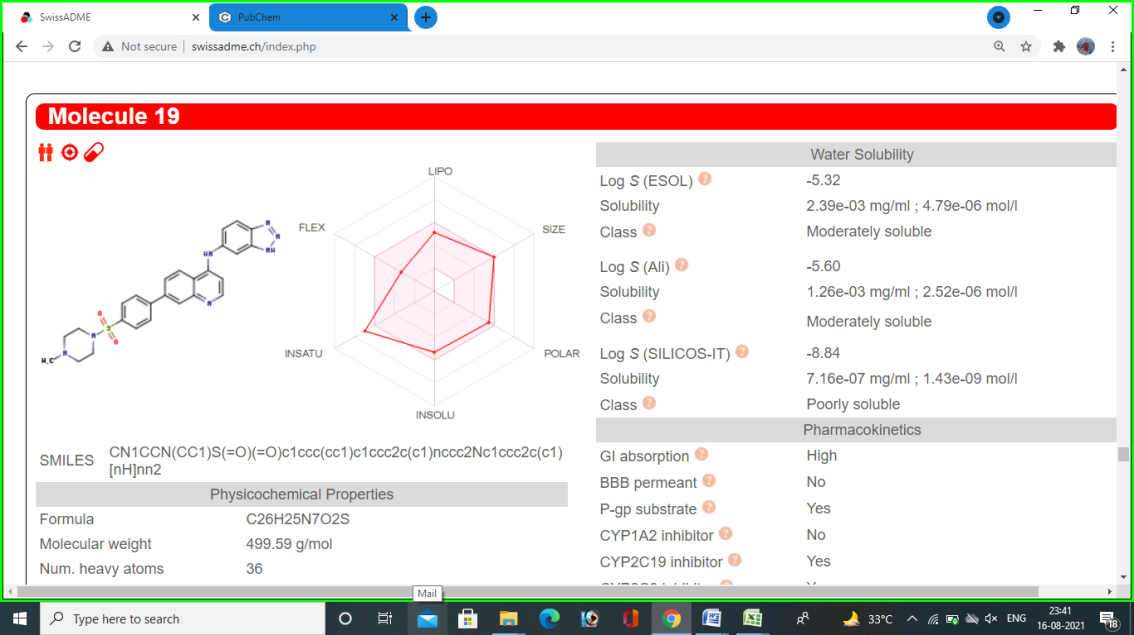 | 2.91 | -5.32 | -6.87 | Yes  0 violation | High | 0  alert |
|  | **MMV688550** | 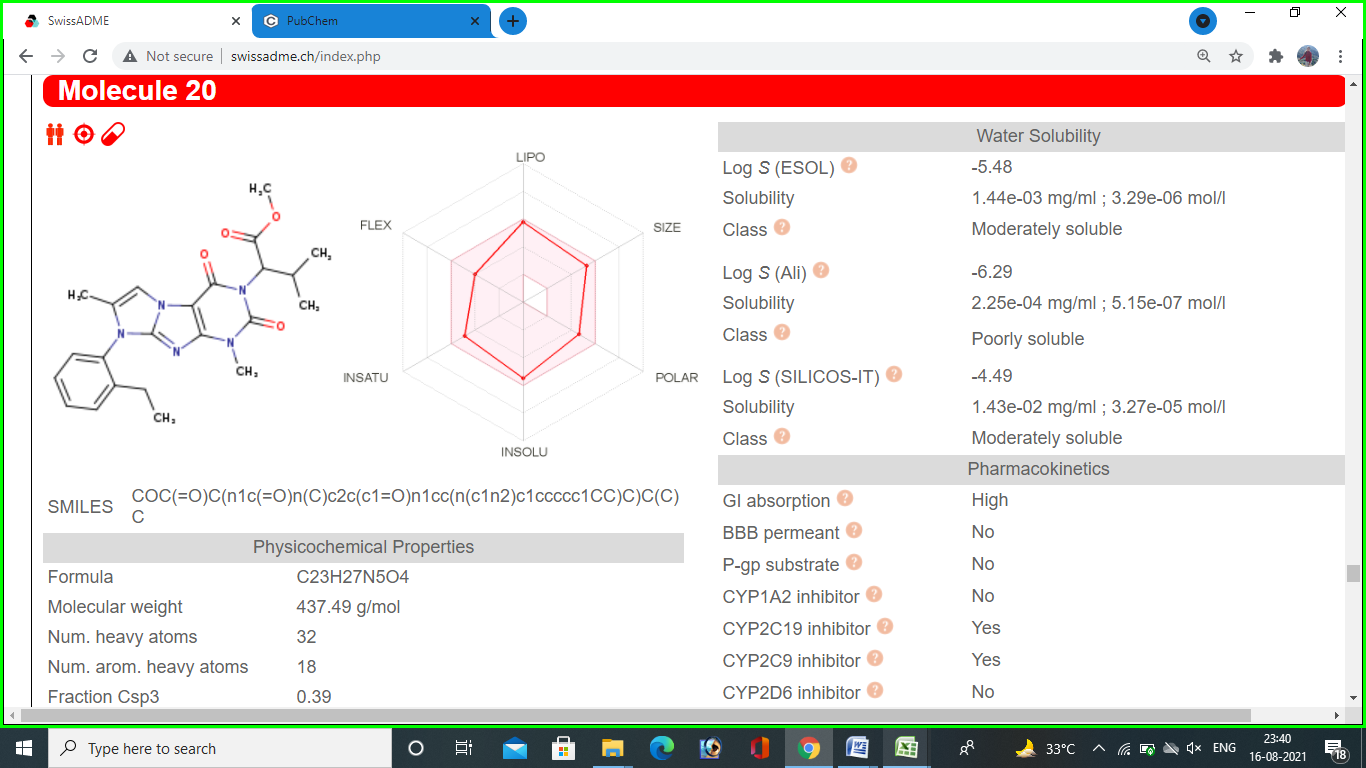 | 4.21 | -5.48 | -5.69 | Yes  0 violation | High | 0  alert |
|  | **MMV688270** | 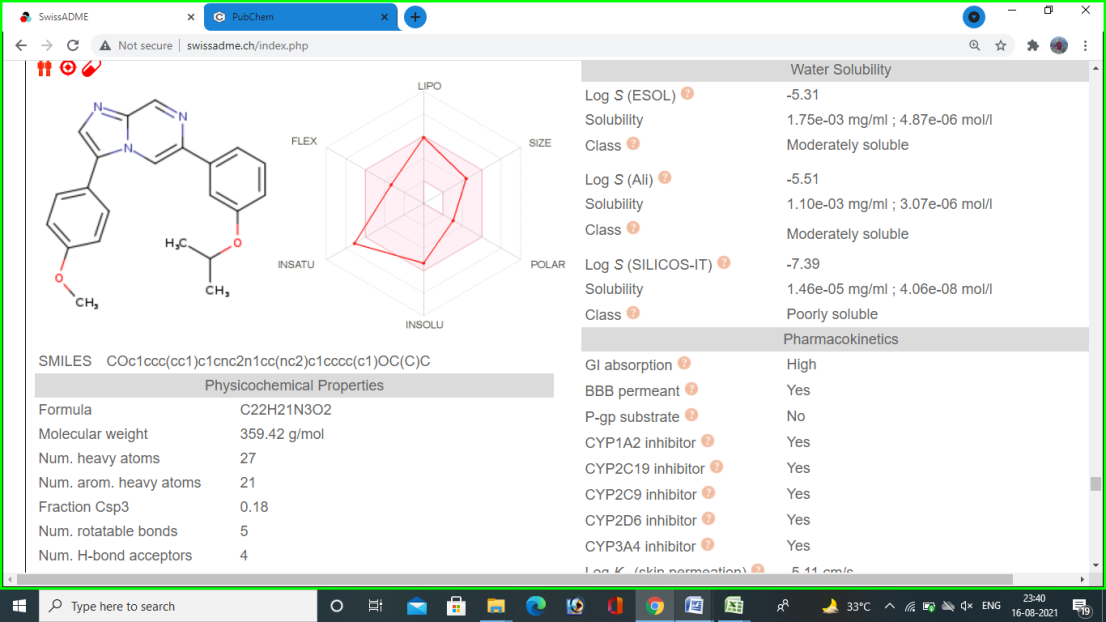 | 3.83 | -5.31 | -5.11 | Yes  0 violation | High | 0  alert |
|  | **MMV020623** | 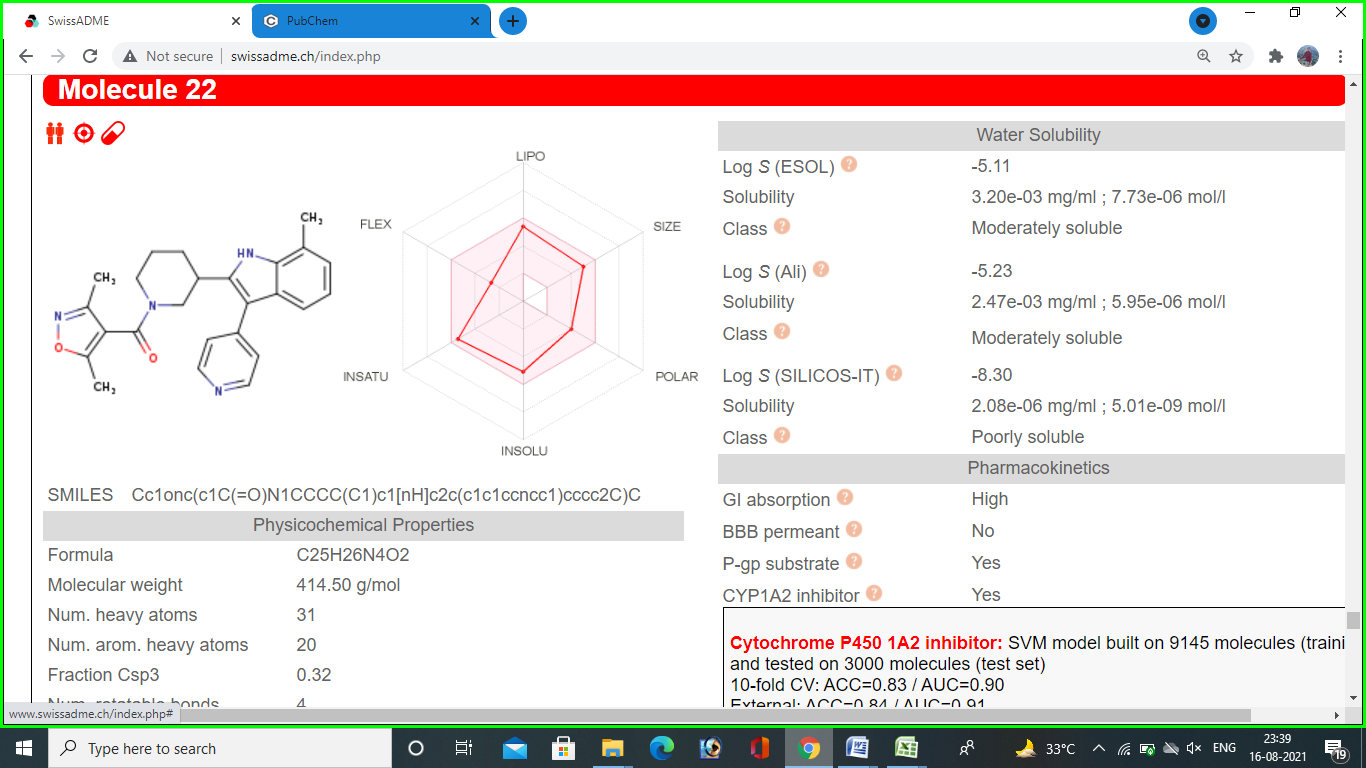 | 3.34 | -5.11 | -6.02 | Yes  0 violation | High | 0  alert |
|  | **MMV688350** | **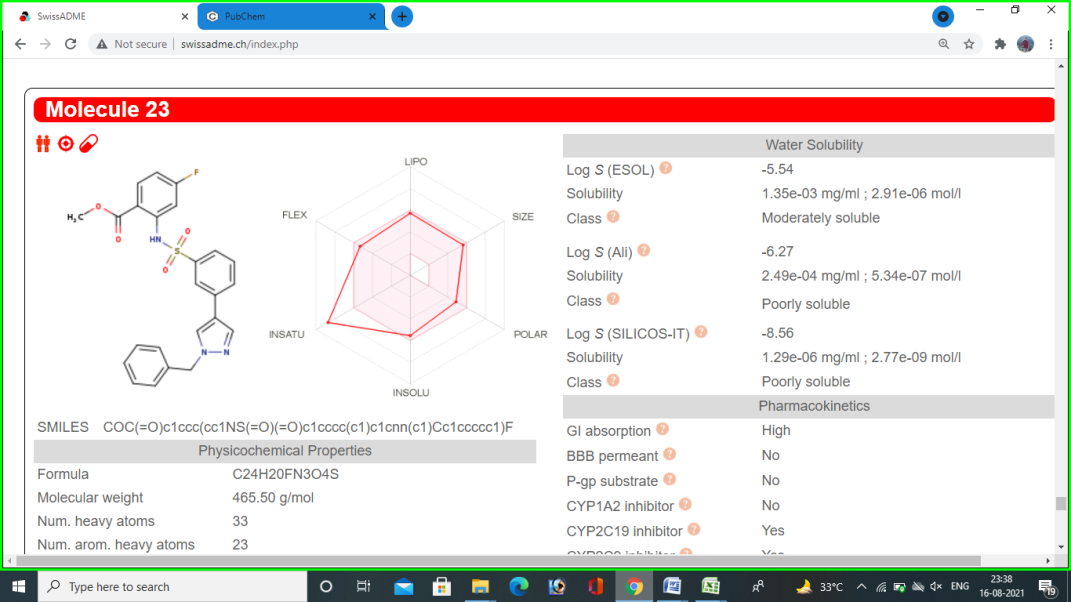** | 3.11 | -5.54 | -5.96 | Yes  0 violation | High | 0  alert |
|  | **MMV023949** | 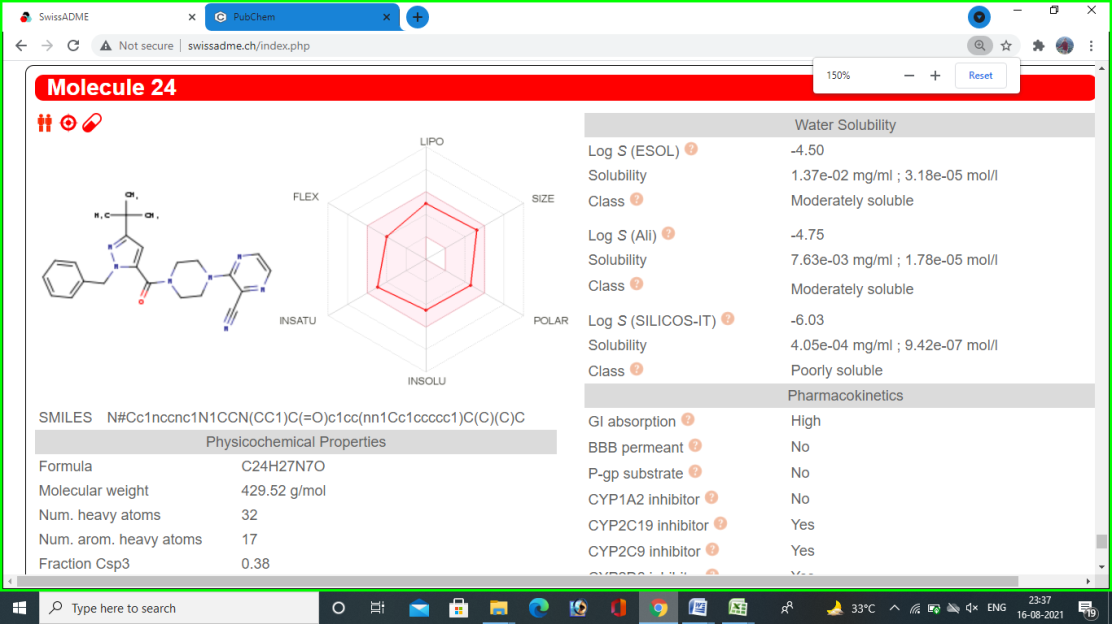 | 3.08 | -4.50 | -6.67 | Yes  0 violation | High | 0  alert |

**The Bioavailability Radar envisages the drug-likeness of a molecule. The pink area represents the optimal range.*

**Predicted octanol/water partition coefficient log Po/w (acceptable range: -0.7 to 5.0).*

**Predicted solubility log S (acceptable range below 6).*

**Predicted skin permeability coefficient log Kp in cm/s.*

***Supplementary Figures***

***Supplementary Figure S1: Uncropped gel images for Figure 2a-b, d-e***

***Figure S1:*** *Full length gel images for commaassie stained SDS gel shown in a) Figure 2a, b) Figure 2b, c) Figure 2d and d) Figure 2e.*

***Supplementary Figure S2: Uncropped images of immunoblotting in Figure 9:***

***Figure S2:*** *Full length images of blot probed with a) anti-ubiquitin and (b) anti-β-acttin. The box indicates the cropped position of membrane in the figure.*

***Supplementary Figure S3: Assessing the cytoxicity of MMV676603 and MMV688704 against mammalian cell lines.***

***Figure S3: Cell viability evaluation using MTT assay:*** *Graphical representation of the percent cell viability of HEK-293T (green) and HepG2 (orange) cells treated with varied concentration of a) MMV676603 and b) MMV688704.*
